# Risk Factors for Brain Metastases in Patients With Small Cell Lung Cancer: A Systematic Review and Meta-Analysis

**DOI:** 10.3389/fonc.2022.889161

**Published:** 2022-06-10

**Authors:** Haiyan Zeng, Danyang Zheng, Willem J. A. Witlox, Antonin Levy, Alberto Traverso, Feng-Ming (Spring) Kong, Ruud Houben, Dirk K. M. De Ruysscher, Lizza E. L. Hendriks

**Affiliations:** ^1^Department of Radiation Oncology (Maastro), GROW School for Oncology, Maastricht University Medical Center+, Maastricht, Netherlands; ^2^Department of Clinical Oncology, Li Ka Shing Faculty of Medicine, The University of Hong Kong, Hong Kong, Hong Kong SAR, China; ^3^Department of Clinical Oncology, The University of Hong Kong-Shenzhen Hospital, Shenzhen, China; ^4^Department of Clinical Epidemiology and Medical Technology Assessment, GROW—School for Oncology and Developmental Biology, Maastricht University Medical Center+, Maastricht, Netherlands; ^5^Department of Radiation Oncology, Gustave Roussy, Villejuif, France; ^6^Université Paris-Saclay, Faculté de Médecine, Le Kremlin-Bicêtre, France; ^7^Department of Pulmonary Diseases, GROW School for Oncology and Developmental Biology, Maastricht University Medical Center+, Maastricht, Netherlands

**Keywords:** small cell lung cancer, brain metastasis, risk factors, systematic review, meta-analysis

## Abstract

The use of prophylactic cranial irradiation (PCI) for small cell lung cancer (SCLC) patients is controversial. Risk factors for brain metastasis (BM) development are largely lacking, hampering personalized treatment strategies. This study aimed to identify the possible risk factors for BM in SCLC.We systematically searched the Pubmed database (1 January 1995 to 18 January 2021) according to the PRISMA guidelines. Eligibility criteria: studies reporting detailed BM data with an adequate sample size (randomized clinical trials [RCTs]: N ≥50; non-RCTs: N ≥100) in patients with SCLC. We summarized the reported risk factors and performed meta-analysis to estimate the pooled hazard ratios (HR) if enough qualified data (i.e., two or more studies; the same study type; the same analysis method; and HRs retrievable) were available. In total, 61/536 records were eligible (18 RCTs and 39 non-RCTs comprising 13,188 patients), in which 57 factors were reported. Ten factors qualified BM data for meta-analysis: Limited stage disease (LD) (HR = 0.34, 95% CI: 0.17–0.67; P = 0.002) and older age (≥65) (HR = 0.70, 95% CI: 0.54–0.92; P = 0.01) were associated with less BM; A higher T stage (≥T3) (HR = 1.72, 95% CI: 1.16–2.56; P = 0.007) was a significant risk factor for BM. Male sex (HR = 1.24, 95% CI: 0.99–1.54; P = 0.06) tended to be a risk factor, and better PS (0–1) (HR = 0.66, 95% CI: 0.42–1.02; P = 0.06) tended to have less BM. Smoking, thoracic radiotherapy dose were not significant (P >0.05). PCI significantly decreased BM (P <0.001), but did not improve OS in ED-SCLC (P = 0.81). A higher PCI dose did not improve OS (P = 0.11). The impact on BM was conflicting between Cox regression data (HR = 0.59, 95% CI: 0.26–1.31; P = 0.20) and competing risk regression data (HR = 0.74, 95% CI: 0.55–0.99; P = 0.04). Compared to M0–M1a, M1b was a risk factor for OS (P = 0.01) in ED-SCLC, but not for BM (P = 0.19). As regular brain imaging is rarely performed, high-quality data is lacking. Other factors such as N-stage and blood biomarkers had no qualified data to perform meta-analysis. In conclusion, younger age, higher T stage, and ED are risk factors for BM, suggesting that PCI should be especially discussed in such cases. Individual patient data (IPD) meta-analysis and well-designed RCTs are needed to better identify more risk factors and further confirm our findings. **Systematic Review Registration:** https://www.crd.york.ac.uk/prospero/display_record.php?ID=CRD42021228391, identifier CRD42021228391.

## Introduction

Small cell lung cancer (SCLC) accounts for about 13% of newly diagnosed lung cancers worldwide (1). Brain metastases (BM) are a very common metastatic site in SCLC: more than 10% of patients have BM at initial diagnosis, more than 50% will develop BM within 2 years, and up to 80% of all patients are found to have BM at autopsy (2). Patients with SCLC and BM have a dismal survival rate, with a 2-year survival rate below 2% (3). Furthermore, BM have a negative impact on the quality of life (QoL). Prophylactic cranial irradiation (PCI) significantly reduces the incidence of BM in patients with SCLC (4, 5). However, because of potential neurotoxicity (6, 7) and possible limited survival, especially in metastatic SCLC (8, 9), PCI is increasingly questioned. Additionally, stereotactic radiosurgery (SRS) has become more available and may represent an attractive therapeutic alternative (10). As a consequence, SCLC guidelines encourage shared decision making regarding PCI for particular subgroup of patients, such as the elderly, very early stages, or extensive stage disease (ED) (11, 12), However, shared decision making is hampered by the fact that risk factors for BM development are largely unknown in SCLC patients. The specific risk of BM (high vs low) could also be used as a stratification factor to better control confounders in trials evaluating BM prevention strategies such as PCI. Therefore, we performed a systematic review and meta-analysis to summarize the possible risk factors for BM in patients with SCLC to support better management of SCLC patients and a better design of SCLC randomized controlled trials (RCTs).

## Methods

### Study Design and Data Extraction

We conducted this study according to the PRISMA guideline (Preferred Reporting Items for Systematic Reviews and Meta-Analyses) (13) and registered it with PROSPERO (CRD42021228391) (14). We performed a systematic literature search in the PubMed database from 1 January 1995 to the search date (18 January 2021), adhering to the PICO method (15) (**Appendix Table 1**). The description of these components is presented in (**Appendix Table 2**). The study eligibility criteria were as follows: 1. SCLC patients without baseline BM; 2. with detailed BM data; 3. had adequate sample size (defined as: retrospective studies or prospective observational/single arm studies [non-RCTs]: N ≥100 patients; RCTs: N ≥50). The detailed criteria are shown in **Appendix Table 3**. We assessed the “risk of bias” for BM in eligible RCTs using the Revised Cochrane risk-of-bias tool for randomized trials (RoB2) (16, 17). We did not grade non-RCTs separately because of the inherent disadvantages of this type of study.

We extracted data according to our published protocol (14) and reported the following critical items: title, the first author, journal, publication year, study design, recruitment period, sample size, age, performance status (PS), sex, thoracic radiotherapy (TRT), surgery, chemotherapy, PCI, follow-up time, statistical analysis, the results of possible risk factors for BM and OS (numbers of events/patients, hazard ratio [HR], 95% CI, and p-value), and conclusion. We also reported the following items for each RCT: brain magnetic resonance imaging (MRI) or computed tomography (CT) at baseline and before PCI; scheduled brain CT or MRI during follow-up; brain imaging contrast-enhanced or not; BM as primary or secondary outcome. We applied the Web Plot Digitizer (18) to extract survival data from plots if necessary.

Two investigators (HZ and DZ) independently screened the titles, abstracts, methods, and full texts for eligibility; extracted data; and assessed the risk of bias. Any conflicts in each step were resolved through discussion with a third investigator (LH).

### Statistical Analysis

Our primary endpoint was BM. When such data were available, we also analyzed OS to further interpret the clinical significance. The effect of the factors on BM and OS was expressed as an HR, being the most appropriate metric for summarizing time-to-event data (19). We first analyzed each factor for BM per study. If two or more studies investigated the factor’s impact on BM with homogenous methodology and outcomes, we performed a meta-analysis with Rev Man 5.4.1 using the EXP[(O − E)/Var] method. If the OS data were not available in one or more studies that were included for the BM meta-analysis, the meta-analysis for OS would not be performed to avoid missing outcome bias. To minimize bias, we used the adjusted rather than the univariate HR if possible. We calculated the observed (O) minus expected (E) number of events and its variance (V) for each study according to the methods of Tierney et al. (20). If similar data were reported by researchers from the same group, only the latest one was included for meta-analysis to avoid data overlapping. Meta-analysis was performed separately for RCTs and non-RCTs to avoid misleading conclusions. A meta-analysis of non-RCTs was not performed if there were sufficient RCTs addressing this issue (21). We used I^2^ to quantify inter-study heterogeneity, of which 25, 50, and 75% can be considered low, moderate, and high heterogeneity (22). If I^2^ >50%, we performed a random-effects meta-analysis (23, 24) using R version 4.1.2 with the “meta” package.

## Results

### Study Selection and Quality Assessment

**Figure 1:**
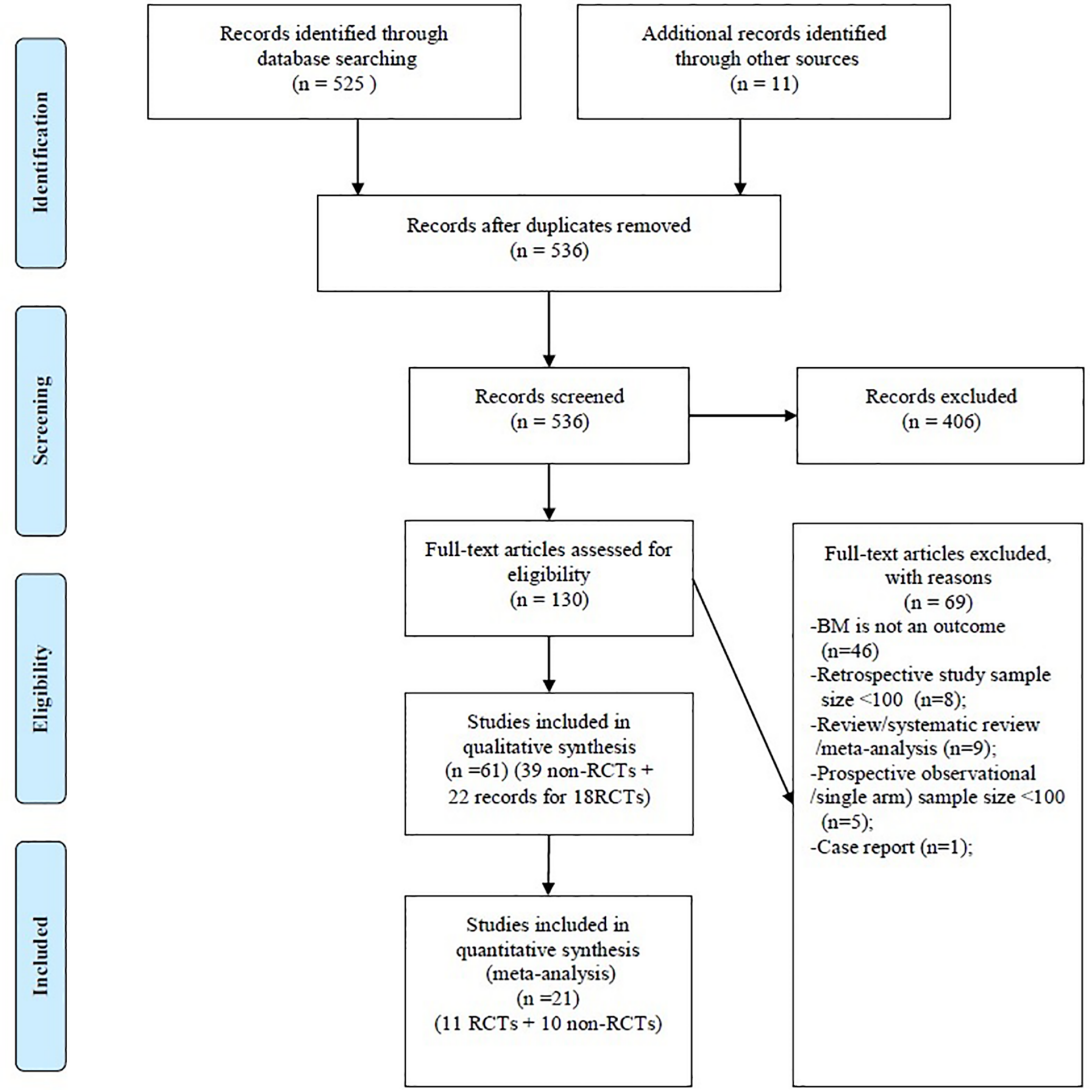
PRISMA flow diagram. BM, brain metastasis; Non-RCTs, non-randomized clinical trials; RCTs, Randomized clinical trials.

**Figure 2:**
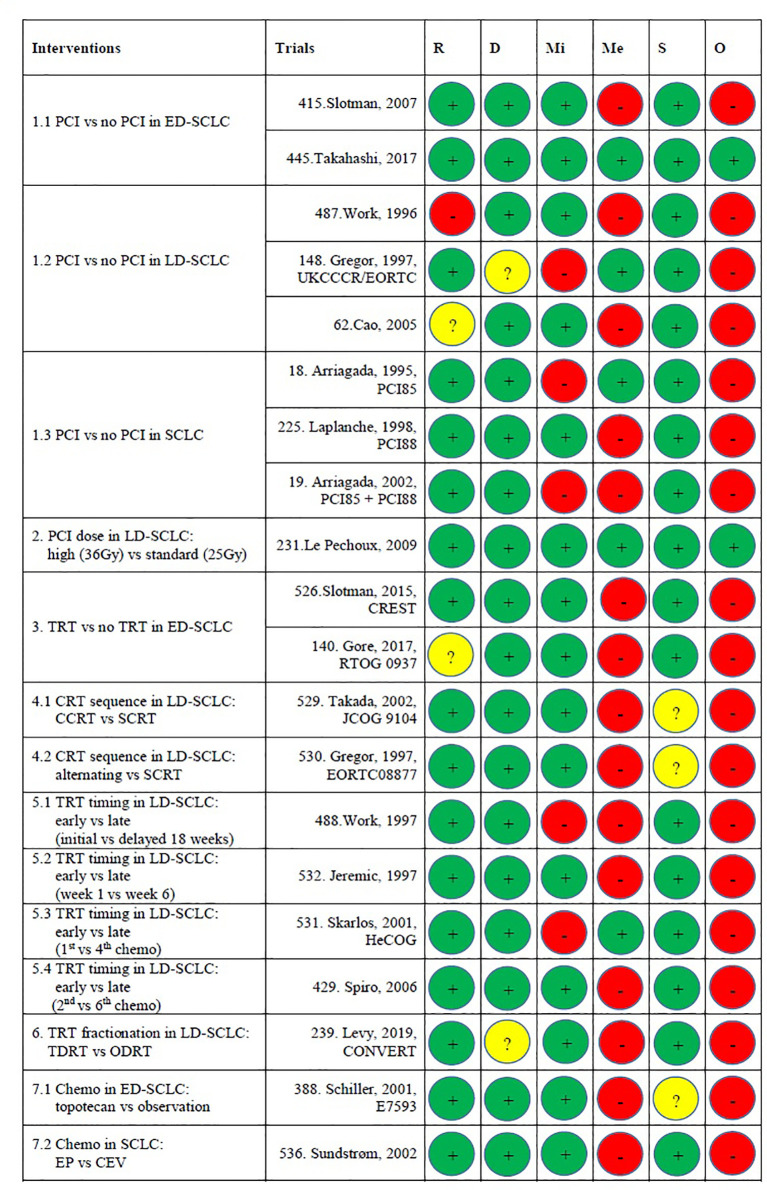
Risk of bias assessments. Risk of bias legend. R, Bias arising from the randomization process; D, Bias due to deviations from intended interventions; Mi, Bias due to missing outcome data; Me, Bias in measurement of the outcome; S, Bias in selection of the reported results; O, Overall risk of bias. Domain 1: Risk of bias arising from the randomization process: The study conducted by Work et al. (34) was at high risk of bias because PCI vs no PCI was not strictly randomized. The study conducted by Cao et al. had “some concerns” because of no information about the random allocation sequence. RTOG 0937 had “some concerns” because baseline age was unbalanced between arms (P = 0.03). The other 16 studies were assessed as at low risk of bias. Domain 2: Risk of bias due to deviations from the intended interventions (effect of assignment to intervention): The CONVERT trial was assessed to have “some concerns” because it is unclear whether there were deviations from the intended intervention that arose because of the trial context. The UKCCCR/EORTC trial was assessed to have “some concerns” since there were deviations from the intended intervention that arose because of the trial context. The others were at low risk. Domain 3: Missing outcome data: This domain is difficult to tell because most trials did not have a regular brain CT/MRI scan plan during the follow-up. In the trials that did have a pre-planned brain CT/MRI scan schedule, only one trial (IPC85) mentioned the compliance at some time point. Readers do not know how many data were missing. The UKCCCR/EORTC trial and HeCOG were at high risk because of no information about missing data. IPC85, the pooled analysis of IPC85+ IPC88, and the study conducted by Work et al. (35) were at high risk because many data were missing but there were no evidence that the result was not biased by missing data. The other 14 studies were at low risk. Domain 4: Risk of bias in measurement of the outcome: 14 studies were judged to be at high risk because the method of measuring the outcome (BM) was inappropriate. They performed brain MRI/CT when patients experience neurological symptoms. The other five trials were at low risk because they had pre-planned brain MRI/CT scan during follow-up. Domain 5: Risk of bias in selection of the reported result: JCOG 9104, E7593, and the trial conducted by Gregor et al. (EORTC) had “some concerns” because of no information about pre-specified analysis plan or selection from multiple eligible analyses. Overall risk of bias: Only the studies conducted by Le Pechoux et al. and Takahashi et al. were judged to be at low risk of bias. The other 17 trials were judged as high risk of bias. This is mainly because of domains 3 and 4. CCRT, concurrent chemoradiotherapy; CEV, cyclophosphamide–epirubicin–vincristine; chemo, chemotherapy; CRT, chemoradiotherapy; ED, extensive-stage disease; EP, etoposide-platinum; LD, limited-stage disease; ODRT, once-daily radiotherapy; PCI, prophylactic cranial irradiation; SCLC, small cell lung cancer; SCRT, sequential chemoradiotherapy; TDRT, twice-daily radiotherapy; TRT, thoracic radiotherapy.

The systematic review identified 536 records, of which 61 records met the inclusion criteria (22 records for 18 RCTs comprising 5,060 patients and 39 non-RCTs comprising 8,128 patients [including two prospective observational studies comprising 544 patients]) (**Figure 1**). All 18 RCTs were published between 1995 and 2019, but only three were from 2010 to 2019 (25–27). As shown in **Appendix Tables 4****,** **5**, BM was the primary endpoint in three trials (5, 28–30). Brain MRI/CT was performed before treatment of patients in two trials (27, 31) and before PCI in six trials (9, 26, 28, 30–32). In five trials, brain CT/MRI was scheduled during follow-up (9, 26, 28, 30, 33) and in one trial [PCI85 (28)], the number of performed CT scans at pre-specified time points was mentioned (which indicated low compliance). As regular brain imaging was not performed in most trials, asymptomatic BM will have been missed, which has resulted in a high risk of bias at domain 4 (measurement method) or domain 3 (missing outcome) according to RoB2. Because of that, two RCTs were assessed to be at low risk of bias, while the others were at high risk of bias (**Figure 2**). The 39 non-RCTs were published from 1995 to 2020, among which 32 were from 2010 to 2020. The study design, characteristics, and treatments of patients are shown in **Appendix Table 6**.

In addition to symptomatic BM, we found that the pre-PCI BM (BM immediately before PCI) was investigated in one study (36) and the first isolated BM event, rather than overall BM during the whole disease course, was analyzed in five studies (37–41). Both the first isolated BM and overall BM were reported in eight papers (28–30, 42–46) and showed that the first isolated BM incidence was lower than the overall BM incidence (**Table 1**). We only performed meta-analysis for overall BM because this is more relevant than a first isolated BM event.

**Table 1:** Risk factors for BM in SCLC.

Risk factors	Studies ID	First Author (Trial)	Statistics	BM Results^A^	OS results^B^	Conclusion	Comments
***A. Baseline characteristics***							
1. Age							
1) <70 *vs* ≥70: Meta-analysis for BM is not applicable because of different statistics							
	115	Farooqi, 2017 (1)	BM: Competing-risk regression. OS: Cox proportional hazard regression	<70 *vs* ≥70: SHR 1.07, 95% CI 0.71–1.62, P= 0.734;	HR 1.34, 95% CI 1.08–1.66, P=0.007; Multivariate (adjusted factors: NI): P>0.05	Age is not an independent risk factor for BM or OS in LD-SCLC	Two definitions for time to development of BM, unclear which one is used
	34	Bernhardt, 2017 (2)	Cox proportional hazard regression	<70 *vs* ≥70: HR 0.90, 95% CI 0.34-2.33, P= 0.83;	<70 *vs* ≥70: HR 1.47, 95% CI 0.28-2.45, P= 0.13;	Age is not a significant risk factor for BM or OS in ED-SCLC with PCI	No report of patients distribution in each group
2) <65 vs ≥ 65: 3 studies (376, 439, 203) have qualified BM data to perform meta-analysis, no qualified data for OS meta-analysis							
	376	Sahmoun, 2004 (3)	Cox proportional hazard regression.	≥ 65 vs <65 (adjust for hypertension, sex, BMI, laterality): HR=1.59, 95%CI: 1.03-2.5; P: NI.	NI	Compared to age ≥ 65, age <65 is an independent risk factor for BM in SCLC.	Investigated only demographic factors, did not consider tumor and treatment related factors
	520	Zhu, 2014 (4)	Cox proportional hazard regression.	<65 vs ≥65: p=0.802	<65 vs ≥65 (adjust for PS, stage, LVI, and BM): HR=1.798, 95%CI: 1.027-3.148; P=0.04.	Compared to age <65, age ≥65is an independent risk factor for OS in resected LD-SCLC, but not for BM.	BM was included in the multivariate model of OS
	439	Suzuki, 2018 (5)	Cox proportional hazard regression.	≤ 64 vs > 64: HR: 0.846, 95%CI: 0.584–1.225; P= 0.375.	NI	Age is not a significant risk factor for BM in SCLC	
	203	Kim, 2019 (6)	Cox proportional hazard regression.	<65 vs ≥65: HR=0.418, 95%CI: 0.187–0.938, P=0.034; adjust for Sex, T, and PCI: P=0.037.	P>0.05	Compared to age ≥ 65, age <65 is a risk factor for BM in LD-SCLC, but not for OS.	Inverse probability treatment weight (IPTW) was used to minimize bias; No report of patients distribution in each group after IPTW; Details of multivariate model not reported.
3) <60 vs ≥60: Meta-analysis for BM is not applicable because of different statistics and no enough HR data							
	514	Zeng, 2017 (7)	Cox proportional hazard regression.	BM: <60 : 24/117 (20.5%); ≥60: 12/58 (20.7%); HR=1.07, 95%CI: 0.53-2.14; p=0.85	NI	Age is not a significant risk factor for BM after PCI in SCLC	
	81	Chen, 2018 (8)	BM: Logistic regression. OS: Cox proportional hazard regression.	<60 vs ≥60 (adjust for sex, PS, tumor load, number of metastatic sites, PCI timing): OR=1.077, 95%CI: 0.428–2.708; P >0.05.	<60 vs ≥60: HR=1.477, 95%CI: 0.823–2.653; P=0.191.	Age is not a significant risk factor for BM or OS in ED-SCLC	Logistic regression was used for BM analysis.
	519	Zheng, 2018 (9)	Cox proportional hazard regression.	<60 vs ≥ 60: HR: NI, 95%CI: NI; p=0.808	P=0.823	Age is not a significant risk factor for BM or OS in LD-SCLC without PCI	Investigated multiple factors (N=21) with limited sample size (n=153).
	513	Zeng, 2019 (10)	Competing-risk regression	<60 vs ≥60: HR=1.20, 95%CI: 0.84-1.71; P=0.32	NI	Age is not a significant risk factor for BM after PCI in SCLC	
4) ≤ 60 vs > 60	139	Gong, 2013 (11)	Cox proportional hazard regression.	≤ 60 vs > 60: HR: NI, 95%CI: NI; P= 0.841.	≤ 60 vs > 60: HR: NI, 95%CI: NI; P= 0.841.	Age is not a significant risk factor for BM or OS in resected LD-SCLC.	Contained many patients with combined SCLC and NSCLC (53.5%, 69/129).
5) <68 vs ≥ 68	377	Sahmoun, 2005 (12)	Cox proportional-hazard regression	≥ 68 vs <68: (adjust for treatment, stage, BMI, sex, laterality, anatomical site, PCI): HR=0.67, 95%CI: 0.41-1.12; P: NI.	≥ 68 vs <68: (adjust for treatment, stage, BMI, sex, laterality, anatomical site): HR=0.62, 95%CI: 0.41-0.95; P: NI.	Compared to age <68, age ≥68 is an independent risk factor for OS in SCLC, but not for BM.	The hazards model of OS did not include PCI.
6) ≤ 58 vs > 58	80	Chen, 2016 (13)	Cox proportional hazard regression	≤ 58 vs > 58: HR, 1.065; 95%CI: 0.722–1.571; p>0.05;	≤ 58 vs > 58: HR, 1.302; 95%CI: 0.898–1.889; p>0.05;	Age is not a significant risk factor for BM or OS in ED-SCLC	
7) <58.5 vs ≥ 58.5	122	Fu, 2014 (14)	Cox proportional-hazard regression	BM as a first recurrence site: ≥ 58.5 vs <58.5 (adjust for sex, PS, stage, CTC at baseline, CTC post-first cycle, CTC post-fourth cycle, response): HR=0.983, 95%CI: 0.953–1.015; P=0.290.	NI	Age is not a significant risk factor for BM after PCI in stage III SCLC	Analyzed BM as a first site of recurrence; No report of patients distribution in each group
8) Continuous: Meta-analysis for BM is not applicable because of different statistics and no HR data							
	491	Wu, 2017 (15)	BM: Competing risk regression; OS: Cox proportional hazard regression	(Continuous) : P>0.05	(Continuous): HR= 1.01; 95%CI: 0.99–1.03; P= 0.23	Age is not a significant risk factor for BM or OS in LD-SCLC	No details on BM results, i.e. HR, 95%CI, and detailed P value.
	28	Bang, 2018 (16)	Cox proportional hazard regression	(Continuous) : P>0.05	(Continuous) : P>0.05	Age is not a significant risk factor for BM or OS in ED-SCLC	Backward stepwise multivariate analysis
	86	Chu, 2019 (17)	Pre-PCI BM: binary logistic regression; OS: Cox proportional hazard regression.	OR=0.976, 95%CI: 0.924–1.032, P=0.400.	HR=1.022, 95%CI: 0.986–1.059, P=0.235	Age is not a significant risk factor for pre-PCI BM or OS in LD-SCLC	Investigated risk factors for Pre-PCI BM in LD-SCLC using logistic regression.
2. Race/ethnicity: Meta-analysis for BM is not applicable because of different statistics							
	115	Farooqi, 2017 (1)	BM: Competing-risk regression. OS: Cox proportional hazard regression	White, non-Hispanic *vs* all others: SHR 1.35, 95%CI: 0.90–2.04; P=0.145;	HR 0.91, 95%CI: 0.71–1.16; P=0.438;	Race is not a significant risk factor for BM or OS in LD-SCLC	Two definitions for time to development of BM, unclear which one is used
	439	Suzuki, 2018 (5)	Cox proportional hazard regression.	White vs non-white: HR: 1.098, 95%CI: 0.677–1.779; P= 0.705.	NI	Race is not a significant risk factor for BM in SCLC	
3. Sex: 5 studies (368, 80, 377, 514, 439) have qualified BM data to perform meta-analysis, no qualified data for OS meta-analysis							
1) LD-SCLC: 368 has available data for meta-analysis							
	520	Zhu, 2014 (4)	Cox proportional hazard regression.	P= 0.906	P= 0.901	Sex is not a significant risk factor for BM or OS in resected LD-SCLC	
	122	Fu, 2014 (14)	Cox proportional-hazard regression	BM as a first recurrence site: male vs female (adjust for age, PS, stage, CTC at baseline, CTC post-first cycle, CTC post-fourth cycle, response): HR= 1.502, 95%CI: 0.751–3.004; P=0.250.	NI	Sex is not a significant risk factor for BM after PCI in stage III SCLC	Analyzed BM as a first site of recurrence; No report of patients distribution in each group; Data overlapped with No.514.
	115	Farooqi, 2017 (1)	BM: Competing-risk regression. OS: Cox proportional hazard regression	Female *vs* male: SHR 1.00, 95%CI: 0.72–1.4; P=0.981	HR 1.09, 95%CI: 0.91–1.30; P=0.345;	Sex is not a significant risk factor for BM or OS in LD-SCLC	Two definitions for time to development of BM, unclear which one is used
	368	Roengvoraphoj, 2017 (18)	BM: log-rank; OS: Cox proportional-hazard regression	Mean BMFS: Female: 96 (95% CI 77–114), Male: 64 months (95% CI 51–75) (HR= 1.79, 95%CI: 1.05–3.04; p = 0.031).	Median OS: 16.8 months (95% CI 14.8–18.9): Female: 20 (95% CI 15–25), Male: 14 (95% CI: 11–17). female vs male (Adjust for PCI, response, chemo regimen, and age) HR= 1.404, 95%CI: 1.082–1.917; P=0.033.	Compared to female, male is a significant risk factor for BM and OS in LD-SCLC.	
	491	Wu, 2017 (15)	BM: Competing risk regression; OS: Cox proportional hazard regression	male vs female: P>0.05	male vs female:: HR= 1.24; 95%CI: 0.92–1.67; P= 0.16	Sex is not a significant risk factor for BM or OS in LD-SCLC	No details on BM results, i.e. HR, 95%CI, and detailed P value.
	519	Zheng, 2018 (9)	Cox proportional hazard regression.	P=0.293	P=0.150	Sex is not a significant risk factor for BM or OS in LD-SCLC	Investigated multiple factors (N=21) with limited sample size (n=153).
	86	Chu, 2019 (17)	Pre-PCI BM: binary logistic regression; OS: Cox proportional hazard regression.	male vs female: OR=0.510, 95%CI: 0.107–2.437, P=0.399.	male vs female: HR=1.725, 95%CI: 0.728–4.086, P=0.215	Sex is not a significant risk factor for pre-PCI BM or OS in LD-SCLC	13.6% (15/110) patients were female; Investigated risk factors for Pre-PCI BM in LD-SCLC using logistic regression.
2) ED-SCLC: 80 has available data for meta-analysis							
	80	Chen, 2016 (13)	Cox proportional hazard regression	HR, 1.254; 95%CI: 0.774–2.033; p>0.05;	HR, 0.991; 95%CI: 0.603–1.628; p>0.05;	Sex is not a significant risk factor for BM or OS in ED-SCLC	
	81	Chen, 2018 (8)	BM: Logistic regression. OS: Cox proportional hazard regression	Female *vs* male: (adjust for age, PS, tumor load, number of metastatic sites, PCI timing): OR=0.616, 95%CI: 0.200–1.896; P >0.05.	Female *vs* male: HR=0.976, 95%CI: 0.314–1.368; P=0.945.	Sex is not a significant risk factor for BM or OS in ED-SCLC	Logistic regression was used for BM analysis.
	28	Bang, 2018 (16)	Cox proportional hazard regression	P>0.05	P>0.05	Sex is not a significant risk factor for BM or OS in ED-SCLC	Backward stepwise multivariate analysis
3) SCLC: 377, 514, 439 have available data for meta-analysis							
	376	Sahmoun, 2004 (3)	Cox proportional hazard regression.	male vs female (adjust for hypertension, age, BMI, laterality): HR=1.01, 95%CI: 0.6-1.6; P: NI.	NI	Sex is not a significant risk factor for BM in SCLC without PCI.	Investigated only demographic factors, did not consider tumor and treatment related factors Data overlapped with No.377.
	377	Sahmoun, 2005 (12)	Cox proportional-hazards regression models	male vs female (adjust for treatment, stage, BMI, age, laterality, anatomical site, PCI): HR=1.11, 95%CI: 0.67-1.83; P: NI.	male vs female (adjust for treatment, stage, BMI, age, laterality, anatomical site): HR=0.55, 95%CI: 0.34-0.88; P: NI.	Compared to female, male is an independent risk factor for OS, but not for BM in SCLC.	The hazards model of OS did not include PCI. Observed events were different in table II and table III.
	514	Zeng, 2017 (7)	Cox proportional hazard regression.	HR=1.12, 95%CI: 0.53-2.36; P=0.760	NI	Sex is not a significant risk factor for BM after PCI in SCLC	
	439	Suzuki, 2018 (5)	Cox proportional hazard regression.	male vs female: HR: 1.109, 95%CI: 0.766–1.604; P= 0.584.	NI	Sex is not a significant risk factor for BM in SCLC	
	203	Kim, 2019 (6)	Cox proportional hazard regression.	male vs female: HR: 0.500, 95%CI: 0.270–0.368, P=0.027; adjust for age, T, and PCI: P=0.167.	P>0.05	Male is a risk factor for BM in LD-SCLC, but not for OS.	No HR in the 95%CI. Inverse probability treatment weight (IPTW) was used to minimize bias; No report of patients distribution in each group after IPTW; Details of multivariate model not reported.
	513	Zeng, 2019 (10)	Competing-risk regression	HR=1.01, 95%CI: 0.69-1.48; P= 0.94;	NI	Sex is not a significant risk factor for BM after PCI in SCLC	
4. Smoking: 2 studies (519, 514) have qualified BM data to perform Meta-analysis, no qualified data for OS meta-analysis							
	520	Zhu, 2014 (4)	Cox proportional hazard regression.	Yes vs No: P= 0.559	P= 0.594	Smoking is not a significant risk factor for BM or OS in resected LD-SCLC	
	514	Zeng, 2017 (7)	Cox proportional hazard regression.	Yes vs No: HR=0.82, 95%CI: 0.41–1.63; P=0.572	NI	Smoking is not a significant risk factor for BM after PCI in SCLC	
	519	Zheng, 2018 (9)	Cox proportional hazard regression.	No *vs* Yes (adjust for NLR, blood glucose, NSE, T, TRT timing, chemo cycles): HR=1.47, 95%CI: 0.78–2.75; P =0.235.	P=0.277	Smoking is not a significant risk factor for BM in LD-SCLC	Investigated multiple factors (N=21) with limited sample size (n=153).
	439	Suzuki, 2018 (5)	Cox proportional hazard regression.	Current smoking vs no: HR: 1.218, 95%CI: 0.831–1.786; P= 0.312.	NI	Current smoking is not a significant risk factor for BM in SCLC	No data for ever smoking or not.
	28	Bang, 2018 (16)	Cox proportional hazard regression	Smoking during chemo vs no: P>0.05	Smoking during chemo vs no: P>0.05	Smoking during chemo is not a significant risk factor for BM or OS in ED-SCLC	Backward stepwise multivariate analysis
	513	Zeng, 2019 (10)	Competing-risk regression	Yes vs No: HR: 0.98, 95%CI: 0.69–1.39; P= 0.93.	NI	Smoking is not a significant risk factor for BM after PCI in SCLC	
	86	Chu, 2019 (17)	Pre-PCI BM: binary logistic regression; OS: Cox proportional hazard regression.	Yes vs no (adjust for CRT-D, T, and N): OR=4.376, 95%CI: 0.895–21.394, P=0.068	Yes vs no: HR=1.205, 95%CI: 0.614–2.366, P=0.588	Smoking is not a significant risk factor for pre-PCI BM or OS in LD-SCLC	Investigated risk factors for Pre-PCI BM in LD-SCLC using logistic regression.
5. BMI: 2 studies (377, 376) have overlapped BM data for meta-analysis. Therefore, meta-analysis was not performed to avoid bias.							
	376	Sahmoun, 2004 (3)	Cox proportional hazard regression.	<25 vs ≥ 25 kg/m^2^ (adjust for hypertension, age, sex, laterality): HR=1.01, 95%CI: 0.6-1.6; P: NI.	NI	BMI is not a significant risk factor for BM in SCLC without PCI.	Investigated only demographic factors, did not consider tumor and treatment related factors Data overlapped with 377.
	377	Sahmoun, 2005 (12)	Cox proportional-hazards regression	<25 vs ≥ 25 kg/m^2^ (adjust for treatment, stage, age, sex, laterality, anatomical site, PCI): HR=0.94, 95%CI: 0.57-1.54; P: NI.	<25 vs ≥ 25 kg/m^2^ (adjust for treatment, stage, age, sex, laterality, anatomical site): HR=1.85, 95%CI: 1.25-2.86; P: NI.	Compared to normal weight, overweight is an independent risk factor for OS, but not for BM.	The hazards model of OS did not include PCI.
	519	Zheng, 2018 (9)	Cox proportional hazard regression.	<25 vs ≥ 25 kg/m^2^: P=0.075	P=0.404	BMI is not a significant risk factor for BM or OS in LD-SCLC	Investigated multiple factors (N=21) with limited sample size (n=153).
6. Weight loss: No qualified data to perform meta-analysis (different statistical analysis).							
	239^C^	Levy, 2019 (19) (CONVERT trial)	BM: Competing risk regression; OS: Cox proportional hazard regression	≤ 10% vs > 10% (adjust by Log (tGTV), ODRT/TDRT, Brain MRI/CT, PS, PCI timing, PCI dose): HR: 1.83; 95% CI: 0. 69–4.89; P=0.230	≤ 10% vs > 10% (adjust by Log (tGTV), TDRT vs ODRT, Brain MRI/CT, PS, PCI timing, PCI dose): HR: 1.98; 95% CI: 0.14–3.43; P=0.015	Weight loss >10% is an independent risk factor for OS in LD-SCLC with PCI, but not for BM.	Data from RCT
	145	Greenspoon, 2011 (20)	logistic regression	≥ 5 kg vs <5kg (adjust for chemo response): OR=0.69, 95%CI: 0.49-0.97; P= 0.03	NI	Weight loss more than 5kg was an independent risk factor for BM in ED-SCLC.	Logistic regression was used for BM analysis . BM time definition and follow-up period were not reported. No report of patients distribution in each group.
7. Chronic disease	519	Zheng, 2018 (9)	Cox proportional hazard regression.	Yes vs No: P=0.056	P=0.879	Chronic disease is not a significant risk factor for BM or OS in LD-SCLC.	Investigated multiple factors (N=21) with limited sample size (n=153).
8. Hypertension	376	Sahmoun, 2004 (3)	Cox proportional hazard regression.	No vs Yes (adjust for, age, sex, laterality, BMI): HR=1.11, 95%CI: 0.7-1.8; P: NI.	NI	Hypertension is not a significant risk factor for BM in SCLC without PCI.	Investigated only demographic factors, did not consider tumor and treatment related factors
***B. Tumor related factors***							
1. Histology (SCLC vs combined SCLC): Meta-analysis for BM is not applicable because of different statistics and no HR data							
	139	Gong, 2013 (11)	Cox proportional hazard regression.	(Adjust for surgical resection, stage, induction chemo, adjuvant chemo, and PORT): HR=2.002, 95%CI: NI; P=0.099.	NI	Combined SCLC is not a significant risk factor for BM in resected LD-SCLC.	Contained many patients with combined SCLC and NSCLC (53.5%, 69/129). The impact of histology on OS was not analyzed.
	491	Wu, 2017 (15)	BM: Competing risk regression; OS: Cox proportional hazard regression	P>0.05	HR= 1.15; 95%CI: 0.60–2.20; P= 0.67.	Combined SCLC is not a significant risk factor for BM or OS in LD-SCLC	Only 6% (17/283) patients were with combined SCLC and NSCLC; No details on BM results, i.e. HR, 95%CI, and detailed P value.
2. Tumor size: Meta-analysis for BM is not applicable because of different analysis methods							
	239^C^	Levy, 2019 (19) (CONVERT trial)	BM: Competing risk regression; OS: Cox proportional hazard regression	Log (tGTV) (adjust by ODRT/TDRT, brain CT/MRI, weight loss, PS, PCI timing, PCI dose): HR: 1.43; 95% CI: 1.11–1.85; P=0.006	Log (tGTV) (adjust by ODRT/TDRT, brain CT/MRI, weight loss, PS, PCI timing, PCI dose): HR: 1.33; 95% CI: 1. 16–1.54; P<0.001	tGTV is an independent risk factor for BM and OS in LD-SCLC with PCI	Data from RCT.
	115	Farooqi, 2017 (1)	BM: Competing-risk regression. OS: Cox proportional hazard regression	<5 *vs* ≥5 cm: HR 1.77, 95% CI 1.22–2.55, P=0.002; SHR 1.66, 95% CI 1.15–2.40, P=0.007; Multivariate (adjusted factors: NI): P>0.05	HR 1.16, 95% CI 0.96–1.40, P=0.114	Tumor size is not an independent risk factor for BM or OS in LD-SCLC	Two definitions for time to development of BM, unclear which one is used
	519	Zheng, 2018 (9)	Cox proportional hazard regression.	<5 *vs* ≥5 cm: P=0.065	P=0.764	Tumor size is not a significant risk factor for BM or OS in LD-SCLC	Investigated multiple factors (N=21) with limited sample size (n=153).
	203	Kim, 2019 (6)	Cox proportional hazard regression.	<50 *vs* ≥50 ml: HR=0.909, 95%CI: 0.413–2.000, P=0.812.	P>0.05	Tumor volume is not a significant risk factor for BM or OS in LD-SCLC.	Inverse probability treatment weight (IPTW) was used to minimize bias; No report of patients distribution in each group after IPTW; Details of multivariate model not reported.
3. T stage: 3 studies (519, 34, 203) have qualified BM data for meta-analysis, no qualified data for OS meta-analysis							
	34	Bernhardt, 2017 (2)	Cox proportional hazard regression	1-2 vs 3-4: HR 0.76, 95% CI 0.39-1.46, P= 0.41;	HR 1.10, 95% CI 0.72-1.69, P= 0.64;	T is not a significant risk factor for BM or OS in ED-SCLC with PCI	No report of patients distribution in each group
	519	Zheng, 2018 (9)	Cox proportional hazard regression.	1-2 vs 3-4 (adjust for smoking, blood glucose, NSE, NLR, TRT timing, chemo cycles): HR=2.27, 95%CI:1.11–4.61, P= 0.024;	P=0.614	T stage is an independent risk factor for BM in LD-SCLC, but not for OS	Investigated multiple factors (N=21) with limited sample size (n=153).
	86	Chu, 2019 (17)	Pre-PCI BM: Logistic regression; OS: Cox proportional hazard regression.	1-2 vs 3-4 (adjust for smoking, CRT-D, and N): OR=1.099, 95%CI: 0.411–2.941, P=0.851	T1-2 vs T3-4 (adjust for CRT-D and N): HR=2.610, 95%CI: 1.364–4.993, P=0.004	T is an independent risk factor for OS in LD-SCLC, but not for pre-PCI BM.	Investigated risk factors for Pre-PCI BM in LD-SCLC using logistic regression.
	203	Kim, 2019 (6)	Cox proportional hazard regression.	0-2 vs 3-4: HR=1.787, 95%CI: 0.894–3.573, P=0.101; adjust for age, sex, and PCI: P=0.253.	P>0.05	T is not a significant risk factor for BM or OS in LD-SCLC	male vs female: HR: 0.500, 95%CI: 0.270–0.368, P=0.027; adjust for age, T, and PCI: P=0.167
4. N stage: Meta-analysis for BM is not applicable because of different statistics and no HR data							
	519	Zheng, 2018 (9)	Cox proportional hazard regression.	N0-1 vs N2-3: p=0.542	P=0.419	N stage is not a significant risk factor for BM or OS in LD-SCLC	Investigated multiple factors (N=21) with limited sample size (n=153).
	203	Kim, 2019 (6)	Cox proportional hazard regression.	0-1 vs 2-3: HR=1.452, 95%CI: 0.731–2.884, P=0.286.	Adjust for PS, LDH, stage, TRT dose, TRT timing, PCI: P>0.05	N is not a significant risk factor for BM or OS in LD-SCLC.	Inverse probability treatment weight (IPTW) was used to minimize bias; No report of patients distribution in each group after IPTW; Details of multivariate model not reported.
	86	Chu, 2019 (17)	Pre-PCI BM: Logistic regression; OS: Cox proportional hazard regression.	N0-2 vs N3 (adjust for smoking, CRT-D, and T): OR=1.389, 95%CI: 0.456–4.235, P=0.564	N0-2 vs N3 (adjust for CRT-D and T): HR=2.160, 95%CI: 1.056–4.417, P=0.035	N is an independent risk factor for OS in LD-SCLC, but not for pre-PCI BM.	Investigated risk factors for Pre-PCI BM in LD-SCLC using logistic regression.
5. c-stage							
1) I-II vs III: Meta-analysis for BM is not applicable because of different statistics and no HR data							
	491	Wu, 2017 (15)	BM: Competing risk regression; OS: Cox proportional hazard regression	I-II vs III (adjust for PCI, chemo): HR, 2.09; 95% CI, 1.08–4.04; P = 0.028.	I-II vs III (adjust for PCI, chemo): HR, 1.97; 95% CI, 1.38–2.80; P <0.001.	Compared to stage 1-II, stage III is an independent risk factor for BM and OS in LD-SCLC.	
	519	Zheng, 2018 (9)	Cox proportional hazard regression.	I-II vs III: p= 0.093	P=0.503	cTNM stage is not a significant risk factor for BM or OS in LD-SCLC	Investigated multiple factors (N=21) with limited sample size (n=153).
	203	Kim, 2019 (6)	Cox proportional hazard regression.	I-II vs III : HR=1.305, 95%CI: 0.660–2.580, P=0.444.	Adjust for PS, N, LDH, TRT dose, TRT timing, PCI: P>0.05.	Stage is not a significant risk factor for BM or OS in LD-SCLC.	Inverse probability treatment weight (IPTW) was used to minimize bias; No report of patients distribution in each group after IPTW; Details of multivariate model not reported.
	303	Nakamura, 2018 (21)	BM: χ^2^-test; OS: Cox proportional hazard regression	BM as a first recurrence site: Stage II: 22% (5/23); Stage III: 29% (40/139); P=0.485	III vs II (adjust for age, ODRT/TDRT, pulmonary effusion, PCI, SER): HR=0.51, 95%CI: 0.27–0.94, P=0.031.	Stage was an independent risk factor for OS in LD-SCLC, but not for BM	χ^2^-test was used for BM analysis; No overall BM results
2) ≤IIIA vs ≥IIIB: Meta-analysis for BM is not applicable because of overlapped data							
	122	Fu, 2014 (14)	Cox proportional-hazard regression	BM as a first recurrence site: IIIA vs IIIB (adjust for age, sex, PS, CTC at baseline, CTC post-first cycle, CTC post-fourth cycle, response): HR=1.601, 95%CI: 0.762–3.366; P=0.214.	NI	Stage is not a significant risk factor for BM after PCI in stage III SCLC	Analyzed BM as a first site of recurrence; No report of patients distribution in each group; Data overlapped with 514.
	514	Zeng, 2017 (7)	Cox proportional hazard regression.	I-IIIA vs IIIB-IV (adjust for sex, age, smoking, response, TDRT/ODRT, CCRT/SCRT, chemo cycles, brain CT/MRI): HR = 2.119, 95%CI 0.932–4.821, p = 0.073.	HR = 2.002, 95% CI 1.180–3.395, p = 0.010	Compared to stage I-IIIA, stage IIIB-IV was a significant risk factor for OS and tended to be an independent risk factor for BM after PCI in SCLC.	
3) I-III vs IV	439	Suzuki, 2018 (5)	Cox proportional hazard regression.	I-III vs IV (adjust for PS, number of extrathoracic metastatic sites, TRT dose, PCI, pretreatment LDH, Pretreatment PLR): HR: 1.062, 95% CI: 0.618–1.826, P=0.826	NI	Stage is not a significant risk factor BM in SCLC	
4) LD vs ED: 2 studies (377, 514) have qualified BM and OS data for meta-analysis							
	397	Seute, 2004 (22)	Log- rank test	2-year BM: LD: 49%, ED: 65%; P: NI	Median OS: 8.5 months (range, 0–154 months): ED (n=284): 7.2 months (range, 0–124 months), LD (n=137): 11.9 months (range, 0–154 months) (P<0.0005).	ED is a risk factor for BM and OS in SCLC,	No HR or P value for BM.
	377	Sahmoun, 2005 (12)	Cox proportional-hazards regression models	LD vs ED (adjust for treatment, BMI, age, sex, laterality, anatomical site, PCI): HR=4.63, 95%CI:1.80-11.9; P: NI	LD vs ED (adjust for treatment, BMI, age, sex, laterality, anatomical site, PCI): HR=2.24, 95%CI: 1.17-4.3; P: NI.	Compared to LD, ED is an independent risk factor for BM and OS.	The hazards model of OS did not include PCI.
	356	Ramlov, 2012 (23)	Log- rank test	BM prevalence: 21/118 (17.8%): LD: 14/74 (18.9%); ED: 7/44 (15.9) (p>0.05).	Median OS: 16.0 months (95%CI 13.0–19.0): LD: 24.0 months (19.6–28.3), ED: 12.0 months (9.6–14.4) (p < 0.001).	ED is a risk factor for OS in SCLC with PCI, but not for BM.	No HR reported.
	514	Zeng, 2017 (7)	Cox proportional hazard regression.	LD vs ED (adjust for sex, age, smoking, response, TDRT/ODRT, CCRT/SCRT, chemotherapy cycles, brain CT/MRI): HR=1.76y, 95%CI: 0.63-4.92; P=0.280.	HR=1.141, 95% CI 0.543-2.395,P= 0.728	LD/ED is not a significant risk factor for BM or OS in SCLC with PCI.	
	513	Zeng, 2019 (10)	BM: Competing-risk regression; OS: Cox proportional-hazards regression models	LD vs ED (adjust for era, PS, CCRT/SCRT, ODRT/TDRT, timing of PCI): HR=1.69, 95%CI:1.03-2.77, P=0.04	LD vs ED (adjust for era, PS, CCRT/SCRT, ODRT/TDRT, timing of PCI): HR=1.27, 95%CI: 0.90-1.79, P=0.17.	ED is an independent risk factor for BM after PCI in SCLC, but not for OS.	
6. p-stage: I,II,III: Meta-analysis for BM is not applicable because of different statistical analysis.							
	139	Gong, 2013 (11)	Cox proportional hazard regression.	(Adjust for surgical resection, histology, induction chemo, adjuvant chemo, and PORT): HR=2.458, 95%CI: NI; P=0.002.	(Adjust for surgical resection, BM, induction chemo, adjuvant chemo, and PORT): HR=2.391, 95%CI: NI; P=0.001.	Stage is an independent risk factor for BM and OS in resected LD-SCLC.	Contained many patients with combined SCLC and NSCLC (53.5%, 69/129); The factors in multivariate model of BM and OS were different.
	520	Zhu, 2014 (4)	Cox proportional hazard regression.	(Adjust for LVI and PORT): HR = 2.013, 95%CI: 1.135 ~ 3.569; p = 0.017.	(adjust for age, PS, LVI, and BM): HR=2.093, 95%CI: 1.399- 3.132; P=0.001.	Stage is an independent risk factor for BM and OS in resected LD-SCLC.	BM was included in the multivariate model of OS.
7. LVI	520	Zhu, 2014 (4)	Cox proportional hazard regression.	Yes vs no (adjust for p-stage and PORT): HR = 1.924, 95%CI: 1.002 ~ 3.291; p = 0.039.	(adjust for age, PS, stage, and BM): HR=0.935, 95%CI: 0.507- 1.723; P=0.829.	LVI is an independent risk factor for BM in resected LD-SCLC, but not for OS.	BM was included in the multivariate model of OS.
8. M status in ED-SCLC: 3 studies (80, 34, 28) have qualified BM and OS data for meta-analysis							
	80	Chen, 2016 (13)	Cox proportional hazard regression	Distant metastases vs. locally advanced: HR, 1.234; 95%CI: 0.826–1.843; p>0.05;	HR, 1.410; 95%CI: 0.959–2.084; p>0.05;	Distant metastases is not a significant risk factor for BM or OS in ED-SCLC	
	34	Bernhardt, 2017 (2)	Cox proportional hazard regression	M1b or not: HR 0.69, 95% CI 0.27-1.78, P= 0.44;	M1b or not: HR 1.25, 95% CI 0.63-2.48, P= 0.51;	M1b is not a significant risk factor for BM or OS in ED-SCLC with PCI	No report of patients distribution in each group
	28	Bang, 2018 (16)	Cox proportional hazard regression	Extrathoracic metastases (No vs Yes) (adjust for PCI): HR 2.59; 95% CI: 1.12-7.56; P=0.02;	Extrathoracic metastases (No vs Yes) (adjust for PS, PCI): HR 1.75; 95% CI:1.04-3.17; P = 0.03	Extrathoracic metastases is an independent risk factor for BM and OS in ED-SCLC.	Backward stepwise multivariate analysis
	81	Chen, 2018 (8)	BM: Logistic regression. OS: Cox proportional hazard regression	Distant metastases vs. locally advanced (adjust for age, sex, PS, number of metastatic sites, PCI timing): OR=2.944, 95%CI: 1.049–8.261; P >0.05.	Distant metastases vs. locally advanced: HR=2.018, 95%CI: 1.159–3.517; P =0.013.	Distant metastases is a significant risk factor for OS in ED-SCLC, but not for BM.	Logistic regression was used for BM analysis.
9. Number of metastatic sites: Meta-analysis for BM is not applicable because of different statistical analysis							
	80	Chen, 2016 (13)	Cox proportional hazard regression	≥2 *vs* <2: HR, 1.124; 95% CI, 0.688–1.835; p> 0.05;	≥2 *vs* <2: (adjust for PCI, liver metastasis, PS): HR, 1.146; 95%CI: 0.722–1.820; p>0.05.	Number of metastatic sites is not a significant risk factor for BM or OS in ED-SCLC.	
	81	Chen, 2018 (8)	BM: Logistic regression. OS: Cox proportional hazard regression	≥2 *vs* <2 (adjust for age, sex, PS, tumor load, PCI timing): OR=1.445, 95%CI: 0.284–7.354; P >0.05.	≥2 *vs* <2: HR=1.758, 95%CI: 0.697–4.435; P=0.232.	Number of metastatic sites is not a significant risk factor for BM or OS in ED-SCLC.	Logistic regression was used for BM analysis.
10. Number of extrathoracic metastatic sites	439	Suzuki, 2018 (5)	Cox proportional hazard regression.	≤ 4 vs > 4 (adjust for PS, stage, TRT dose, PCI, pretreatment LDH, Pretreatment PLR): HR: 0.978, 95% CI: 0.620–1.543, P=0.924.	NI	Number of extrathoracic metastatic sites is not a significant risk factor BM in SCLC.	
11. Metastatic organs							
1) Bone metastasis: Meta-analysis for BM is not applicable because of different statistical analysis.							
	145	Greenspoon, 2011 (20)	logistic regression	Yes vs No: OR=0.68, 95%CI: 0.24-1.94; P= 0.47.	NI	Bone metastasis is not a significant risk factor for BM in ED-SCLC.	Logistic regression was used for BM analysis . BM time definition and follow-up period were not reported. No report of patients distribution in each group.
	80	Chen, 2016 (13)	Cox proportional hazard regression	Yes vs no: HR, 1.234; 95%CI: 0.826–1.843; p>0.05;	HR, 1.083; 95%CI: 0.692–1.694; p>0.05;	Bone metastases is not a significant risk factor for BM or OS in ED-SCLC.	
2) Liver metastasis: Meta-analysis for BM is not applicable because of different statistical analysis.							
	145	Greenspoon, 2011 (20)	logistic regression	Yes vs No: OR=0.80, 95%CI: 0.27-2.34; P= 0.68.	NI	Liver metastasis is not a significant risk factor for BM in ED-SCLC.	Logistic regression was used for BM analysis . BM time definition and follow-up period were not reported. No report of patients distribution in each group.
	80	Chen, 2016 (13)	Cox proportional hazard regression	Yes *vs* no (adjust for PCI, Number of metastatic sites): HR, 2.511; 95%CI: 1.408–4.477; p<0.05;	Yes *vs* no (adjust for PCI, Number of metastatic sites, PS): HR, 2.193; 95%CI: 1.284–3.747; p<0.05;	Liver metastasis is an independent risk factor for BM and OS in ED-SCLC	
3) Adrenal metastasis: Meta-analysis for BM is not applicable because of different statistical analysis.							
	145	Greenspoon, 2011 (20)	logistic regression	Yes vs No: OR=0.84, 95%CI 0.22-3.24; P= 0.80.	NI	Adrenal metastasis is not a significant risk factor for BM in ED-SCLC.	Logistic regression was used for BM analysis . BM time definition and follow-up period were not reported. No report of patients distribution in each group.
	80	Chen, 2016 (13)	Cox proportional hazard regression	Yes vs no: HR, 1.778; 95%CI: 0.946–3.344; p>0.05;	HR, 1.396; 95%CI: 0.725–2.687; p>0.05;	Adrenal metastases is not a significant risk factor for BM or OS in ED-SCLC.	
4) Lung metastasis	80	Chen, 2016 (13)	Cox proportional hazard regression	Yes vs no: HR, 0.886; 95%CI: 0.526–1.493; p>0.05;	HR, 0.828; 95%CI: 0.499–1.374; p>0.05;	Lung metastases is not a significant risk factor for BM or OS in ED-SCLC.	
12. Laterality: Meta-analysis for BM is not applicable because of different analysis and overlapped data.							
	376	Sahmoun, 2004 (3)	Cox proportional hazard regression.	Left vs right (adjust for hypertension, age, sex, BMI): HR=1.11, 95%CI: 0.7-1.8; P: NI.	NI	Laterality is not a significant risk factor for BM in SCLC without PCI.	Investigated only demographic factors, did not consider tumor and treatment related factors Data overlapped with 377.
	377	Sahmoun, 2005 (12)	Cox proportional-hazards regression	Left vs right (adjust for treatment, stage, BMI, age, sex, anatomical site, PCI): HR=1.25, 95%CI: 0.84-1.89; P: NI.	Left vs right (adjust for treatment, stage, BMI, age, sex, anatomical site): HR=1.52, 95%CI: 1.01-2.3; P: NI.	Compared to left , right SCLC is an independent risk factor for OS, but not for BM.	The hazards model of OS did not include PCI.
	513	Zeng, 2019 (10)	Competing-risk regression	left vs right: HR=0.94, 95%CI: 0.67-1.32; P=0.71.	NI	Laterality is not a significant risk factor for BM after PCI in SCLC	
13. Anatomical site	377	Sahmoun, 2005 (12)	Cox proportional-hazards regression models	lower vs upper lobe (adjust for treatment, stage, BMI, age, sex, laterality, PCI): HR=0.70, 95%CI: 0.42-1.16; P: NI.	lower vs upper lobe (adjust for treatment, stage, BMI, age, sex, laterality): HR=0.90, 95%CI: 0.54-1.53; P: NI.	Anatomical site is not a significant risk factor for BM or OS in LD-SCLC	The hazards model of OS did not include PCI.
14. KPS^D^: Meta-analysis for BM is not applicable because of different analysis methods.							
	520	Zhu, 2014 (4)	Cox proportional hazard regression.	≥80 *vs* <80: P= 0.272	(adjust for age, stage, LVI, and BM): HR=1.149, 95%CI: 0.631-2.092; P=0.649.	KPS is not a significant risk factor for BM or OS in resected LD-SCLC	BM was included in the multivariate model of OS
	115	Farooqi, 2017 (1)	BM: Competing-risk regression. OS: Cox proportional hazard regression	≥80 *vs* <80: SHR 0.89, P=0.668;	HR 1.41, 95% CI 1.09–1.83, P=0.010; Multivariate (adjusted factors: NI): P>0.05	KPS is not an independent risk factor for BM or OS in LD-SCLC.	Two definitions for time to development of BM, unclear which one is used
	491	Wu, 2017 (15)	BM: Competing risk regression; OS: Cox proportional hazard regression	≥80 *vs* <80: P>0.05	≥80 *vs* <80: HR= 0.75; 95%CI: 0.50–1.11; P= 0.15	KPS is not a significant risk factor for BM or OS in LD-SCLC	No details on BM results, i.e. HR, 95%CI, and detailed P value.
	34	Bernhardt, 2017 (2)	Cox proportional hazard regression	≤ 70 vs > 70: HR 0.71, 95% CI 0.35-1.41, P= 0.33;	HR 0.85, 95% CI 0.55-1.33, P= 0.49;	KPS is not a significant risk factor for BM or OS in ED-SCLC with PCI	No report of patients distribution in each group
	371	Rubenstein, 1995 (24)	Multivariate Cox regression	Pre-RT KPS (≤ 80 vs > 80) (adjusted factors: PCI, response, age, treatment intent): HR: NI, P=0.04.	pre-RT KPS (≤ 80 vs > 80) (adjusted factors: PCI, response, age, CCRT/SCRT): HR: NI, P = 0.0001	Pre-RT KPS was a significant risk factor for BM and OS in LD-SCLC	Did not report HR;
15. PS^D^							
1) 0-1 vs ≥ 2: 2 studies (80, 439) have qualified BM data for meta-analysis, no qualified data for OS meta-analysis.							
	80	Chen, 2016 (13)	Cox proportional hazard regression	0-1 vs 2: HR, 2.383; 95% CI, 0.866–6.560; p> 0.05;	0-1 vs 2: (adjust for PCI, liver metastasis, number of metastatic sites) : HR, 3.182; 95%CI: 1.534–6.599; p<0.05;	PS is an independent risk factor for OS in ED-SCLC, but not for BM.	
	81	Chen, 2018 (8)	BM: Logistic regression. OS: Cox proportional hazard regression	0-1 vs 2: (adjust for age, sex, tumor load, number of metastatic sites, PCI timing): OR=6.001, 95%CI: 0.509–70.727; P >0.05.	0-1 vs 2: (adjust for age, sex, tumor load, number of metastatic sites, PCI timing): HR=2.545, 95%CI: 0.788–8.217; P=0.118.	PS is not a significant risk factor for BM or OS in ED-SCLC	Logistic regression was used for BM analysis.
	439	Suzuki, 2018 (5)	Cox proportional hazard regression.	0-1 vs ≥ 2 (adjust for stage, number of extrathoracic metastatic sites, TRT dose, PCI, pretreatment LDH, Pretreatment PLR): HR: 1.369, 95% CI: 0.834–2.246, P=0.214.	NI	PS is not a significant risk factor BM in SCLC	
	28	Bang, 2018 (16)	Cox proportional hazard regression	0-1 vs 2-4: P>0.05	0-1 vs 2-4 (adjust for PS, PCI, Extrathoracic metastases): HR 1.75; 95% CI:1.04-3.17; P = 0.03	PS is an independent risk factor for OS in ED-SCLC, but not for BM.	Backward stepwise multivariate analysis
2) 0 vs 1-2: Meta-analysis for BM is not applicable because of different analysis methods and no HR data.							
	239^C^	Levy, 2019 (19) (CONVERT trial)	BM: Competing risk regression; OS: Cox proportional hazard regression	0 vs 1-2 (adjust by Log (tGTV), ODRT/TDRT, Brain MRI/CT, Weight loss, PCI timing, PCI dose): HR: 0.54; 95% CI: 0.32–0.90; P=0.018	0 vs 1-2 (adjust by Log (tGTV), TDRT vs ODRT, Brain MRI/CT, Weight loss, PCI timing, PCI dose): HR: 1.1; 95% CI: 0.86–1.46; P=0.348	Better PS is an independent risk factor for BM after PCI in LD-SCLC, but not for OS.	Data from RCT,
	519	Zheng, 2018 (9)	Cox proportional hazard regression.	0 vs 1-2: P= 0.455	P=0.805	PS is not a significant risk factor for BM in LD-SCLC	Investigated multiple factors (N=21) with limited sample size (n=153).
	203	Kim, 2019 (6)	Cox proportional hazard regression.	0 vs 1-2: HR=1.788, 95%CI: 0.554–5.773, P=0.331.	Adjust for LDH, N, stage, TRT dose, TRT timing, PCI: P>0.05.	PS is not a significant risk factor for BM or OS in LD-SCLC.	Inverse probability treatment weight (IPTW) was used to minimize bias; No report of patients distribution in each group after IPTW; Details of multivariate model not reported.
3) Others: Meta-analysis for BM is not applicable because of different analysis methods.							
	513	Zeng, 2019 (10)	BM: Competing risk regression; OS: Cox proportional hazard regression	0,1,2 (adjust for era, stage, ODRT/TDRT, SCRT/CCRT, PCI timing): HR=1.25, 95%CI: 0.81–1.91, P=0.32.	0,1,2 (adjust for era, stage, ODRT/TDRT, SCRT/CCRT, PCI timing): HR=1.38, 95%CI: 1.03–1.83, P=0.03.	PS is an independent risk factor for OS in SCLC with PCI, but not for BM.	
	122	Fu, 2014 (14)	Cox proportional-hazard regression	BM as a first recurrence site: 0-3 vs >3 (adjust for age, sex, stage, CTC at baseline, CTC post-first cycle, CTC post-fourth cycle, response): HR= 0.397, 95%CI: 0.046–3.432; P=0.401.	NI	PS is not a significant risk factor for BM after PCI in stage III SCLC	Analyzed BM as a first site of recurrence; No report of patients distribution in each group.
	145	Greenspoon, 2011 (20)	logistic regression	0-2 vs 3-4: OR=0.39, 95%CI: 0.08-1.86; P= 0.24.	NI	PS is not a significant risk factor for BM in ED-SCLC.	Logistic regression was used for BM analysis. BM time definition and follow-up period were not reported. No report of patients distribution in each group.
16. Response^E^: Meta-analysis for BM is not applicable because of different analysis methods and no HR data.							
	371	Rubenstein, 1995 (24)	Multivariate Cox regression	Response to induction chemo (CR/Near CR vs others) (adjusted factors: PCI, KPS, age, treatment intent) HR: NI, P>0.05.	Response to induction chemo (CR/Near CR vs others) (adjusted factors: PCI, Pre-RT KPS, age, CCRT/SCRT): HR: NI, P = 0.0173	Response was a significant risk factor for OS in LD-SCLC, but not for BM.	NoHR given; Did not report compared response in detail.
	519	Zheng, 2018 (9)	Cox proportional hazard regression.	PR vs CR: P= 0.308	P=0.102	Response is not a significant risk factor for BM in LD-SCLC	Investigated multiple factors (N=21) with limited sample size (n=153).
	28	Bang, 2018 (16)	Cox proportional hazard regression	PR vs CR: P>0.05	PR vs CR: P>0.05	Response is not a significant risk factor for BM or OS in ED-SCLC	Backward stepwise multivariate analysis
	514	Zeng, 2017 (7)	Cox proportional hazard regression.	PR/SD vs CR: P=0.842	NI	Response is not a significant risk factor for BM after PCI in SCLC	
	122	Fu, 2014 (14)	Cox proportional-hazard regression	(adjust for age, sex, PS, CTC at baseline, CTC post-first cycle, CTC post-fourth cycle, stage): HR= 1.727, 95%CI: 0.718–4.152; P=0.222.	NI	Response is not a significant risk factor for BM after PCI in stage III SCLC	Analyzed BM as a first site of recurrence; No report of patients distribution in each group; Data overlapped with No. 514.
	145	Greenspoon, 2011 (20)	Logistic regression	Chemo response (adjust for weight loss): OR=5.49, 95%CI: 1.08-27.91; P= 0.03	NI	Chemo response was an independent risk factor for BM in ED-SCLC.	Logistic regression was used for BM analysis. BM time definition and follow-up period were not reported. No report of patients distribution in each group.
	264	Manapov, 2012 (25)	Log-rank test	BMFS: CR: 567 days, PR: 298 days, NR (SD/PD): 252 days; p <0.0001.	NI	Response significantly affects BMFS in LD-SCLC with poor initial PS	No HR given.
17. Pretreatment LDH (lactate dehydrogenase): Meta-analysis for BM is not applicable because of different cut-off values							
	439	Suzuki, 2018 (5)	Cox proportional hazard regression.	≤543 IU/L vs > 543IU/L (adjust for PS, stage, number of extrathoracic metastatic sites, TRT dose, PCI, pretreatment platelet count): HR: 1.373, 95% CI: 0.922–2.046, P =0.119.	NI	Pretreatment LDH is not a significant risk factor for BM in SCLC	
	203	Kim, 2019 (6)	Cox proportional hazard regression.	< 400 IU/L vs ≥400 IU/L: HR=1.240, 95%CI: 0.703–2.187, P=0.458.	Adjust for PS, N, stage, TRT dose, TRT timing, PCI: P>0.05	LDH is not a significant risk factor for BM or OS in LD-SCLC.	Inverse probability treatment weight (IPTW) was used to minimize bias; No report of patients distribution in each group after IPTW; Details of multivariate model not reported.
18. Neutrophil count							
1) Pretreatment	439	Suzuki, 2018 (5)	Cox proportional hazard regression.	≤3.9×10^3^/µL vs >3.9×10^3^/µL: HR: 0.807, 95%CI: 0.540–1.207; P= 0.296.	NI	Pretreatment neutrophil count is not a significant risk factor for BM in SCLC	
2) Pre-PCI	439	Suzuki, 2018 (5)	Cox proportional hazard regression.	≤3.6×10^3^/µL vs >3.6×10^3^/µL: HR: 0.764, 95%CI: 0.382−1.525; P= 0.445.	NI	Pre-PCI neutrophil count is not a significant risk factor for BM in SCLC	Cut-off value changed
19. TLC, total lymphocyte count							
1) Pretreatment	439	Suzuki, 2018 (5)	Cox proportional hazard regression.	≤1.7×10^3^/µL vs >1.7×10^3^/µL: HR: 1.024, 95%CI: 0.708–1.481; P= 0.898.	NI	Pretreatment TLC is not a significant risk factor for BM in SCLC	
2) Pre-PCI	439	Suzuki, 2018 (5)	Cox proportional hazard regression.	≤1.1×10^3^/µL vs >1.1×10^3^/µL (adjust for stage): HR: 2.512, 95%CI: 1.196–5.277; P= 0.015.	NI	Higher Pre-PCI TLC is an independent risk factor for BM in SCLC	Cut-off value changed
20. NLR, neutrophil-to-lymphocyte ratio							
1) Pretreatment: Meta-analysis for BM is not applicable because of different cut-off values							
	519	Zheng, 2018 (9)	Cox proportional hazard regression.	<2.55 vs ≥ 2.55 (adjust for smoking, blood glucose, NSE, T, TRT timing, chemo cycles): HR= 2.07, 95%CI: 1.08–3.97, P= 0.029.	<2.55 vs ≥ 2.55 (adjust for TRT timing) HR= 2.11, 95%CI:1.28-3.59; P= 0.005	Higher pretreatment NLR is an independent risk factor for BM and OS in LD-SCLC	Investigated multiple factors (N=21) with limited sample size (n=153).
	439	Suzuki, 2018 (5)	Cox proportional hazard regression.	≤1.6 vs >1.6: HR: 0.758, 95%CI: 0.433–1.326; P= 0.332.	NI	Pretreatment NLR is not a significant risk factor for BM in SCLC	
2) Pre-PCI	439	Suzuki, 2018 (5)	Cox proportional hazard regression.	≤2.3 vs >2.3: HR: 0.498, 95%CI: 0.240–1.033; P= 0.061.	NI	Pre-PCI NLR is not a significant risk factor for BM in SCLC	Cut-off value changed
21. Platelet count							
1) Pretreat-ment	439	Suzuki, 2018 (5)	Cox proportional hazard regression.	≤270×10^9^/L vs >270×10^9^/L(adjust for PS, stage, number of extrathoracic metastatic sites, TRT dose, PCI, pretreatment LDH): HR: 1.516, 95% CI: 1.024–2.245, P =0.038	NI	High pretreatment platelet count is an independent risk factor for BM in SCLC	
2) Pre-PCI	439	Suzuki, 2018 (5)	Cox proportional hazard regression.	≤247×10^9^/L vs >247×10^9^/L(adjust for stage): HR: 1.847, 95% CI: 0.927−3.681, P =0.081	NI	Pre-PCI platelet count is not a significant risk factor for BM in SCLC	
22. PLR, platelet-to-lymphocyte ratio							
1) Pretreatment: Meta-analysis for BM is not applicable because of different cut-off values							
	519	Zheng, 2018 (9)	Cox proportional hazard regression.	<125.7 vs ≥ 125.7: P= 0.477	P=0.401	Pretreatment PLR is not a significant risk factor for BM or OS in LD-SCLC	Investigated multiple factors (N=21) with limited sample size (n=153).
	439	Suzuki, 2018 (5)	Cox proportional hazard regression.	≤119.4 vs >119.4 (adjust for PS, stage, number of extrathoracic metastatic sites, TRT dose, PCI, pretreatment LDH): HR: 1.557, 95% CI: 0.939–2.582, P =0.086	NI	Pretreatment PLR is not a significant risk factor for BM in SCLC	
2) Pre-PCI	439	Suzuki, 2018 (5)	Cox proportional hazard regression.	≤69.3 vs >69.3 (adjust for stage): HR: 0.409, 95% CI: 0.173–0.969, P = 0.042	NI	Lower Pre-PCI PLR is an independent risk factor for BM in SCLC	Cut-off value changed
23. Pretreat-ment NSE	519	Zheng, 2018 (9)	Cox proportional hazard regression.	<17 vs ≥ 17 ng/ml (adjust for smoking, blood glucose, NLR, T, TRT timing, chemo cycles): HR= 3.84, 95%CI: 0.90–16.40, P= 0.069.	P=0.280	NSE is not a significant risk factor for BM or OS in LD-SCLC	Investigated multiple factors (N=21) with limited sample size (n=153).
24. Pretreat-ment CEA	519	Zheng, 2018 (9)	Cox proportional hazard regression.	<3.4 vs ≥3.4 ng/ml: P= 0.111	P=0.272	CEA is not a significant risk factor for BM or OS in LD-SCLC	Investigated multiple factors (N=21) with limited sample size (n=153).
25. Pretreat-ment blood glucose	519	Zheng, 2018 (9)	Cox proportional hazard regression.	≤6.2 vs >6.2 mmol/L (adjust for smoking, NSE, NLR, T, TRT timing, chemo cycles): HR=1.09, 95%CI: 0.50–2.41, P= 0.826.	P=0.182	Blood glucose is not a significant risk factor for BM or OS in LD-SCLC	Investigated multiple factors (N=21) with limited sample size (n=153).
26. CTC, circulating tumor cells							
1) CTC at baseline	122	Fu, 2014 (14)	Cox proportional-hazard regression	BM as a first recurrence site: (adjust for age, sex, PS, CTC post-first cycle, CTC post-fourth cycle, stage, response): HR=5.243; 95% CI, 2.133–10.574; P < 0.001. Median BM time: CTCs ≤ 218 vs CTCs > 218: 11.6 (22.3–67.7) vs 7.3 (6.8–35.2) months (p=0.001).	NI	Higher CTC at baseline is an independent risk factor for BM after PCI in stage III SCLC	Analyzed BM as a first site of recurrence; No report of patients distribution in each group
2) CTC post-first cycle	122	Fu, 2014 (14)	Cox proportional-hazard regression	BM as a first recurrence site: (adjust for age, sex, PS, CTC at baseline, CTC post-fourth cycle, stage, response): HR=1.066; 95% CI, 0.585–4.318; P =0.546.	NI	CTC post-first cycle is not a significant risk factor for BM after PCI in stage III SCLC	Analyzed BM as a first site of recurrence; No report of patients distribution in each group
3) CTC post-fourth cycle	122	Fu, 2014 (14)	Cox proportional-hazard regression	BM as a first recurrence site: (adjust for age, sex, PS, CTC post-first cycle, CTC post-fourth cycle, stage, response): HR=1.002; 95% CI, 0.776–2.371; P =0.857.	NI	CTC post-fourth cycle is not a significant risk factor for BM after PCI in stage III SCLC	Analyzed BM as a first site of recurrence; No report of patients distribution in each group
27. SUVmax	491	Wu, 2017 (15)	BM: Competing risk regression; OS: Cox proportional hazard regression	(continuous): P>0.05	(continuous): HR= 1.02; 95%CI: 0.99–1.05; P= 0.21.	SUVmax is not a significant risk factor for BM or OS in LD-SCLC	No detailed BM results reported, i.e. HR, 95%CI, and detailed P value.
***Treatment related factors***							
1. PCI vs no PCI: 3 RCTs have qualified overall BM data for meta-analysis based on Cox regression (148, 487, 19); 2 have overall BM data based on competing risk regression (415, 445); 2 have OS data (415, 445)							
1) LD-SCLC: 2 RCTs have qualified overall BM data for subgroup meta-analysis (487, 148)							
	62^C^	Cao, 2005 (26)	χ^2^-test	BM prevalence: PCI: 3.8% (1/26);No PCI: 32.0% (8/25) (χ^2^=5.15, P =0.02)	χ^2^ =2.25, P =0.13	PCI significantly decreased BM in LD-SCLC, but did not significantly improve OS	RCT; χ^2^-test was used for BM analysis
	487^C^	Work, 1996 (27)	Log-rank test	BM prevalence: PCI: 9.6%(15/157); No PCI: 31% (13/42); ( HR = 0.30, 95% CI 0.12-0.75, P =0.01);	2-year OS: PCI: 24.9%; No PCI: 16.9%; HR: NI; P=0.31	PCI significantly decreased BM in LD-SCLC, but did not significantly improve OS	RCT; Not strictly randomized;
	148^C^	Gregor, 1997 (28) (UKCCCR/EORTC)	Log-rank test	2-year BM: PCI: 30%, No PCI: 54%; HR = 0.44, 95% CI 0.29-0.67, P = 0.00004.	HR= 0.86, 95% CI 0.66-1.12, P= 0.25).	PCI significantly decreased BM in LD-SCLC, but did not significantly improve OS	RCT;
	461	van der Linden, 2001 (29)	Cox proportional hazard regression.	Overall BM: PCI: 17%; No PCI: 57%; HR: 7.3; 95% CI: 3.3 - 16.4, P<0.001	2-year OS: PCI: 42%, No PCI: 27%; HR: 1.8; 95%CI: 1.1 - 2.9, P = 0.016;	PCI significantly decreased BM and improved OS in LD-SCLC.	
	377	Sahmoun, 2005 (12)	Cox proportional-hazards regression models	No vs Yes (adjust for treatment, stage, BMI, age, sex, laterality, anatomical site): HR=0.56, 95%CI: 0.20-1.57; P: NI.	NI	PCI did not significantly decrease BM in LD-SCLC	Only 5.7% (12/209) patients received PCI.
	384	Sas-Korczyńska, 2010 (30)	BM prevalence: χ^2^-test; BMFS: Log-rank test.	PCI: 12/86 (14%), No PCI: 20/43 (46.5%); P=0.00005. 4-year BMFS: All: 67.8%, PCI: 81.8%, No PCI: 32.2% (P<0.0001).	NI	PCI significantly decreased BM in LD-SCLC	
	134	Giuliani, 2010 (31)	Cox proportional hazard regression.	HR:3.4; 95% CI: 1.9-6.1;P<0.001; multivariate (adjusted for age): HR:3.8; 95% CI: 2.1-6.8; P<0.001;	(adjusted for age) PCI: HR 2.0 (95% CI, 1.4 to 2.8; P=0.0001).	PCI significantly decreased BM and improved OS in LD-SCLC.	
	264	Manapov, 2012 (25)	Log-rank test	BM prevalence: PCI: 13.9% (5/36), No PCI: 28.1%(25/89); BMFS in patients with CR: PCI: 640 days; No PCI: 482 days; (P=0.047).	NI	PCI prolongs BMFS in LD-SCLC with poor initial PS who had CR to CRT	No HR reported.
	441	Tai, 2013 (32)	BM prevalence: χ^2^-test or Fisher exact 2-tailed test; BM time, OS: Kaplan-Meier method, Wilcoxon test.	1. Overall BM: 1) CR: PCI: 24/128 (18.8%); no PCI: 20/49 (40.8%) (Fisher P=0.002); 2) IR: PCI: 11/40 (27.5%); no PCI: 15/48 (31.3%) (Fisher P=0.70); 2. BM as first recurrence: 1) CR: PCI: 6/128 (4.7%); no PCI: 5/49 (10.2%) (Fisher P=0.18); 2) IR: PCI: 2/40 (20%); no PCI: 8/48 (16.7%) (Fisher P=0.10); 3. BM as first recurrence time: 20.7 vs. 10.6 months (*P*<0.0001)	PCI vs No PCI: 1. All: P=0.0011; 2. pts with IR: P=0.32; 3. pts with CR: P=0.15;	PCI decreases BM, improves OS	
	393	Scotti, 2014 (33)	Log-rank test.	PCI: 8/38 (21.1%); No PCI: 19/54 (35.2%); P: NI	P=0.21	BM prevalence in the PCI group was lower, but the p was not reported. PCI did not improve OS in LD-SCLC.	No P values for BM.
	115	Farooqi, 2017 (1)	BM: Competing-risk regression. OS: Cox proportional hazard regression	No PCI *vs* PCI: HR 0.54, 95% CI 0.39–0.76, P<0.001; SHR 0.56, 95% CI 0.40–0.78, P=0.001; Multivariate (adjusted factors: NI): SHR 0.57, 95% CI 0.41–0.79, p=0.001;	Multivariate (adjusted factors: NI): HR 0.76, 95% CI 0.63–0.91, p=0.003	PCI significantly improved OS and decreased BM in LD-SCLC	Two definitions for time to development of BM, unclear which one is used
	82	Choi, 2017 (34)	Cox proportional hazard regression.	cumulative first isolated BM: whole: PCI: 25.4%; No PCI: 38.9% (P = 0.014); PET: PCI: 34.3%; No PCI: 41.1% (P = 0.243); No PET: PCI: 13.3%; No PCI: 37.0% (P = 0.020).	whole: PCI: 33.1 months; No PCI: 30.7 months (P = 0.938); PET: PCI: 33.0 months; No PCI: 42.2 months (P = 0.474); No PET: PCI: 34.9 months; No PCI: 22.5 months (P = 0.569).	1. PCI decreased first isolated BM, did not improve OS in the whole group and no PET group; PCI did not decrease first isolated BM or improve OS the PET group.	Analyzed BM as a first site of recurrence; Characteristics were not balanced between groups; Less patients underwent MRI in the no-PET group (68.4% vs 82.8%, P=0.001).
	491	Wu, 2017 (15)	BM: Competing risk regression; OS: Cox proportional hazard regression	No vs Yes: Univariate : HR, 0.81; 95% CI, 0.48–1.39, P = 0.45: Multivariate (adjust for stage, chemo): P>0.001.	No vs Yes (adjust for stage, chemo): HR= 0.67; 95%CI: 0.49–0.92; P= 0.014	PCI did not significantly decrease BM, but significantly improved OS in LD-SCLC	
	303	Nakamura, 2018 (21)	BM: χ^2^-test; OS: Cox proportional hazard regression	BM as a first recurrence site: PCI: 18% (17/93); No PCI: 41% (28/69); P=0.002; BM as a first recurrence site time: No PCI: 7.5 months, PCI: 10 months (P = 0.012).	(adjust for age, stage, pulmonary effusion, TDRT/ODRT, SER): HR=0.54, 95%CI: 0.36–0.82, P=0.004.	PCI significantly decreased first isolated BM and improved OS in LD-SCLC	Unbalanced characteristics between PCI and non-PCI group (in no PCI group, more patients had longer SER, more patients had ODRT); χ^2^-test was used for BM analysis; No overall BM results
	203	Kim, 2019 (6)	Cox proportional hazard regression.	HR 0.588, 95% CI 0.338–1.024, P = 0.060. adjust for age, T, and PCI: P=0.068.	whole cohort: PCI: HR 0.543, 95% CI 0.383–0.771, P = 0.001.	PCI improved OS and BMFS in LD-SCLC	Inverse probability treatment weight (IPTW) was used to minimize bias; No report of patients distribution in each group after IPTW; Details of multivariate model not reported.
2) LD-SCLC with MRI: Meta-analysis for BM is not applicable because of different methods.							
	112	Eze, 2017 (35)	BM: Log-rank test; OS: Cox proportional hazard regression	PCI: 16/71 (23%); No PCI: 42/113 (37%); P<0.0001	Yes vs No (adjust for sex, chemo cycles, chemo regimen, response) : HR=1.899; 95% CI, 1.370-2.632; P < 0.0001;	PCI improves OS and decreases BM in LD-SCLC staged with brain MRI	
	342	Pezzi, 2020 (36)	BM: Competing risk regression; OS: Cox proportional hazard regression	3-year BM: PCI 20.40% vs no PCI 11.20%; P = 0.10; No PCI vs PCI (adjust for tumor size, radiation dose): 0.513 (95%CI, 0.239-1.098; P = .09)	No PCI vs PCI (adjust for age, sex, PS, tumor size, radiation dose): HR=0.787; 95%CI, 0.558-1.110; P = 0.17;	PCI does not significantly improve OS or decrease BM in LD-SCLC staged with brain MRI	
3) Resected SCLC: Meta-analysis for BM is not applicable because of no HR data.							
	521	Zhu, 2014 (37)	BM: Log-rank test; OS: Cox proportional hazard regression	2-year BMFS: PCI: 96.8%, non-PCI: 79.4%; 5-year BMFS: PCI: 76.6%, non-PCI: 75.5% (p = 0.014).	2-year OS: All: 73.4%, PCI: 92.5%, non-PCI: 63.2%; 5-year OS: All: 52.3%, PCI: 54.9%, non-PCI: 47.8% (p = 0.001). Yes vs No (adjust for sex, age, KPS, stage, LVI, PORT, chemo cycles): HR= 2.339; 95%CI: 1.414–3.869; P= 0.001. p-stage I: 2-year OS: All: 91.7%, PCI: 100%, non-PCI: 87.1%, 5-year OS: All: 69.3%, PCI: 58.3%, non-PCI: 74.4% (p = 0.601)	PCI improves OS and BMFS in resected LD-SCLC, but not in p-stage I.	
	493	Xu, 2017 (38)	BM: Log-rank test; OS: Cox proportional hazard regression	All: PCI: 15/115 (13.0%), No PCI: 53/234 (22.6%), P=0.009; p-stage I: PCI: 2/19 (10.5%), No PCI: 8/59(13.6%), P=0.389; p-stage II: PCI: 5/39 (12.8%), No PCI:15/67 (22.4%), P=0.094; p-stage III: PCI: 8/57 (14.0%), No PCI: 30/108 (27.8%), P=0.018;	PCI: 36.40 months, 95% CI:23.36–49.44; non–PCI: 25.62 months, 95% CI: 18.86–32.39). No vs Yes (adjust for age, sex, smoking, histology, stage, tumor size, PORT, Surgery type, chemo cycles, and PET/CT scan) HR = 0.69, 95% CI: 0.50–0.95, p= 0.023. p-stage III:HR=0.54, 95% CI: 0.34–0.86, p =0.009). p-stage II: HR=0.54, 95% CI: 0.30–0.99, p =0.047). p-stage I: HR= 1.61, 95% CI: 0.68–3.83, p=0.282).	PCI improves OS and decreases BM in resected LD-SCLC, but not in p-stage I.	
4) ED-SCLC: 2 RCTs have qualified BM data for meta-analysis (415, 445).							
	415^C^	Slotman, 2007 (39) (EORTC)	BM: Competing risk regression; OS: log-rank test	BM prevalence: PCI: 16.8% (24/143); No PCI: 41.3% (59/143); 1-year BM: PCI: 14.6%; No PCI: 40.4%; HR, 0.27; 95%CI, 0.16-0.44; P<0.001.	Median OS: PCI: 6.7 months, No PCI: 5.4 months; HR=0.68; 95% CI, 0.52- 0.88; P = 0.003.	PCI significantly decreased BM and improved OS in ED-SCLC	RCT; Symptomatic BM, no brain images at baseline.
	445^C^	Takahashi, 2017 (40)	BM: Competing risk regression; OS: Cox proportional hazard regression	BM prevalence: PCI: 48% (54/113); No PCI: 69% (77/111); 1-year BM: PCI: 32.9%; No PCI: 59% (HR, 0.49; 95%CI, 0.33-0.74; Gray’s p<0·0001)	Median OS: PCI: 11.6 months, No PCI: 13.7 months;HR=1.27; 95% CI, 0.96–1.68; p=0.094	PCI significantly decreased BM, but did not improve OS in ED-SCLC	RCT; Contains asymptomatic BM, have brain images at baseline.
	80	Chen, 2016 (13)	Cox proportional hazard regression	Yes vs No (adjust for liver metastasis, number of metastatic sites) : HR, 0.410; 95% CI, 0.218–0.770; p< 0.05;	Yes vs No (adjust for PS, liver metastasis, number of metastatic sites) : HR, 0.638; 95% CI, 0.413–0.982; p <0.05;	PCI significantly decreased BM and improved OS in ED-SCLC.	
	28	Bang, 2018 (16)	Cox proportional hazard regression	Yes vs No (adjust for extrathoracic metastases): HR 2.53; 95% CI: 1.51-4.29; P=0.0004);	Yes vs No (adjust for PS, extrathoracic metastases): HR 1.81; 95% CI: 1.29-2.54; P=0.0005	PCI significantly decreased BM and improved OS in ED-SCLC.	Backward stepwise multivariate analysis
5) SCLC							
	18^C^	Arriagada, 1995 (41) (PCI 85)	First isolated BM: Competing risk regression; Overall BM, OS: log-rank test	Overall BM (2-year): PCI: 40%; No PCI: 67%; RR=0.35, P<10^-13^ (Log-rank test); First BM (2-year): PCI: 19%; No PCI: 45%: P<10^-6^ (Gray’s test).	2-year OS: PCI: 29%; No PCI: 21.5%; (adjust for center and stage): RR=0.83, p=0.14	PCI significantly decreased first isolated BM in SCLC, but did not improve OS	RCT; The incidence of first isolated BM is lower than overall BM. Data overlapped with No.19.
	225^C^	Laplanche, 1998 (33) (PCI 88)	First isolated BM: Competing risk regression; Overall BM, OS: log-rank test	Overall BM (4-year): PCI: 44%; No PCI: 51%: RR=0.71, 95%CI 0.45–1.12, P=0.14; First BM (4-year): PCI: 21%; No PCI: 27%: RR=0.69, P=0.26.	4-year OS: PCI: 22%; No PCI: 16%; RR=0.84, p=0.25	PCI did not significantly decrease BM or improve OS in SCLC	RCT; Closed earlier, Power=37%. The incidence of first isolated BM is lower than overall BM. Data overlapped with No.19.
	19^C^	Arriagada, 2002 (42) (PCI 85 + PCI 88)	First isolated BM: Competing risk regression; Overall BM, OS: log-rank test	Overall BM (5-year): PCI: 43%; No PCI: 59%: RR=0.50, P<0.001; First BM (5-year): PCI: 20%; No PCI: 37%: P<0.001.	5-year OS: PCI: 18%; No PCI: 15%; RR=0.84, p=0.06	PCI significantly decreased BM in SCLC, but did not improve OS.	Pooled analysis of 2 RCTs; The incidence of first isolated BM is lower than overall BM; HR is estimated by RR.
	312	Nicholls, 2016 (43)	OS, BMFS: Kaplan-Meier method, Wilcoxon signed-rank test; BM incidence: Fisher’s exact test	LD: PCI: 3 (9.4%), No PCI: 8 (19%), p=0.33; ED: PCI: 4 (23.5%), No PCI: 13 (17.8%), p=0.24 Median BMFS: LD: PCI: 11.8 months (range 11.6–50.2); no PCI: 6.4 months (range 0.2–21.0) (P = 0.22). ED: PCI: 13.6 months (range 8.8–33.1); No PCI: 6.5 months (range 5.2–28.6) (P = 0.04).	LD-SCLC: 8.2 months (0.1–51.5), PCI: 18.8 months (0.9–69.4), No PCI: 8.2 months (0.1–34.4), (P < 0.001). ED-SCLC: 5.7 months (0.1–37.5); PCI: 13.6 months (5.2–37.5), No PCI: 5.6 months (0.1–73.6), (P < 0.001).	PCI improved OS in SCLC	Fisher’s exact test was used for BM incidence analysis.
	439	Suzuki, 2018 (5)	Cox proportional hazard regression.	No vs Yes (adjust for PS, stage, number of extrathoracic metastatic sites, TRT dose, pretreatment LDH, Pretreatment PLR): HR: 0.317, 95% CI: 0.207–0.485, P <0.001	NI	PCI significantly decreases BM in SCLC	
2. PCI dose: ≤25 Gy *vs* > 25 Gy: 2 RCTs have qualified overall BM data for meta-analysis based on Cox regression (148, 231); 2 have overall BM data based on competing risk regression (231, 239); 2 have OS data (231, 239).							
25Gy vs 33Gy	487^C^	Work, 1996 (27)	Log-rank test	5-year BM: 33Gy: 14.9± 7.0%; 25 Gy: 22.9 ± 6.6%; P>0.05	NI	High dose PCI didn’t significantly decrease BM.	RCT;
24Gy vs 36Gy	148 ^C^	Gregor, 1997 (28) (UKCCCR/EORTC)	Log-rank test	2-year BM (data from plot): 36Gy: 16%; 24 Gy: 55%; HR 0.34; 95%CI 0.13–0.86; p<0.05.	NI	High dose PCI decreased BM more effectively in LD-SCLC.	RCT;
25Gy vs 36Gy	231 ^C^	Le Pechoux, 2009 (44)	Overall BM, first isolated BM: Competing risk regression; Overall BM, OS: Cox proportional hazard regression	Overall BM (2-year): 36Gy: 23%; 25Gy: 29%: HR 0.80; 95%CI 0.57–1.11; p=0.18; Overall BM (2-year) (Gray): 36Gy: 16%; 25Gy: 22%: HR= 0.76, 95% CI 0.54–1.05, p=0.10; First BM (2-year) (Gray): 36Gy: 12%; 25Gy: 6%: HR= 0.48, 95% CI 0.29–0.81, p=0.005.	2-year OS: 36Gy: 37%; 25Gy: 42%; HR 1.20; 95%CI 1.00–1.44; p=0.05.	High dose PCI decreased OS and first BM, but did not decrease overall BM in LD-SCLC.	RCT.
≤25 Gy *vs* > 25 Gy	239 ^C^	Levy, 2019 (19) (CONVERT trial)	BM: Competing risk regression; OS: Cox proportional hazard regression	≤25 Gy *vs* > 25 Gy (adjust by Log (tGTV), ODRT/TDRT, Brain MRI/CT, Weight loss, PS, PCI timing): HR: 0.67; 95% CI: 0.34–1.28; P=0.220.	≤25 Gy *vs* > 25 Gy (adjust by Log (tGTV), TDRT vs ODRT, Brain MRI/CT, Weight loss, PS, PCI timing): HR: 0.93; 95% CI: 0.65–1.34; P=0.776.	PCI dose is not a significant risk factor for BM or OS in LD-SCLC with PCI.	Data from RCT
	371	Rubenstein, 1995 (24)	Actuarial survival techniques, log-rank tests.	≤25.2 Gy *vs* > 25.2 Gy: HR: NA, P=0.1091.	NI	PCI dose was not a significant risk factor for BM in LD-SCLC.	Did not report HR.
	52	Brewster, 1995 (45)	Descriptive	Single fraction, 8Gy: 2-yr BM: 22% (16/73); 2-yr BM only: 12.3% (9/73).	2-yr OS: 35%	Single fraction PCI was effective	Included 106 patients, but only 73 with CR were reported for BM incidence,
	513	Zeng, 2019 (10)	Competing-risk regression	lower, standard, higher: HR: 1.09; 95% CI: 0.68–1.73; P=0.73.	NI	PCI dose is not a significant risk factor for BM after PCI in SCLC	
3. PCI timing: Meta-analysis for BM is not applicable because of different analysis methods							
	239 ^C^	Levy, 2019 (19) (CONVERT trial)	BM: Competing risk regression; OS: Cox proportional hazard regression	log(PCI) timing from randomization (adjust by Log (tGTV), ODRT/TDRT, Brain MRI/CT, Weight loss, PS, PCI dose): HR: 1.82; 95% CI: 0.04–8.62; P=0.760	log(PCI) timing from randomization (adjust by Log (tGTV), TDRT vs ODRT, Brain MRI/CT, Weight loss, PS, PCI dose): HR: 0.66; 95% CI: 0.11–4.14; P=0.659	PCI timing from randomization is not a significant risk factor for BM or OS in LD-SCLC with PCI	Data from RCT
	239 ^C^	Levy, 2019 (19) (CONVERT trial)	BM: Competing risk regression; OS: Cox proportional hazard regression	log(PCI) timing from end of CRT (adjust by Log (tGTV), ODRT/TDRT, Brain MRI/CT, Weight loss, PS, PCI dose): HR: 0.83; 95% CI: 0.48–1.45; P=0.520	log(PCI) timing from end of CRT (adjust by Log (tGTV), TDRT vs ODRT, Brain MRI/CT, Weight loss, PS, PCI dose): HR: 1.32; 95% CI: 0.93–1.87; P=0.189	PCI timing from end of CRT is not a significant risk factor for BM or OS in LD-SCLC with PCI	Data from RCT
	239 ^C^	Levy, 2019 (19) (CONVERT trial)	BM: Competing risk regression; OS: Cox proportional hazard regression	log(PCI) timing from beginning of chemo (adjust by Log (tGTV), ODRT/TDRT, Brain MRI/CT, Weight loss, PS, PCI dose): HR: 1.68; 95% CI: 0.03–10.67; P=0.810	log(PCI) timing from beginning of chemo (adjust by Log (tGTV), TDRT vs ODRT, Brain MRI/CT, Weight loss, PS, PCI dose): HR: 1.07; 95% CI: 0.15–7.84; P=0.945	PCI timing from beginning of chemo is not a significant risk factor for BM or OS in LD-SCLC with PCI	Data from RCT
	384	Sas-Korczyńska, 2010 (30)	χ^2^-test;	(early: PCI was given immediately after the end of thoracic radiotherapy and prior to the last cycles of chemotherapy): Early PCI: 3/41 (7.3%), Late PCI: 9/45 (20%), p= 0.00901.	NI	Early PCI is more effective to decrease BM than late PCI in LD-SCLC	χ^2^-test was used for BM analysis.
	356	Ramlov, 2012 (23)	Log- rank test	(Early: <5 months from the diagnosis to PCI): p = 0.26.	NI	PCI timing is not a significant risk factor for BM after PCI in SCLC	No HR reported.
	34	Bernhardt, 2017 (2)	Cox proportional hazard regression	PCI timing from chemo: 120-170 days vs ≤ 120 days: HR 0.91, 95% CI 0.35-2.36, P= 0.85;	PCI timing from chemo: 120-170 days vs ≤ 120 days: HR 0.72, 95% CI 0.40-1.29, P= 0.27;	PCI timing from chemo is not a significant risk factor for BM or OS in ED-SCLC with PCI	No report of patients distribution in each group
	34	Bernhardt, 2017 (2)	Cox proportional hazard regression	PCI timing from brain CT: <80 days *vs* ≥ 80 days: HR 0.52, 95% CI 0.19-1.37, P= 0.18; PCI timing from brain MRI: <80 days *vs* ≥ 80 days: HR 2.30, 95% CI 0.87-6.05, P= 0.09.	PCI timing from brain CT: <80 days *vs* ≥ 80 days: HR 0.62, 95% CI 0.32-1.17, P= 0.14; PCI timing from brain MRI: <80 days *vs* ≥ 80 days: HR 1.49, 95% CI 0.79-2.80, P= 0.21.	PCI timing from brain MRI/CT is not a significant risk factor for BM or OS in ED-SCLC with PCI	No report of patients distribution in each group
	81	Chen, 2018 (8)	BM: Logistic regression. OS: Cox proportional hazard regression	(Early: <6 months from the start of initial chemo to PCI): early PCI: 10/47 (21.3%), late PCI: 23/56 (41.1%); multivariate (adjust for age, sex, PS, tumor load, number of metastatic sites): OR=0.367, 95%CI: 0.145–0.933; P <0.05.	Early vs late: HR=0.917, 95%CI: 0.542–1.551; P=0.748.	Early PCI is more effective to decrease BM than late PCI in ED-SCLC, but not for OS.	Logistic regression was used for BM analysis.
	513	Zeng, 2019 (10)	BM: Competing risk regression; OS: Cox proportional hazard regression	Before vs after completing CRT (adjust for era, PS, stage, ODRT/TDRT, SCRT/CCRT): HR: 1.10; 95% CI: 0.70–1.79; P=0.69.	Before vs after completing CRT (adjust for era, PS, stage, ODRT/TDRT, SCRT/CCRT): HR: 1.37; 95% CI: 1.05–1.78; P=0.02.	Undergoing PCI before completing CRT is an independent risk factor for OS in SCLC with PCI, but not for BM.	
4. TRT vs no TRT: Meta-analysis for BM is not applicable because of different methods and no HR data.							
1) LD-SCLC	519	Zheng, 2018 (9)	Cox proportional hazard regression.	2-year BM: Yes: 41.7%, No: 35.7%; HR: NI, p=0.521.	P=0.182	TRT or not is not a significant risk factor for BM or OS in LD-SCLC	9.2% (14/152) patients did not undergo TRT; Investigated multiple factors (N=21) with limited sample size (n=153).
2) ED-SCLC: Meta-analysis for BM is not applicable because of different statistics							
	526 ^C^	Slotman, 2015 (46) (CREST)	Log-rank test	BM: TRT: 24/247 (9.7%), No TRT: 13/248 (5.2%), p=0.09	2-year OS: TRT: 13%, No TRT: 3%, p=0.004	TRT improved OS, but did not decrease BM in ED-SCLC	RCT;
	140 ^C^	Gore, 2017 (61) (RTOG 0937)	BM: Competing risk regression; OS: Cox proportional hazard regression	1-year BM: No TRT: 17% (95% CI: 6.6– 40.2); TRT: 18.5% (95% CI: 8.5–37.6); P: NI.	No TRT: 15.8 months, 13.8 months, p=0.21 HR:1.44; 95% CI: 0.82–2.53	TRT is not a significant risk factor for OS in ED-SCLC	RCT;
3) Resected SCLC: Meta-analysis for BM is not applicable because of different patients							
	139	Gong, 2013 (11)	Cox proportional hazard regression.	Yes (PORT) vs no (Adjust for stage, histology, induction chemo, adjuvant chemo, and surgical resection): HR= 0.607, 95%CI: NI; P= 0.226.	Yes (PORT) vs no (Adjust for stage, BM, induction chemo, adjuvant chemo, and surgical resection): HR=0.630, 95%CI:NI; P=0.057.	PORT or not is not a significant risk factor for BM in resected LD-SCLC, but tended to improve OS.	Contained many patients with combined SCLC and NSCLC (53.5%, 69/129).); The factors in multivariate model of BM and OS were different.
	520	Zhu, 2014 (4)	Cox proportional hazard regression.	Yes (PORT) vs no (adjust for p-stage and LVI): HR = 0.825, 95%CI: 0.329 ~ 2.064; p = 0.680.	P=0.866	PORT or not is not a significant risk factor for BM or OS in resected LD-SCLC	
5. TRT dose: 2 studies (439, 203) have qualified BM data for meta-analysis, no qualified data for OS meta-analysis.							
	439	Suzuki, 2018 (5)	Cox proportional hazard regression.	<45Gy *vs* ≥ 45Gy (adjust for PS, stage, number of extrathoracic metastatic sites, PCI, pretreatment LDH, Pretreatment PLR): HR: 0.425, 95% CI: 0.267–0.677, P <0.001	NI	Lower TRT dose is an independent risk factor BM in SCLC	
	203	Kim, 2019 (6)	Cox proportional hazard regression.	52.5Gy *vs* 44Gy: HR=0.990, 95%CI: 0.563–1.742, P=0.973;	Adjust for PS, N, stage, TRT dose, LDH, PCI: P>0.05	TRT dose is not a significant risk factor for BM or OS in LD-SCLC	Inverse probability treatment weight (IPTW) was used to minimize bias; No report of patients distribution in each group after IPTW; Details of multivariate model not reported.
6. BED	513	Zeng, 2019 (10)	BM: Competing-risk regression; OS: Cox proportional hazard regression.	(adjust for ODRT/TDRT, SER) HR=1.02, 95%CI:0.97-1.06, P=0.45;	(adjust for ODRT/TDRT, SER) HR=1.02, 95%CI:0.98-1.06, P=0.37;	BED is not a significant risk factor for BM or OS in SCLC with PCI.	
7. TRT timing: Meta-analysis for BM is not applicable because of different methods.							
	488 ^C^	Work, 1997 (59)	Log-rank test	Initial TRT vs delayed 18 weeks: BM prevalence: Early: 11% (11/99); Late: 7% (4/58). 2-year BMFS: Early: 80.8 ± 5.5%; Late: 87.0 ± 6.6% (p=0.24).	Median OS: Early: 10.5 months; Late: 12.0 months, p=0.41	TRT timing is not a significant risk factor for BM or OS in LD-SCLC	RCT;
	532 ^C^	Jeremic, 1997 (72)	Cox proportional hazard regression	CCRT at week 1 vs week 6: 5-year BM: Early TRT: 11%; Late TRT: 10%; P=0.9.	Median OS: Early: 34 months; Late: 26 months. 5-year OS: Early: 30%; Late:15%; *P* = 0.052.	Early TRT improved OS in LD-SCLC, but not significant for BM.	RCT;
	531 ^C^	Skarlos, 2001 (81) (HeCOG)	Cox proportional hazard regression	CCRT at 1^st^ vs 4^th^ chemo: Early TRT: 26% (11/42); Late TRT: 23% (9/39); p>0.05	Death: Early TRT: 69% (29/42); Late TRT: 82% (32/39); P = 0.65.	TRT timing is not a significant risk factor for BM or OS in LD-SCLC	RCT;
	429 ^C^	Spiro, 2006 (66)	Log-rank test	CCRT at 2^nd^ vs 6^th^ chemo: BM: Early: 24%; late: 17%; HR=1.00, 95%CI:0.62-1.61, P=0.12	HR= 1.16; 95% CI, 0.91-1.47; log-rank *P*=0.23.	TRT timing is not a significant risk factor for BM or OS in LD-SCLC	RCT;
	519	Zheng, 2018 (9)	Cox proportional hazard regression.	≤ 2.93 vs > 2.93 months (adjust for smoking, blood glucose, NSE, NLR, T, chemo cycles): HR=0.34, 95%CI: 0.17–0.67, P=0.002.	≤ 2.93 vs > 2.93 months (adjust for NLR) HR= 1.95, 95%CI:1.16-3.26; P= 0.011	Earlier TRT is an independent risk factor for BM in LD-SCLC, but benefits OS.	Authors speculated that earlier TRT might promote metastasis when tumor is larger and active, and the brain is thought to represent a ‘sanctuary’ site as systemic control improves; Investigated multiple factors (N=21) with limited sample size (n=153).
	513	Zeng, 2019 (10)	Competing-risk regression	≤ 64 days vs >64 days: HR=1.09, 95%CI: 0.78–1.53, P=0.62.	NI	TRT timing is not a significant risk factor for BM after PCI in SCLC	
	203	Kim, 2019 (6)	Cox proportional hazard regression.	Early (start TRT at 1^st^ chemo) *vs* late (start TRT at 3^rd^ chemo): HR=1.033, 95%CI: 0.547–1.956, P=0.918.	Adjust for PS, N, stage, TRT dose, LDH, PCI: P>0.05	TRT timing is not a significant risk factor for BM or OS in LD-SCLC	Inverse probability treatment weight (IPTW) was used to minimize bias; No report of patients distribution in each group after IPTW; Details of multivariate model not reported.
8. SER	513	Zeng, 2019 (10)	BM: Competing-risk regression; OS: Cox proportional hazard regression.	(Adjust for ODRT/TDRT, BED) HR=1.00, 95%CI: 1.00-1.01, P=0.58.	(Adjust for ODRT/TDRT, BED) HR=1.00, 95%CI: 1.00-1.01, P=0.14.	SER is not a significant risk factor for BM or OS in SCLC with PCI.	
9. CRT-D	86	Chu, 2019 (17)	Pre-PCI BM: Logistic regression; OS: Cox proportional hazard regression.	(Adjust for smoking, T, and N): OR=1.406, 95%CI: 1.007–1.964, P=0.045	(Adjust for T and N): HR=1.227, 95%CI: 1.026–1.466, P=0.025	CRT-D is an independent risk factor for pre-PCI BM and OS in LD-SCLC	Investigated risk factors for Pre-PCI BM in LD-SCLC using logistic regression.
10. TRT techni-que	115	Farooqi, 2017 (1)	BM: Competing-risk regression. OS: Cox proportional hazard regression.	IMRT *vs* 2D/3D: SHR 0.46, 95% CI 0.29–0.71, P=0.001; Multivariate (adjusted factors: NI): SHR 0.46, 95% CI 0.30–0.73, p=0.001.	Multivariate (adjusted factors: NI): HR 0.79, 95% CI 0.64–0.99, p=0.037	Compared to 2D/3D, IMRT is an independent risk factor for BM and OS in LD-SCLC.	Two definitions for time to development of BM, unclear which one is used
11. Era: Meta-analysis for BM is not applicable because of different methods.							
	115	Farooqi, 2017 (1)	BM: Competing-risk regression. OS: Cox proportional hazard regression.	<2000 *vs* ≥ 2000: SHR 0.57, 95% CI 0.40–0.80, P=0.001; Multivariate (adjusted factors: NI): P>0.05	HR 0.76, 95% CI 0.63–0.90, P=0.002; Multivariate (adjusted factors: NI): P>0.05	Era is not an independent risk factor for BM or OS in LD-SCLC	Two definitions for time to development of BM, unclear which one is used
	28	Bang, 2018 (16)	Cox proportional hazard regression	<2008 *vs* ≥ 2008: P>0.05	<2008 *vs* ≥ 2008: P>0.05	Era is not a significant risk factor for BM or OS in ED-SCLC	Backward stepwise multivariate analysis
	513	Zeng, 2019 (10)	BM: Competing-risk regression; OS: Cox proportional hazard regression.	2003-2010 vs 2011-2016 (adjust for PS, stage, ODRT/TDRT, SCRT/CCRT, PCI timing): HR=0.83, 95% CI 0.55–1.27, p=0.39.	(Adjust for PS, stage, ODRT/TDRT, SCRT/CCRT, PCI timing): HR=0.82, 95% CI 0.65–1.04, p=0.11.	Era is not a significant risk factor for BM or OS in SCLC with PCI	
12. CRT sequence: Meta-analysis for BM is not applicable because of different methods and no HR data.							
1) Alterna-ting vs SCRT	530 ^C^	Gregor, 1997 (78) (EORTC)	Cox proportional hazard regression	First isolated BM: Alternating: 20% (34/169); SCRT: 16% (26/165); P: NI.	Death: Alternating: 81.2% (138/170); SCRT: 81.8% (135/165); P=0.24.	A/S was not a significant factor for OS in LD-SCLC. The significance of difference on BM was unclear.	Analyzed first isolated BM instead of overall BM. HR or P of BM was not reported.
2) CCRT vs SCRT							
	529 ^C^	Takada, 2002 (76) (JCOG 9104)	Cox proportional hazard regression	First isolated BM: SCRT: 27% (31/114); CCRT: 19% (22/114); P=0.16.	Median OS: SCRT:19.7months, CCRT: 27.2 months, P=0.094; (Adjust for PS, stage, age, and sex): HR=0.70, 95%CI: 0.52-0.94, P=0.02.	CCRT significantly improved OS in LD-SCLC, but not for first isolated BM.	Analyzed first isolated BM instead of overall BM.
	108	El Sharouni, 2009 (62)	BM: χ2 test; OS: Log-rank test	SCRT+PCI: 16.4% (11/67); CCRT+PCI: 8.7% (2/23). (P=0.502)	SCRT (N=95): 14.0 months; CCRT (N=40): 21.8 months; P: NI	CCRT/SCRT is not a significant risk factor for BM after PCI in SCLC	χ2 test wasused for BM in SCRT + PCI vs CCRT + PCI but with low number of events. Statistic significance of OS was not reported.
	264	Manapov, 2012 (25)	Log-rank test	BMFS: CCRT: 332 days, SCRT: 267 days, p = 0.522.	NI	CCRT/SCRT is not a significant risk factor for BM in LD-SCLC with poor initial PS	No HR.
	263	Manapov, 2012 (25)	Descriptive	SCRT: 19% (14/74); CCRT:31% (16/51); p: NI.	CCRT: 14.9 months (95% CI 11.7–18.2); SCRT: 16.1 months (95% CI 12.2–20) ; p = 0.6.	In LD-SCLC patients with poor initial PS, more patients developed BM in the CCRT group than in the SCRT group. But the P value was not reported. CCRT/SCRT is not a significant risk factor for OS.	No statistic analysis details and no statistic interpretation.
	265	Manapov, 2013 (49)	Log-rank test	CCRT: 37% (19/51); SCRT:20% (15/74); Log-rank P=0.049. BM time from initial diagnosis: CCRT: 330 days (95%CI: 216-444), SCRT: 273 days (95%CI:221-325), Log-rank P=0.7; from end of chemotherapy: CCRT: 123 days (95%CI:15-231), SCRT: 151 days (95%CI:101-210), Log-rank P=0.7; from end of TRT: CCRT: 213 days (95%CI: 104-322), SCRT: 73 days (95%CI: 17-129), Log-rank P=0.2;	14.9 months (SCRT vs CCRT: P=0.6)	CCRT/SCRT is not a significant risk factor for OS in LD-SCLC. The conclusion of impact on BM is contradictory	The BM conclusion is contradictory with the detailed BM time.
	115	Farooqi, 2017 (1)	BM: Competing-risk regression. OS: Cox proportional hazard regression	CCRT *vs* induction chemo→CRT: SHR 1.36, 95% CI 0.92–2.02, P=0.120; CCRT *vs* induction chemo→RT: SHR 1.14, 95% CI 0.75–1.75, P=0.534.	CCRT vs introduction chemo→CRT): HR 1.55, 95% CI 1.25–1.92, P<0.001. Multivariate (adjusted factors: NI): P>0.05	CCRT/SCRT is not an independent risk factor for BM or OS in LD-SCLC.	Two definitions for time to development of BM, unclear which one is used
	514	Zeng, 2017 (7)	Cox proportional hazard regression.	P=0.163	NI	CCRT/SCRT is not a significant risk factor for BM after PCI in SCLC	
	519	Zheng, 2018 (9)	Cox proportional hazard regression.	P=0.062	P=0.440	CCRT/SCRT is not a significant risk factor for BM or OS in LD-SCLC	Investigated multiple factors (N=21) with limited sample size (n=153).
	513	Zeng, 2019 (10)	BM: Competing-risk regression; OS: Cox proportional hazard regression	(adjust for PS, stage, ODRT/TDRT, era, PCI timing): HR=0.87, 95% CI 0.62–1.23, P=0.42.	(adjust for PS, stage, ODRT/TDRT, era, PCI timing): HR=0.89, 95% CI 0.71–1.11, P=0.30.	CCRT/SCRT is not a significant risk factor for BM or OS in SCLC with PCI.	
13. TRT fractionation: Meta-analysis for BM is not applicable because of different methods and no HR data.							
	239 ^C^	Levy, 2019 (19) (CONVERT trial)	BM: Competing risk regression; OS: Cox proportional hazard regression	TDRT vs ODRT (adjust by Log (tGTV), brain CT/MRI, weight loss, PS, PCI timing, PCI dose): HR: 0.93; 95% CI: 0.57–1.53; P=0.770	TDRT vs ODRT (adjust by Log (tGTV), brain CT/MRI, weight loss, PS, PCI timing, PCI dose): HR: 1.16; 95% CI: 0.89–1.51; P=0.275.	ODRT/TDRT is not a significant risk factor for BM or OS in LD-SCLC with PCI.	Data from RCT
	514	Zeng, 2017 (7)	Cox proportional hazard regression.	ODRT vs TDRT (adjust for sex, age, smoking, response, TNM stage, CCRT/SCRT, chemotherapy cycles, brain CT/MRI): 3-year BM: ODRT: 21%; TDRT: 43%; HR = 2.748, 95%CI 1.227–6.157, p = 0.014	*p* = 0.570	TDRT is an independent risk factor for BM after PCI in SCLC, but not for OS.	
	115	Farooqi, 2017 (1)	BM: Competing-risk regression. OS: Cox proportional hazard regression.	ODRT *vs* TDRT: SHR 1.01, 95%CI 0.72–1.41, P=0.971; ODRT *vs* Mixed: SHR 1.02, 95%CI 0.25–1.45, P=0.981.	HR 0.75, 95%CI 0.63–0.90, P=0.002. Multivariate (adjusted factors: NI): P>0.05	ODRT/TDRT is not an independent risk factor for BM or OS in LD-SCLC.	Two definitions for time to development of BM, unclear which one is used
	519	Zheng, 2018 (9)	Cox proportional hazard regression.	ODRT vs TDRT: P=0.187	P=0.453	ODRT/TDRT is not a significant risk factor for BM or OS in LD-SCLC	13.7%(19/139) were TDRT; Investigated multiple factors (N=21) with limited sample size (n=153).
	303	Nakamura, 2018 (21)	BM: χ^2^-test; OS: Cox proportional hazard regression	BM as a first recurrence site: ODRT: 34% (23/68); TDRT: 23% (22/94); P=0.144.	ODRT vs TDRT (adjust for age, stage, pulmonary effusion, PCI, SER): HR=0.49, 95%CI: 0.27–0.88, P=0.016.	ODRT/TDRT is not a significant risk factor for BM in LD-SCLC, but TDRT improved OS.	No overall BM results. χ^2^-test was used for BM analysis.
	513	Zeng, 2019 (10)	BM: Competing-risk regression; OS: Cox proportional hazard regression.	ODRT vs TDRT (adjust for era, PS, CCRT/SCRT, stage, timing of PCI): HR=1.57, 95%CI: 1.04-2.37, p=0.03; After propensity score matching: ODRT vs TDRT (adjust for BED, SER): HR=1.98, 95%CI: 1.09-3.59, p=0.03.	ODRT vs TDRT (adjust for era, PS, CCRT/SCRT, stage, timing of PCI): HR=1.13, 95%CI: 0.86-1.50, p=0.38;After propensity score matching: ODRT vs TDRT (adjust for BED, SER): HR=1.69, 95%CI: 1.05-2.71, p=0.03.	TDRT is an independent risk factor for BM and OS in SCLC with PCI.	Propensity score matching was used to minimize bias.
14. Treatment intent: Meta-analysis is not applicable because of different methods.							
	371	Rubenstein, 1995 (24)	Multivariate Cox regression	Curative vs not (adjusted factors: PCI, response, age, KPS) HR: NI, P>0.05.	NI	Treatment intention was not a significant risk factor for BM in LD-SCLC.	Did not report HR.
	377	Sahmoun, 2005 (12)	Cox proportional-hazards regression models	CRT vs Chemo alone (adjust for stage, BMI, age, sex, laterality, anatomical site, PCI): HR=2.46, 95%CI: 1.41-4.28; P: NI	CRT vs Chemo alone (adjust for stage, BMI, age, sex, laterality, anatomical site): HR=1.17, 95%CI: 0.74-1.8; P: NI	Compared to CRT, chemo alone is an independent risk factor for BM, but not for OS.	The hazards model of OS did not include PCI.
	377	Sahmoun, 2005 (12)	Cox proportional-hazards regression models	CRT vs No treatment (adjust for stage, BMI, age, sex, laterality, anatomical site, PCI): HR=2.65, 95%CI: 1.26-5.64; P: NI	CRT vs No treatment (adjust for stage, BMI, age, sex, laterality, anatomical site): HR=3.30, 95%CI: 1.87-5.8; P: NI	Compared to CRT, no treatment is an independent risk factor for BM and OS.	The hazards model of OS did not include PCI.
15. Chemo cycles: Meta-analysis for BM is not applicable because of different methods and no HR data.							
	520	Zhu, 2014 (4)	Cox proportional hazard regression.	<4 vs ≥ 4: P= 0.624	P= 0.638	Chemo cycles is not a significant risk factor for BM or OS in resected LD-SCLC	
	439	Suzuki, 2018 (5)	Cox proportional hazard regression.	<4 vs ≥ 4: HR: 0.939, 95%CI: 0.457–1.928; P= 0.863.	NI	Chemo cycles is not a significant risk factor for BM in SCLC	
	519	Zheng, 2018 (9)	Cox proportional hazard regression.	≤4 vs >4 (adjust for smoking, blood glucose, NSE, NLR, T, TRT timing): HR=0.49, 95%CI:0.25–0.95, P= 0.036.	P=0.345	Chemo cycles is a significant risk factor for BM in LD-SCLC, but not for OS.	Investigated multiple factors (N=21) with limited sample size (n=153).
	514	Zeng, 2017 (7)	Cox proportional hazard regression.	≤6 vs >6: P=0.960	NI	Chemo cycles is not a significant risk factor for BM after PCI in SCLC	
	491	Wu, 2017 (15)	BM: Competing risk regression; OS: Cox proportional hazard regression	No vs Yes (Adjust for PCI, Stage):P>0.05	No vs Yes (Adjust for PCI, Stage): HR=0.45, 95%CI: 0.25–0.81, P= 0.008	Chemo did not decrease BM, but improved OS in LD-SCLC	Only 6.7% (17/283) patients did not get chemotherapy.
	28	Bang, 2018 (16)	Cox proportional hazard regression	(Continuous): P>0.05	(Continuous): P>0.05	Chemo cycles is not a significant risk factor for BM or OS in ED-SCLC	Backward stepwise multivariate analysis
	513	Zeng, 2019 (10)	Competing-risk regression	<4, 4-6, >6: HR=1.50, 95%CI: 0.88–2.54; P= 0.13.	NI	Chemo cycles is not a significant risk factor for BM after PCI in SCLC	
16. Chemo regimen: Meta-analysis is not applicable because of different methods.							
	388^C^	Schiller, 2001 (58) (E7593)	Log-rank test	Observation: 25%; Topotecan: 31%. p>0.05	1-year OS: Observation: 28%; Topotecan: 25%; P=0.43	Compared to observation, Topotecan after first line EP chemo did not improve OS or BM in ED-SCLC	
	536^C^	Sundstrøm, 2002 (64)	BM: χ^2^-test; OS: Cox proportional hazard regression	325 of the 436 patients had available follow-up information. 290 were relapsed. 46% recurred in the brain: EP: 57% (82/143); CEV: 46% (68/147); P=0.06	Median OS: EP: 10.2 months; CEV: 7.8 months; P=0.0004.	Compared to CEV, EP improved OS in SCLC.	χ^2^-test was used for BM analysis.
	28	Bang, 2018 (16)	Cox proportional hazard regression	Cisplatin vs Carboplatin: P>0.05	Cisplatin vs Carboplatin: P>0.05	Chemo regimen is not a significant risk factor for BM or OS in ED-SCLC	Backward stepwise multivariate analysis
	513	Zeng, 2019 (10)	Competing-risk regression	EP vs non-EP: HR=1.33, 95%CI: 0.76–2.33; P= 0.32.	NI	Chemo regimen is not a significant risk factor for BM after PCI in SCLC	
	513	Zeng, 2019 (10)	Competing-risk regression	Types of chemo regimen involved (1 vs ≥ 2): HR=1.17, 95%CI: 0.75–1.84; P= 0.48.	NI	Types of chemo regimen involved is not a significant risk factor for BM after PCI in SCLC	
17. chemo or not in resected LD-SCLC							
1). Induction chemo	139	Gong, 2013 (11)	Cox proportional hazard regression.	Yes vs no (Adjust for stage, histology, PORT, adjuvant chemo, and surgical resection): HR= 1.556, 95%CI: NI; P= 0.274.	Yes vs no (Adjust for stage, BM, PORT, adjuvant chemo, and surgical resection): HR=1.201, 95%CI:NI; P=0.423.	Induction chemo or not is not a significant risk factor for BM or OS in resected LD-SCLC.	Contained many patients with combined SCLC and NSCLC (53.5%, 69/129); The factors in multivariate model of BM and OS were different.
2). Adjuvant chemo	139	Gong, 2013 (11)	Cox proportional hazard regression.	Yes vs no (Adjust for stage, histology, induction chemo, PORT, and surgical resection): HR=2.515, 95%CI: NI; P= 0.373.	Yes vs no (Adjust for stage, BM, induction chemo, PORT, and surgical resection): HR=0.524, 95%CI:NI; P=0.067.	Adjuvant chemo or not is not a significant risk factor for BM in resected LD-SCLC, but tended to improve OS.	Only 11.1% (14/126) patients did not undergo adjuvant chemo; Contained many patients with combined SCLC and NSCLC (53.5%, 69/129); The factors in multivariate model of BM and OS were different.
18. Surgery or not	513	Zeng, 2019 (10)	Competing-risk regression	HR=0.75, 95%CI: 0.36–1.58; P= 0.45.	NI	Surgery is not a significant risk factor for BM after PCI in SCLC	Only 5.7% (44/778) patients underwent surgery.
19. Surgical resection complete or not	139	Gong, 2013 (11)	Cox proportional hazard regression.	Complete vs incomplete (Adjust for stage, histology, induction chemo, adjuvant chemo, and PORT): HR=3.563, 95%CI: NI; P=0.020.	Complete vs incomplete (Adjust for stage, BM, induction chemo, adjuvant chemo, and PORT): HR=1.712, 95%CI:NI; P=0.117.	Compared to complete resection, incomplete resection is an independent risk factor for BM, but not for OS in resected LD-SCLC	Contained many patients with combined SCLC and NSCLC (53.5%, 69/129); The factors in multivariate model of BM and OS were different.
20. Brain CT/MRI before PCI: Meta-analysis is not applicable because of different methods.							
	239 ^C^	Levy, 2019 (19) (CONVERT trial)	BM: Competing risk regression; OS: Cox proportional hazard regression	MRI vs CT (adjust by Log (tGTV), ODRT/TDRT, weight loss, PS, PCI timing, PCI dose): HR: 1.28; 95% CI: 0. 67–2.46; P=0.450	MRI vs CT (adjust by Log (tGTV), TDRT vs ODRT, weight loss, PS, PCI timing, PCI dose): HR: 1.41; 95% CI: 0.99–2.00; P=0.151	Brain MRI/CT is not a significant risk factor for BM or OS in LD-SCLC with PCI	Data from RCT
	514	Zeng, 2017 (7)	Cox proportional hazard regression.	MRI vs CT: P=0.362	MRI vs CT: P=0.239	Brain MRI/CT is not a significant risk factor for BM or OS in SCLC with PCI	
	28	Bang, 2018 (16)	Cox proportional hazard regression	MRI vs CT: P>0.05	MRI vs CT: P>0.05	Postchemo brain MRI/CT is not a significant risk factor for BM or OS in ED-SCLC	Backward stepwise multivariate analysis
21. PET-CT or not at diagnosis	82	Choi, 2017 (34)	Cox proportional hazard regression.	cumulative first isolated BM: whole: PET: 38.7%; No PET: 30.1% (P = 0.718); PCI: PET: 34.3%; No PET: 13.3% (P = 0.177); No PCI: PET: 41.1%; No PET: 37.1% (P = 0.942);	5-year OS: whole: PET: 38.2%; No PET: 30.5% (P = 0.023); PCI: PET: 38.3%; No PET: 33.6% (P = 0.985); No PCI: PET: 38.6%; No PET: 29.3% (P = 0.011); Yes vs no (Adjust for age, sex, PS, and PCI): HR=1.452, 95%CI: 1.071-1.968; P=0.016	With initial PET or not did not significantly correlate with first isolated BM in LD-SCLC, but improved OS.	Analyzed BM as a first site of recurrence; Characteristics were not balanced between groups.
22. Treating site (hospital)	513	Zeng, 2019 (10)	Competing-risk regression	HR=0.99, 95%CI: 0.87–1.13; P= 0.86.	NI	Treating hospital is not a significant risk factor for BM after PCI in SCLC	

Notes:

^A^: All the results are in univariate analysis for overall BM unless specified;

^B^: Only factors with BM results will be presented with the OS results;

^C^: Highlighted studies are RCTs.

^D^: Baseline performance status unless specified;

^E^: Response to chemoradiotherapy unless specified.

BED, biologically effective dose; BM, brain metastasis; BMFS, brain metastasis free survival; BMI, body mass index; CCRT, concurrent chemoradiotherapy; CEA, carcinoembryonic antigen; CEV, cyclophosphamide-epirubicin-vincristine; chemo, chemotherapy; CI, confidence interval; CR, complete response; CRT, chemoradiotherapy; CRT-D: Chemoradiotherapy duration; CT, computerized tomography; CTC, circulating tumor cells; ED, extensive-stage disease; EP, etoposide-platinum; HR, hazard ratio; IMRT, intensity-modulated radiotherapy; IPTW, inverse probability treatment weight; IR, incomplete response; KPS, Karnofsky performance status scale; LD, limited-stage disease; LDH, lactate dehydrogenase; LVI, lymphovascular invasion; MRI, magnetic resonance imaging; NA, not applicable; NI, no information; NLR, neutrophil-to-lymphocyte ratio; NR: Non-response; NSCLC, non-small cell lung cancer; NSE, neuron-specific enolase; ODRT, once-daily radiotherapy; OR, odds ratio; OS, overall survival; PCI, prophylactic cranial irradiation; PET-CT, positron emission tomography and computed tomography; PLR, platelet-to-lymphocyte ratio; PORT, postoperative radiotherapy; PS, performance status; SCLC, small cell lung cancer; SCRT, sequential chemoradiotherapy; SD, stable disease; SER, start of any treatment until the end of chest irradiation; SHR, subdistribution hazard ratio; SUV, standardized uptake value, tGTV, thoracic gross tumor volume; TRT, thoracic radiotherapy; TDRT, twice-daily radiotherapy; 2D, two-dimensional radiotherapy; 3D, three-dimensional radiotherapy.

We also found that the definition of time to BM events varied among studies, which indicates that heterogeneity also exists between RCTs: from the date of initial diagnosis (n = 19) (45, 49, 51, 53, 55, 58–62, 64, 66, 71, 72, 74–76, 78, 81); from the date of randomization (n = 16) (5, 9, 25, 26, 28–32, 34, 35, 41, 46, 68, 79, 80); from the date of treatment initiation (n = 6) (37, 42, 47, 57, 69, 77); from the end of chemoradiotherapy (CRT) (n = 5) (44, 47, 67, 70, 78); from the date of PCI (n = 4) (27, 48, 54, 65); from the date of chemotherapy initiation (n = 3) (33, 38, 39); from the date of TRT initiation (n = 2) (43, 56); from the date of surgery (n = 1) (50); five studies had no information (36, 40, 52, 63, 73), two studies applied two definitions (47, 78).

More importantly, we noticed that the statistical analyses for BM varied considerably: Competing risk regression: n = 12 (47, 56, 60, 73), RCT: N = 8 (5, 9, 26–30, 46); Cox proportional hazard regression: n = 20 (37, 38, 43, 45, 48–53, 55, 57–59, 61, 70), RCT: N = 4 (31, 33, 40, 41); Log-rank test n = 16 (43, 44, 62, 64–66, 72, 74, 75, 78), RCT: N = 6 (25, 32, 34, 35, 68, 79); Logistic regression: n = 3 (36, 54, 63); χ^2^-test or Fisher exact 2-tailed test: n = 7 (39, 69, 71, 76, 77), RCT: N = 2 (67, 80); Descriptive: n = 2 (42, 81). Statistical analysis for OS was always performed using survival analysis (Kaplan–Meier, Log-rank test, and Cox regression).

### Risk Factors

In total, 57 factors were reported in all studies, namely, 8 baseline factors, 27 tumor-related factors, and 22 treatment-related factors (**Table 1**). However, they were investigated in various ways with different participants, such as LD, or ED, or resected SCLC, or patients with PCI. Details are shown in the comments in **Table 1**. Hence, 10 factors had qualified BM data from 21 studies (11 RCTs + 10 non-RCTs [all were retrospective studies]) and four factors had qualified OS data for meta-analysis (**Tables 1**, **2**).

**Table 2:** Summary of the 10 factors for BM with meta-analysis.

		BM	
		Risk	Non-significant
OS	Risk	ED	M1b stage
	Non-significant	PCI in ED-SCLC, PCI dose	
	Unclear	Age, Male (p=0.06), cT-stage, PS (p=0.06), PCI in SCLC	Smoking
	No information		TRT dose

BM, brain metastasis; ED, extensive-stage disease; OS, overall survival; PCI, prophylactic cranial irradiation; PS, performance status; SCLC, small cell lung cancer; TRT, thoracic radiotherapy.

#### A. Baseline Characteristics

**Figure 3:**
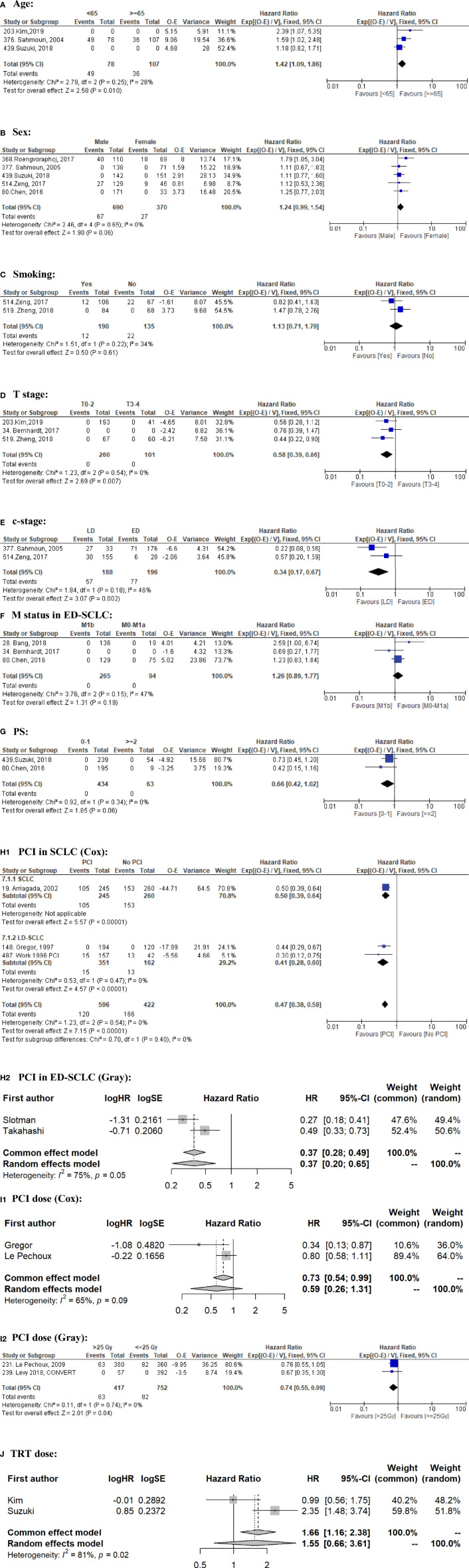
Forrest plots for BM: **(A)** Age; **(B)** Sex; **(C)** Smoking; **(D)** T stage; **(E)** c-stage; **(F)** M status in ED-SCLC; **(G)** PS; **(H1)** PCI in SCLC; **(H2)** PCI in ED-SCLC; **(I1)** PCI dose (Cox); **(I2)** PCI dose (Gray); **(J)** TRT dose. BM, brain metastasis; LD, limited-stage disease; ED, extensive-stage disease; SCLC, small cell lung cancer; PCI, prophylactic cranial irradiation; PS, performance status; TRT, thoracic radiotherapy; O, observed events; E, expected events; V, variance; CI, confidence interval; HR, hazard ratio; SE, standard error.

1. Age: Age was investigated in 18 studies with seven different methods (different age groups, continuous vs group) (**Table 1**). It was concluded that age was not an independent risk factor for BM or OS in 14 studies (36, 38, 43, 47, 48, 51, 53–57, 59–61). Three studies (49, 51, 52) were eligible to perform BM meta-analysis and showed that patients with advanced age (≥65) had less BM than younger patients (HR = 0.70, 95% CI: 0.54–0.92; P = 0.01) (**Figure 3A**).

2. Sex: Sex was investigated in 16 studies. It concluded that sex was not an independent risk factor for BM or OS in 13 studies (36, 38, 47, 49–51, 53–56, 59–61). Five studies (51, 53, 58, 59, 62) were eligible to perform a meta-analysis for BM and showed that male sex tends to be a risk factor for BM (HR = 1.24, 95% CI: 0.99–1.54; P = 0.06) (**Figure 3B**).

3. Smoking: Smoking was investigated in seven studies. It has been shown that smoking is not a significant risk factor for BM or OS (36, 50, 51, 53, 55, 56, 61). Two studies (53, 55) were eligible to perform meta-analysis for BM and showed that smoking (ever vs never) was indeed not a significant risk factor for BM (HR = 1.13, 95% CI: 0.71–1.79; P = 0.61) (**Figure 3C**).

#### B. Tumor Related Factors

**Figure 4:**
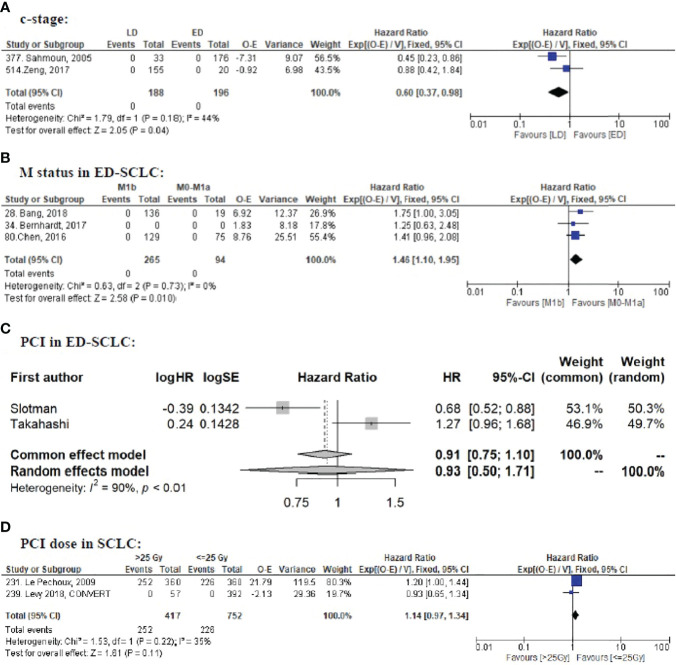
Forrest plots for OS: **(A)** c-stage; **(B)** M status in ED-SCLC; **(C)** PCI in ED-SCLC; **(D)** PCI dose in SCLC. OS, overall survival; LD, limited-stage disease; ED, extensive-stage disease; SCLC, small cell lung cancer; PCI, prophylactic cranial irradiation; O, observed events; E, expected events; V, variance; CI, confidence interval; HR, hazard ratio; SE, standard error.

1. TNM cT stage: The T stage was investigated in four studies with conflicting conclusions (36, 48, 52, 55). Three studies (48, 52, 55) had qualified BM data for meta-analysis and showed that patients with a higher T stage (T ≥3) had a statistically significantly higher risk of BM than patients with lower T stages (HR = 1.72, 95% CI: 1.16–2.56; P = 0.007) (**Figure 3D**).

2. c-stage: c-stage was investigated in different ways in 11 studies with conflicting conclusions (38, 39, 51–53, 55, 56, 58, 60, 64, 65) (**Table 1**). Two studies (53, 58) were eligible to perform meta-analysis for BM and OS. It showed that compared with ED, LD patients had less BM (HR = 0.34, 95% CI: 0.17–0.67; P = 0.002) (**Figure 3E**) and a better OS (HR = 0.60, 95% CI: 0.37–0.98; P = 0.04) (**Figure 4A**).

3. M-status in ED-SCLC: M status (M1b or M0–M1a) was investigated in patients with ED-SCLC in four studies (54, 59, 61, 48). Three were eligible to perform meta-analysis for BM and OS (48, 59, 61). It showed that M1b was a significant risk factor for OS (HR = 1.46, 95% CI: 1.10–1.95; P = 0.01; **Figure 4B**) but not for BM (HR = 1.26, 95% CI: 0.89–1.77; P = 0.19; **Figure 3F**) in ED-SCLC.

4. PS: PS was investigated in 10 studies in different ways. It was concluded that PS was not a significant risk factor for BM or OS in six SCLC studies (38, 51, 52, 54, 55, 63). Two non-RCTs (51, 59) were eligible to perform meta-analysis for BM and showed that better PS (0–1) tended to be associated with less BM (HR = 0.66, 95% CI: 0.42–1.02; P = 0.06) (**Figure 3G**).

#### C. Treatment Related Factors

1. PCI vs no PCI: PCI was investigated in 28 studies, including 8 RCTs. Three RCTs had qualified overall BM data for meta-analysis based on Cox regression (29, 34, 68) and showed that PCI significantly decreases BM in SCLC (HR = 0.47, 95% CI: 0.38–0.58; P <0.00001) and LD-SCLC (HR = 0.41, 95% CI: 0.28–0.60; P <0.00001) (**Figure 3H1**); two had overall BM data based on competing risk regression (5, 9) and also showed that PCI significantly decreased BM in ED-SCLC (HR = 0.37, 95% CI: 0.20–0.65; P = 0.0007) (**Figure 3H2**); two had OS data (5, 9) and showed that PCI did not significantly improve OS in ED-SCLC (HR = 0.93, 95% CI: 0.50–1.71; P = 0.81) (**Figure 4C**). Two retrospective studies (72, 73) investigated PCI in LD-SCLC staged with brain MRI and reported controversial conclusions. Meta-analysis was not applicable. Two retrospective studies (74, 75) investigated PCI in resected LD-SCLC and showed that PCI improved OS and decreased BM in resected LD-SCLC but not in p-stage I. Meta-analysis was also not applicable.

2. PCI dose: PCI dose was investigated in four RCTs (27, 30, 34, 68) and three retrospective studies (42, 43, 56). Two RCTs had qualified overall BM data for meta-analysis based on Cox regression (30, 68) and showed that PCI dose (≤25 Gy vs >25 Gy) was not a significant risk factor for BM (HR = 0.59, 95% CI: 0.26–1.31; P = 0.20) (**Figure 3I1**); two RCTs had overall BM data based on competing risk regression (27, 30) and showed that high dose (>25 Gy) decreased BM more effectively (HR = 0.74, 95% CI: 0.55–0.99; P = 0.04) (**Figure 3I2**); Two had OS data (27, 30) and showed that higher dose did not significantly improve OS (HR = 1.14, 95% CI: 0.97–1.34; P = 0.11) (**Figure 4D**).

3. TRT dose: TRT dose (<45 Gy vs ≥45 Gy) was investigated in patients with SCLC in two studies (51, 52) and obtained different conclusions. Meta-analysis showed that high dose (≥45 Gy) was not a significant risk factor for BM (HR = 1.55, 95% CI: 0.66–3.61; P = 0.31) (**Figure 3J**).

The other 47 factors did not have sufficient qualified data to perform meta-analysis, such as N-stage, number of distant metastasis, and blood biomarkers. Detailed reasons are summarized in **Appendix Text 1**. Detailed results are provided in **Appendix Text 2** along with a brief summary table (**Appendix Table 7**).

## Discussion

Data on risk factors for BM in SCLC are largely lacking, which makes personalized treatment (e.g., shared decision-making regarding PCI) difficult. It also impairs the design and interpretation of RCTs evaluating PCI. We identified several factors that were associated with a higher risk of BM: higher T-stage, ED, male sex, and younger age. As has already been reported previously (4, 82), we also found that PCI reduced BM incidence significantly, but did not improve OS in ED-SCLC. Of note, most data were derived from studies reporting only the development of symptomatic BM since brain imaging before treatment or during follow-up was rarely performed unless indicated by neurological symptoms, indicating that asymptomatic BM data have been missed; and only two RCTs were at low risk of bias. IPD meta-analysis of RCTs could help reveal more clues.

It is not surprising that ED and higher T stage, which means more advanced tumor load, were risk factors for BM. It is more interesting to note that compared to M0–M1a, M1b was a risk factor for OS but not for BM in patients with ED-SCLC. This could be explained by the aggressive nature of ED-SCLC *per se*, resulting in a short OS, making M-status factors less relevant than risk factors for BM development.

We also found younger age (<65) as a risk factor for BM. This is probably because younger SCLC patients generally live longer (50, 58) and therefore have more time to experience BM. Of note, the cut-off value of age varied among studies, but only those age <65 had qualified data to perform meta-analysis in our current study.

Similarly, the cut-off value of PS also varied among studies, resulting in only PS ≥2 having qualified data to perform meta-analysis based on two retrospective studies. It showed that worse PS (≥2) tended to be at a higher risk of BM. This is at odds with a secondary analysis of the CONVERT trial showing that poorer PS (1–2 vs 0) patients had a lower risk (HR: 0.54; 95% CI: 0.32–0.90; P = 0.018) of brain progression (27), likely because they die earlier before developing BM (56, 59, 61).

We also showed a marginally significant risk of developing BM in males. This is consistent with former reports illustrating that female patients had better prognosis than males, in SCLC (62), NSCLC (83), or other cancer sites (84). Reasons for this are not clear, but could include lower proliferation indexes (85), lower levels of p-glycoprotein (86, 87), more frequently expressed thyroid transcription factor-1 (TTF-1) (88), and sex hormone patterns (84).

Furthermore, we found that PCI reduced BM in SCLC but did not improve OS in ED-SCLC, which is based on the EORTC phase III trial (5) and the Japanese phase III trial (9). The conflicting results of these two trials have made PCI in ED-SCLC a reviving area of debate. Details of these two RCTs have been thoroughly discussed in other papers (8, 53, 89). Several literature-based meta-analyses reported conflicting OS results after PCI in ED-SCLC (82, 90, 91). Differences might be explained by including different studies, although all those meta-analyses included the aforementioned two RCTs. Interestingly, the meta-analysis results of two RCTs by Maeng et al. were similar to ours (HR = 0.93, 95% CI: 0.50–1.71; P = 0.81) (82). This also indicates that inclusion criteria for meta-analysis are very crucial and that pooling retrospective studies with RCTs could result in misleading conclusions because of the methodological downsides of retrospective studies.

Interestingly, we noticed that the meta-analysis results based on competing risk regression and Cox regression could be different, which indicates that data based on different statistical analysis methods should not be pooled together to perform meta-analysis. In this current study, only PCI dose (≤25 Gy vs >25 Gy) had qualified data to perform meta-analysis for both regressions. The Cox regression data showed that PCI dose was not a significant risk factor for BM (HR = 0.59, 95% CI: 0.26–1.31; P = 0.20), while the competing risk regression data showed that a higher dose (>25 Gy) could prevent BM more effectively (HR = 0.74, 95% CI: 0.55–0.99; P = 0.04). Of note, both analyses contained the same RCT conducted by Le Pechoux et al. (30), in which the results of competing risk regression (HR = 0.76, 95% CI 0.54–1.05, p = 0.10) and Cox regression (HR = 0.80; 95% CI 0.57–1.11; p = 0.18) were similar. It is unknown whether the meta-analysis results of the same trials would be different. We preferred the competing risk result because it treats death without BM as a competing event. We have not found other systematic reviews or meta-analysis answering the same question. IPD meta-analysis is needed to further clarify these data. Since higher doses of PCI did not improve OS significantly, we do not recommend increasing the PCI dose, especially because a higher PCI dose was associated with a higher risk of cognitive decline (7).

PCI best timing is also unknown. Current guidelines do not have a definite consensus on this issue (89). We identified six studies, which had investigated PCI timing (27, 48, 54, 56, 65, 69). The RCT showed that PCI timing was not a significant risk factor for BM or OS in LD-SCLC (27). Two retrospective studies showed that early PCI was more effective in reducing BM (54, 69), but three others showed the opposite (48, 56, 65). As studies investigated PCI timing in different ways, and the definitions of “early” were also different, there was no qualified data to perform meta-analysis. Therefore, it remains unclear what the best PCI timing is. More RCTs or meta-analysis of RCTs is warranted to further answer this question.

Similarly, four RCTs (31–33, 35) and three retrospective studies (52, 55, 56) have reported the impact of TRT timing on BM with different definitions of “early TRT,” which made the meta-analysis not applicable. Therefore, it is unclear whether TRT timing is a risk factor for BM. However, it has already been shown in an IPD meta-analysis that early TRT (within 30 days after the start of chemotherapy) improves OS (2-year survival: OR: 0.73, 95% CI 0.51–1.03, P = 0.07; 5-year survival: OR: 0.64, 95% CI 0.44–0.92, P = 0.02) (92). Consequently, most guidelines recommend starting TRT in the 1st or 2nd cycle of chemotherapy (89).

Risk of bias assessment is essential in systematic reviews and meta-analyses. We assessed the risk of bias for RCTs using the RoB2 tool and noticed that it has its limitations. It assesses the process of data collection and data reporting but does not assess the methods of data analysis. However, inappropriate analysis can lead to different/misleading conclusions. It also does not evaluate trials that were closed earlier, which results in much less powerful conclusions. Therefore, the improvement of the RoB2 tool is needed to assess the risk of bias more thoroughly and help improve the design of RCTs.

As for the non-RCTs, Wells et al. proposed the Newcastle–Ottawa-Scale (NOS) for assessing the quality on a website rather than in a peer-reviewed journal (93). Till now, NOS has been widely used and tends to become increasingly popular for non-RCTs in meta-analysis. However, a discussion in depth showed that the NOS has unknown validity and that using this score may produce arbitrary results (94). Lo et al. also found that the assessment between reviewers and authors of the studies was very different (95). Interestingly, many studies that used the NOS cited this critical discussion instead of the original web-based link (96–99), suggesting that researchers were using the problematic tool even though they were aware of the limitations.

The Cochrane community recommends the Risk Of Bias In Non-randomized Studies of Interventions (ROBINS-I) tool for assessing the risk of bias in non-RCTs of interventions (100). However, in our study, the baseline characteristics and tumor-related factors are not interventions, so ROBINS-I is inappropriate as well. Additionally, since most of the included RCTs were at high risk of bias and all the RCTs in which BM was the primary endpoint did not perform regular brain imaging examinations during follow-up, we decided not to perform risk of bias assessment for non-RCTs because the additional work would not add much value to the current study.

Additionally, current risk of bias assessment tools mainly assesses the risk of bias per study. This is fine for studies that mainly investigate interventions. However, as a meta-analysis aims to identify all related risk factors, it is necessary to assess the risk of bias per factor in each study. Therefore, we assessed the quality of data per factor, mainly focusing on the analysis methods in each study and summarized the possible problems in the comments. In this way, readers can clearly interpret the results.

As far as we are aware, this is the first systematic review and meta-analysis to identify risk factors for BM in SCLC. Most current meta-analyses focused on one aspect, such as PCI or not in SCLC (101), ED-SCLC (82, 90), and resected SCLC (102). Chen et al. conducted a meta-analysis to identify risk factors for BM in NSCLC (97). Unfortunately, they only searched for observational studies instead of RCTs. They used odds ratios (ORs) rather than HRs to measure the effects. Therefore, the conclusions of this study were not comparable to the current study of identifying risk factors for BM in SCLC. We suggest a well-designed study following the PRISMA guidelines and Cochrane handbook before jumping into meta-analysis by simply pooling everything together.

Additionally, we first used a simple and effective method to assess the quality of data before pooling everything together to perform the meta-analysis. That is, only studies of the same type using the same method with proper statistical analysis should be pooled together under the premise that the patients belong to the same category. This will avoid misleading conclusions based on heterogeneous data.

Furthermore, we noticed that many studies retrieved in our search (46, among which 17 were RCTs) did not report BM-related outcomes. Moreover, brain imaging is often lacking in published studies. To evaluate BM risk factors better, it is very crucial to document baseline characteristics, treatment, as well as adequate and regular brain imaging. Brain imaging should be preferred over MRI, as this is the best imaging modality to detect asymptomatic BM. Regular brain imaging is important in clinical trials, as even after a negative baseline brain MRI, in a study by Manapov et al., the second cranial MRI after completion of chemoradiotherapy revealed asymptomatic BM in 11/40 (32.5%) LD-SCLC complete responders (103). In some RCTs (9, 26, 28, 30, 33), MRI was indeed scheduled at specified time points, but it was generally unreported whether these time points were adhered to, which might influence the results. In this study, only one RCT reported the MRI compliance indirectly. Current trials on SCLC patients without BM are assessing whether MRI surveillance could be non-inferior to (hippocampal-avoidance)-PCI in terms of both OS and neurotoxicity (104, 105), in which the regular brain imaging is scheduled. We hope they will also report their compliance data.

We also noticed that many studies which reported BM data did not report OS data. This hampers the interpretation of clinical significance. For example, if a factor (A) is a risk of BM but not for OS, a factor (B) is a risk of both BM and OS, and another factor (C) is a risk of BM but unknown for OS, clinicians will put much higher weight on considering factor B and much less weight on considering C when making an individualized management strategy. Therefore, we suggest researchers report OS data as well when reporting BM data to enhance the clinical application value.

## Conclusion

In conclusion, multiple studies evaluated risk factors for SCLC BM, but limited data were qualified to perform a meta-analysis. We found that younger age, higher T stage, and ED were risk factors for BM; suggesting that PCI should be especially discussed in such cases, shared decision making is necessary; and that higher PCI dose is not necessary. IPD meta-analysis and well-designed RCTs with high-quality data are needed to identify more risk factors such as blood biomarkers, and confirm our findings. Regular MRI with contrast-enhancement before PCI and during follow-up is helpful to detect asymptomatic BM, especially for patients with a high risk for BM. The MRI compliance at each pre-specified time point should also be reported in prospective trials. Better collaboration with statisticians is needed in future studies. We suggest emendation of the ROB2 tool to assess the statistical methods as well.

## Author Contributions

HZ, DDR, and LH conceived this study. HZ and DDR searched papers in Pubmed. HZ and DZ screening the papers from titles to full texts, extracted the data, and assessed the risk of bias. LH checked the screening, extraction and assessments. HZ, WW, and RH analyzed the results. DDR and LH supervised the whole process. HZ, LH, and DDR draft the manuscript. AL, AT, WW, RH, FMK, and DZ made the revisions. All authors listed have made a substantial, direct, and intellectual contribution to the work and approved it for publication.

## Funding

This research was supported by the following grant: Scholarship of China Scholarship Council (Grant No.: CSC 201909370087).

## Conflict of Interest

The authors declare that the research was conducted in the absence of any commercial or financial relationships that could be construed as a potential conflict of interest.

## Publisher’s Note

All claims expressed in this article are solely those of the authors and do not necessarily represent those of their affiliated organizations, or those of the publisher, the editors and the reviewers. Any product that may be evaluated in this article, or claim that may be made by its manufacturer, is not guaranteed or endorsed by the publisher.
