# Supplementary material for: Risk Factors for Brain Metastases in Patients With Small Cell Lung Cancer: A Systematic Review and Meta-Analysis
Source: Front Oncol. 2022 Jun 10;12:889161. doi: 10.3389/fonc.2022.889161 (PMC9226404; doi:10.3389/fonc.2022.889161)
Supplement: Supplementary file 1 [file DataSheet_1.docx]

**Appendix text 1. Reasons of not performing meta-analysis**

After careful assessment for the data, we found that many factors did not have qualified data to perform meta-analysis. Reasons are shown as follows:

**1. The factor was investigated with the same method in only one study:**

1) Age: ≤ 60 vs > 60;

2) Age: <68 vs ≥ 68;

3) Age: ≤ 58 vs > 58;

4) Age: <58.5 vs ≥ 58.5;

5) Chronic disease;

6) Hypertension;

7) c-stage: I-III vs IV

8) LVI

9) Number of extrathoracic metastatic sites

10) Lung metastasis

11) Anatomical site

12) Neutrophil count

13) TLC, total lymphocyte count

14) Platelet count

15) NSE

16) CEA

17) CTC

18) SUVmax

19) BED

20) SER

21) CRT-D

22) TRT technique

23) chemo or not in resected LD-SCLC

24) Surgery or not

25) Surgical resection complete or not

26) PET-CT or not at diagnosis

27) Treating site (hospital)

**2. The factor was analyzed with different statistics or analysis methods in different studies:**

1) Age: <70 *vs* ≥70;

2) Age: <60 vs ≥60;

3) Age: Continuous:

4) Race/ethnicity;

5) Histology (SCLC vs combined SCLC);

6) Tumor size;

7) N stage;

8) c-stage: I-II vs III;

9) Laterality;

10) KPS;

11) PS: 0 vs 1-2;

12) PS: others;

13) Response;

14) LD-SCLC with MRI: PCI or not;

15) PCI timing;

16) SCLC: TRT vs no TRT;

17) TRT timing;

18) Era;

19) CRT sequence;

20) TRT fractionation;

21) Treatment intent;

22) Chemo cycles;

23) Chemo regimen;

24) Brain CT/MRI before PCI;

**3. The continuous variable was analyzed using different cut-off values:**

1) LDH, lactate dehydrogenase

2) NLR, neutrophil-to-lymphocyte ratio

3) PLR, platelet-to-lymphocyte ratio

**4. No HR data (not reported, and also no enough information to calculate):**

1) Age: <60 vs ≥60;

2) Age: Continuous;

3) Histology (SCLC vs combined SCLC);

4) N stage;

5) c-stage: I-II vs III;

6) PS: 0 vs 1-2;

7) Response;

8) Resected SCLC: PCI vs no PCI;

9) SCLC: TRT vs no TRT;

10) CRT sequence;

11) TRT fractionation;

12) Chemo cycles.

**5. Data overlapped:**

1) BMI;

2) c-stage: ≤IIIA vs ≥IIIB:

1) Laterality.

**6. Different statistical analysis:**

1) Weight loss;

2) p-stage: I,II,III;

3) Number of metastatic sites:

4) Bone metastasis;

5) Liver metastasis;

6) Adrenal metastasis.

**7. Different patients:**

1) resected SCLC: PORT or not
