# Supplementary material for: Risk Factors for Brain Metastases in Patients With Small Cell Lung Cancer: A Systematic Review and Meta-Analysis
Source: Front Oncol. 2022 Jun 10;12:889161. doi: 10.3389/fonc.2022.889161 (PMC9226404; doi:10.3389/fonc.2022.889161)
Supplement: Supplementary file 2 [file DataSheet_2.docx]

**Appendix text 2. Risk factors without meta-analysis**

This is a summary for risk factors that have no qualified data to perform meta-analysis for BM or OS. Detailed data are shown in table 1.

***A. baseline characteristics:***

**1. Race**: Two studies investigated race and showed that race was not a significant risk factor for brain metastasis (BM) or overall survival (OS) (1, 2).

**2. Body mass index (BMI)**: Three studies investigated BMI and showed that BMI (<25 vs ≥ 25 kg/m^2^) was not a significant risk factor for BM(3-5). Two studies have overlapping BM data(3, 4). Therefore, meta-analysis was not performed. The impact of BMI on OS was controversial but no qualified data were available to perform meta-analysis.

**3. Weight loss:** Two studies investigated weight loss with conflicting results^(6, 7)^. The CONVERT trial showed that weight loss >10% was an independent risk factor for OS in limited disease small cell lung cancer (LD-SCLC) with prophylactic cranial irradiation (PCI), but not for BM(7). No data were available to perform Meta-analysis. Therefore, it’s unclear whether weight loss is a risk factor for BM.

**4. Chronic disease:** Zheng *et al* investigated chronic disease and showed that it was not a significant risk factor for BM or OS in LD-SCLC(5).

**5. Hypertension**: Sahmoun *et al* investigated hypertension and showed that it was not a significant risk factor for BM in SCLC(3).

***B. Tumor related factors***

**1. Histology (**SCLC vs combined SCLC**):** Two studies investigated SCLC vs combined SCLC and showed that it was not a significant risk factor for BM or OS(8, 9).

**2. Tumor size:** Four studies investigated tumor size in different ways with conflicting conclusions(1, 5, 7, 10) . The CONVERT trial showed that tGTV, thoracic gross tumor volume (tGTV) was an independent risk factor for BM and OS in LD-SCLC with PCI(7). No qualified data were available to perform Meta-analysis. Therefore, it’s unclear whether tumor size is a risk factor for BM.

**3. N-stage:** Three studies investigated N and showed that N was not a significant risk factor for BM in LD-SCLC(5, 10, 11). No qualified data were available to perform meta-analysis. The impact of N on OS was controversial but no qualified data were available to perform meta-analysis.

**4. p-stage:** Two studies investigated p-stage (I, II, III) and showed that p-stage was an independent risk factor for BM and OS in resected LD-SCLC(8, 12). Of note, both studies analyzed this 3-category variable without setting dummy variables or merging into two categories. Therefore, no qualified data were available to perform meta-analysis.

**5. Lymphovascular node invasion (LVI)**: Zhu *et al* investigated LVI and showed that LVI was an independent risk factor for BM in resected LD-SCLC, but not for OS(12).

**6. Number of metastatic sites**: Chen *et al* investigated number of metastatic sites and showed that it was not a significant risk factor for BM or OS in extensive disease small cell lung cancer (ED-SCLC)(13, 14).

**7.** **Number of extrathoracic metastatic sites:** Suzuki *et al* investigated number of extrathoracic metastatic sites and showed that it was not a significant risk factor BM in SCLC(2).

**8. Metastatic organs**: Two studies investigated bone metastasis, liver metastasis, and adrenal metastasis(6, 13). Chen *et al* investigated lung metastasis as well(13). It showed that bone, adrenal, and lung metastasis were not significant risk factors for BM or OS in ED-SCLC. Liver metastasis was a risk factor for OS, the BM conclusions were conflicting. No qualified data were available for meta-analysis.

**9. Laterality:** Three studies(3, 4, 15) investigated laterality and showed that laterality was not a significant risk factor for BM in SCLC, right SCLC was an independent risk factor for OS. No qualified data were available for meta-analysis.

**10. Anatomical site**: Sahmoun *et al* investigated anatomical site and showed that it was not a significant risk factor for BM or OS in LD-SCLC(4).

**11. Karnofsky performance status (KPS)**: Four studies investigated KPS (≥80 *vs* <80 / ≤ 70 vs > 70) and showed that KPS was not a significant risk factor for BM or OS in LD-SCLC(1, 9, 12, 16). Rubenstein *et al* investigated pre-radiotherapy (RT) KPS (≤ 80 vs > 80) and showed that pre-RT KPS was a significant risk factor for BM and OS in LD-SCLC(17). No qualified data were available for meta-analysis.

**12. Response**: Seven studies investigated response with conflicting conclusions(5, 6, 17-21). As studies investigated response in different ways with different patients, no effective data were available to perform meta-analysis. Therefore, it’s unclear whether response is a risk factor for BM.

**13. Lactate dehydrogenase (LDH):** Two studies investigated pretreatment LDH with different cut-off values and found that LDH was not a significant risk factor for BM or OS in LD-SCLC(2, 10). Meta-analysis was not applicable.

**14. Neutrophil count**: Suzuki *et al* investigated pretreatment and pre-PCI neutrophil count and showed that they were not significant risk factors for BM in SCLC(2).

**15. Total lymphocyte count (TLC):** Suzuki *et al* investigated pretreatment and pre-PCI TLC and showed that higher pre-PCI TLC was an independent risk factor for BM in SCLC but pretreatment TLC was not.

**16. Neutrophil-to-lymphocyte ratio (NLR):** Two studies investigated pretreatment NLR with different cut-off values and got controversial conclusions(2, 5). Meta-analysis was not applicable. Therefore, it’s unclear whether pretreatment NLR is a risk factor for BM in SCLC. Suzuki *et al* also investigate pre-PCI NLR and showed that pre-PCI NLR was not a significant risk factor for BM in SCLC(2).

**17. Platelet count:** Suzuki *et al* investigated pretreatment and pre-PCI platelet count and showed that higher pretreatment platelet count was an independent risk factor for BM in SCLC but pre-PCI platelet count was not(2).

**18. Platelet-to-lymphocyte ratio (PLR):** Two studies investigated pretreatment NLR with different cut-off values and showed that it was not a significant risk factor for BM or OS in SCLC(2, 5). Suzuki *et al* also investigated pre-PCI PLR and showed that lower pre-PCI PLR was an independent risk factor for BM in SCLC(2).

**19. Neuron-specific enolase (NSE):** Zheng *et al* investigated pretreatment NSE and showed that NSE was not a significant risk factor for BM or OS in LD-SCLC(5).

**20. Carcinoembryonic antigen** (**CEA):** Zheng *et al* investigated pretreatment CEA and showed that CEA was not a significant risk factor for BM or OS in LD-SCLC(5).

**21. Blood glucose:** Zheng *et al* investigated pretreatment blood glucose and showed that it was not a significant risk factor for BM or OS in LD-SCLC(5).

**22. Circulating tumor cells (CTC)**: Fu *et al* investigated CTC at baseline, post-first cycle, post-fourth cycle and showed that CTC at baseline was an independent risk factor for BM after PCI in stage III SCLC, while CTC post-first cycle and post-fourth cycle were not(19).

**23. Maximum standardized uptake value (SUVmax)**: Wu *et al* investigated SUVmax and showed that it was not a significant risk factor for BM or OS in LD-SCLC(9).

C. ***Treatment related factors:***

**1. PCI timing**: Six studies investigated PCI timing and got different conclusions(7, 14-16, 22, 23). The only one randomized clinical trial (RCT) showed that PCI timing was not a significant risk factor for BM or OS in LD-SCLC with PCI(7). As studies investigated PCI timing in different ways with different patients, no effective data are available to perform Meta-analysis. Therefore, it’s unclear whether PCI timing is a risk factor for BM.

**2. Thoracic radiotherapy (TRT) vs no TRT**: Zheng *et al* investigated TRT in LD-SCLC, Zhu *et al(12)* and Gong *et al*(8) investigated adjuvant TRT in resected LD-SCLC. Two RCTs(24, 25) investigated TRT in ED-SCLC and got different conclusions. Meta-analysis was not applicable.

**3. Biologically effective dose (BED):** Zeng *et al* investigated BED and showed that BED was not a significant risk factor for BM or OS in SCLC with PCI.

**4. TRT timing**: Four RCTs(26-29) and three retrospective studies(5, 10, 15) investigated TRT timing and got different conclusions. As studies investigated TRT timing in different ways with different patients, no effective data were available to perform meta-analysis. Therefore, it’s unclear whether TRT timing is a risk factor for BM.

**5. Start of any treatment until the end of chest irradiation (SER):** Zeng *et al* investigated SER and found that SER was not a significant risk factor for BM or OS in SCLC with PCI(15).

**6. Chemoradiotherapy duration (CRT-D):** Chu *et al* investigated CRT-D and found that CRT-D was an independent risk factor for pre-PCI BM and OS in LD-SCLC. Of note, they only investigated pre-PCI BM with logistic regression. The BM time definition and the impact of CRT-D on total BM was unclear.

**7. TRT technique:** Farooqi *et al* investigated intensity-modulated radiotherapy (IMRT) vs 2D/3D and found that compared to 2D/3D, IMRT was an independent risk factor for BM and OS in LD-SCLC(1). Of note, they used competing risk analysis but the competing event was inappropriate. They also used two definitions for BM time, it’s unclear which definition was used for the data. Therefore, the impact of TRT technique on total BM was unclear.

**8. Era:** Three studies(1, 15, 18) investigated era and showed that it was not a significant risk factor for BM or OS in SCLC. Meta-analysis was not applicable.

**9. Chemoradiotherapy (CRT) sequences**: One RCT(30) investigated the impact of alternating CRT vs sequential CRT (SCRT) on first isolated BM in LD-SCLC and showed that it was not a significant factor for OS in LD-SCLC. The significance of difference on first isolated BM was unclear. One RCT(31) investigated the impact of SCRT vs concurrent CRT (CCRT) on first isolated BM in LD-SCLC and showed that CCRT significantly improved OS in LD-SCLC, but not for first isolated BM. Eight retrospective studies(1, 5, 15, 20, 21, 32-34) showed that SCRT or CCRT was not a significant risk factor for BM or OS in SCLC. Meta-analysis was not applicable.

**10. TRT fractionation**: One RCT(7) investigated once-daily radiotherapy (ODRT) vs twice-daily radiotherapy (TDRT) and showed that ODRT/TDRT was not a significant risk factor for BM or OS in LD-SCLC with PCI. Five retrospective studies(1, 5, 15, 21, 35) got conflicting conclusions. As studies investigated TRT fractionation in different ways with different patients, no effective data were available to perform meta-analysis. Therefore, it’s unclear whether TRT fractionation is a risk factor for BM.

**11. Treatment intent:** Rubenstein *et al* investigated curative vs not and found that it was not a significant risk factor for BM or OS in LD-SCLC(17). Sahmoun *et al* investigated CRT vs chemotherapy (chemo) alone and CRT vs no treatment(4). It showed that compared to CRT, no treatment was an independent risk factor for BM and OS. Compared to CRT, chemo alone was an independent risk factor for BM, but not for OS.

**12. Chemo cycles:** Seven studies(2, 5, 9, 12, 15, 18, 21) investigated chemo cycles with conflicting conclusions. As studies investigated it in different ways with different patients, no effective data were available to perform Meta-analysis. Therefore, it’s unclear whether chemo cycles is a risk factor for BM.

**13. Chemo regimen**: One RCT(36) investigated topotecan after first line etopside-platinum (EP) chemo and showed that compared to observation, topotecan after first line EP chemo did not improve OS or BM in ED-SCLC. One RCT(37) investigated EP vs cyclophosphamide-epirubicin-vincristine (CEV) in SCLC and found that EP improved OS. Zeng *et al* investigated EP vs non-EP and types of chemo regimen involved(15). Bang *et al* investigated cisplatin vs carboplatin(18). It showed that chemo regimen and types of chemo regimen were not significant for BM or OS in SCLC. Meta-analysis was not applicable.

**14. Chemo or not in resected LD-SCLC**: Gong *et al* investigated induction chemo and adjuvant chemo in resected LD-SCLC(8). It showed that induction chemo or not was not a significant risk factor for BM or OS in resected LD-SCLC. Adjuvant chemo or not was not a significant risk factor for BM in resected LD-SCLC, but tended to improve OS. Of note, only 11.1% patients did not undergo adjuvant chemo and the majority patients were combined SCLC (53.5%). Therefore, it’s unclear whether induction and adjuvant chemo was a risk factor for BM or OS in pure LD-SCLC with surgery.

**15. Surgery**: Zeng *et al* investigated surgery and found that surgery was not a significant risk factor for BM after PCI in SCLC(15). Of note, only 5.7% (44/778) patients underwent surgery. Therefore, it’s unclear whether surgery was a risk factor for BM in SCLC.

**16. Surgical resection complete or not**: Gong *et al* investigated surgical resection and found that compared to complete resection, incomplete resection was an independent risk factor for BM in resected LD-SCLC, but not for OS(8). Of note, the majority patients were combined SCLC patients (53.5%); The factors in multivariate model of BM and OS were different. Therefore, it’s unclear whether induction and adjuvant chemo was a risk factor for BM.

**17. Brain CT/MRI before PCI**: One RCT(7) and 2 retrospective studies(18, 21) investigated brain CT vs MRI before PCI and found that it was not a significant risk factor for BM or OS in SCLC with PCI. Meta-analysis was not applicable.

**18. PET-CT or not at diagnosis**: Choi *et al* investigated with or without PET-CT at staging and found that with initial PET or not did not significantly correlate with first isolated BM, but improved OS. Of note, this study only analyzed BM as a first site of recurrence and characteristics were not balanced between groups. Therefore, it is unclear whether PET-CT at staging was associated with total BM in LD-SCLC.

**19. Hospital**: Zeng *et al* investigated treating hospital and found it was not a significant risk factor for BM after PCI in SCLC(15).

**References:**

1. Farooqi AS, Holliday EB, Allen PK, Wei X, Cox JD, Komaki R. Prophylactic cranial irradiation after definitive chemoradiotherapy for limited-stage small cell lung cancer: Do all patients benefit? Radiother Oncol. 2017;122(2):307-12. doi:10.1016/j.radonc.2016.11.012.

2. Suzuki R, Wei X, Allen PK, Welsh JW, Komaki R, Lin SH. Hematologic variables associated with brain failure in patients with small-cell lung cancer. Radiother Oncol. 2018;128(3):505-12. doi:10.1016/j.radonc.2018.05.026.

3. Sahmoun AE, Case LD, Chavour S, Kareem S, Schwartz GG. Hypertension and risk of brain metastasis from small cell lung cancer: a retrospective follow-up study. Anticancer Res. 2004;24(5b):3115-20.

4. Sahmoun AE, Case LD, Santoro TJ, Schwartz GG. Anatomical distribution of small cell lung cancer: effects of lobe and gender on brain metastasis and survival. Anticancer Res. 2005;25(2a):1101-8.

5. Zheng Y, Wang L, Zhao W, Dou Y, Lv W, Yang H, et al. Risk factors for brain metastasis in patients with small cell lung cancer without prophylactic cranial irradiation. Strahlentherapie und Onkologie : Organ der Deutschen Rontgengesellschaft [et al]. 2018;194(12):1152-62. doi:10.1007/s00066-018-1362-7.

6. Greenspoon JN, Evans WK, Cai W, Wright JR. Selecting patients with extensive-stage small cell lung cancer for prophylactic cranial irradiation by predicting brain metastases. J Thorac Oncol. 2011;6(4):808-12. doi:10.1097/JTO.0b013e31820d782d.

7. Levy A, Le Péchoux C, Mistry H, Martel-Lafay I, Bezjak A, Lerouge D, et al. Prophylactic Cranial Irradiation for Limited-Stage Small-Cell Lung Cancer Patients: Secondary Findings From the Prospective Randomized Phase 3 CONVERT Trial. J Thorac Oncol. 2019;14(2):294-7. doi:10.1016/j.jtho.2018.09.019.

8. Gong L, Wang QI, Zhao L, Yuan Z, Li R, Wang P. Factors affecting the risk of brain metastasis in small cell lung cancer with surgery: is prophylactic cranial irradiation necessary for stage I-III disease? International journal of radiation oncology, biology, physics. 2013;85(1):196-200. doi:10.1016/j.ijrobp.2012.03.038.

9. Wu AJ, Gillis A, Foster A, Woo K, Zhang Z, Gelblum DY, et al. Patterns of failure in limited-stage small cell lung cancer: Implications of TNM stage for prophylactic cranial irradiation. Radiother Oncol. 2017;125(1):130-5. doi:10.1016/j.radonc.2017.07.019.

10. Kim TG, Pyo H, Ahn YC, Noh JM, Oh D. Role of prophylactic cranial irradiation for elderly patients with limited-disease small-cell lung cancer: inverse probability of treatment weighting using propensity score. J Radiat Res. 2019;60(5):630-8. doi:10.1093/jrr/rrz040.

11. Chu X, Li S, Xia B, Chu L, Yang X, Ni J, et al. Patterns of brain metastasis immediately before prophylactic cranial irradiation (PCI): implications for PCI optimization in limited-stage small cell lung cancer. Radiation oncology (London, England). 2019;14(1):171. doi:10.1186/s13014-019-1371-4.

12. Zhu H, Bi Y, Han A, Luo J, Li M, Shi F, et al. Risk factors for brain metastases in completely resected small cell lung cancer: a retrospective study to identify patients most likely to benefit from prophylactic cranial irradiation. Radiation oncology (London, England). 2014;9:216. doi:10.1186/1748-717x-9-216.

13. Chen Y, Li J, Hu Y, Zhang Y, Lin Z, Zhao Z, et al. Prophylactic cranial irradiation could improve overall survival in patients with extensive small cell lung cancer : A retrospective study. Strahlentherapie und Onkologie : Organ der Deutschen Rontgengesellschaft [et al]. 2016;192(12):905-12. doi:10.1007/s00066-016-1038-0.

14. Chen Y, Li J, Zhang Y, Hu Y, Zhang G, Yan X, et al. Early versus late prophylactic cranial irradiation in patients with extensive small cell lung cancer. Strahlentherapie und Onkologie : Organ der Deutschen Rontgengesellschaft [et al]. 2018;194(10):876-85. doi:10.1007/s00066-018-1307-1.

15. Zeng H, Li R, Hu C, Qiu G, Ge H, Yu H, et al. Association of Twice-Daily Radiotherapy With Subsequent Brain Metastases in Adults With Small Cell Lung Cancer. JAMA network open. 2019;2(5):e190103. doi:10.1001/jamanetworkopen.2019.0103.

16. Bernhardt D, Adeberg S, Bozorgmehr F, Opfermann N, Hoerner-Rieber J, Repka MC, et al. Nine-year Experience: Prophylactic Cranial Irradiation in Extensive Disease Small-cell Lung Cancer. Clin Lung Cancer. 2017;18(4):e267-e71. doi:10.1016/j.cllc.2016.11.012.

17. Rubenstein JH, Dosoretz DE, Katin MJ, Blitzer PH, Salenius SA, Floody PA, et al. Low doses of prophylactic cranial irradiation effective in limited stage small cell carcinoma of the lung. International journal of radiation oncology, biology, physics. 1995;33(2):329-37. doi:10.1016/0360-3016(95)00166-v.

18. Bang A, Kendal WS, Laurie SA, Cook G, MacRae RM. Prophylactic Cranial Irradiation in Extensive Stage Small Cell Lung Cancer: Outcomes at a Comprehensive Cancer Centre. International journal of radiation oncology, biology, physics. 2018;101(5):1133-40. doi:10.1016/j.ijrobp.2018.04.058.

19. Fu L, Liu F, Fu H, Liu L, Yuan S, Gao Y, et al. Circulating tumor cells correlate with recurrence in stage III small-cell lung cancer after systemic chemoradiotherapy and prophylactic cranial irradiation. Jpn J Clin Oncol. 2014;44(10):948-55. doi:10.1093/jjco/hyu109.

20. Manapov F, Klöcking S, Niyazi M, Levitskiy V, Belka C, Hildebrandt G, et al. Primary tumor response to chemoradiotherapy in limited-disease small-cell lung cancer correlates with duration of brain-metastasis free survival. J Neurooncol. 2012;109(2):309-14. doi:10.1007/s11060-012-0894-4.

21. Zeng H, Xie P, Meng X, Yuan S, Sun X, Li W, et al. Risk factors for brain metastases after prophylactic cranial irradiation in small cell lung cancer. Scientific reports. 2017;7:42743. doi:10.1038/srep42743.

22. Ramlov A, Tietze A, Khalil AA, Knap MM. Prophylactic cranial irradiation in patients with small cell lung cancer. A retrospective study of recurrence, survival and morbidity. Lung cancer (Amsterdam, Netherlands). 2012;77(3):561-6. doi:10.1016/j.lungcan.2012.05.101.

23. Sas-Korczyńska B, Korzeniowski S, Wójcik E. Comparison of the effectiveness of "late" and "early" prophylactic cranial irradiation in patients with limited-stage small cell lung cancer. Strahlentherapie und Onkologie : Organ der Deutschen Rontgengesellschaft [et al]. 2010;186(6):315-9. doi:10.1007/s00066-010-2088-3.

24. Slotman BJ, van Tinteren H, Praag JO, Knegjens JL, El Sharouni SY, Hatton M, et al. Use of thoracic radiotherapy for extensive stage small-cell lung cancer: a phase 3 randomised controlled trial. Lancet. 2015;385(9962):36-42. doi:10.1016/s0140-6736(14)61085-0.

25. Gore EM, Hu C, Sun AY, Grimm DF, Ramalingam SS, Dunlap NE, et al. Randomized Phase II Study Comparing Prophylactic Cranial Irradiation Alone to Prophylactic Cranial Irradiation and Consolidative Extracranial Irradiation for Extensive-Disease Small Cell Lung Cancer (ED SCLC): NRG Oncology RTOG 0937. J Thorac Oncol. 2017;12(10):1561-70. doi:10.1016/j.jtho.2017.06.015.

26. Spiro SG, James LE, Rudd RM, Trask CW, Tobias JS, Snee M, et al. Early compared with late radiotherapy in combined modality treatment for limited disease small-cell lung cancer: a London Lung Cancer Group multicenter randomized clinical trial and meta-analysis. Journal of clinical oncology : official journal of the American Society of Clinical Oncology. 2006;24(24):3823-30. doi:10.1200/jco.2005.05.3181.

27. Work E, Nielsen OS, Bentzen SM, Fode K, Palshof T. Randomized study of initial versus late chest irradiation combined with chemotherapy in limited-stage small-cell lung cancer. Aarhus Lung Cancer Group. Journal of clinical oncology : official journal of the American Society of Clinical Oncology. 1997;15(9):3030-7. doi:10.1200/jco.1997.15.9.3030.

28. Jeremic B, Shibamoto Y, Acimovic L, Milisavljevic S. Initial versus delayed accelerated hyperfractionated radiation therapy and concurrent chemotherapy in limited small-cell lung cancer: a randomized study. Journal of clinical oncology : official journal of the American Society of Clinical Oncology. 1997;15(3):893-900. doi:10.1200/jco.1997.15.3.893.

29. Skarlos DV, Samantas E, Briassoulis E, Panoussaki E, Pavlidis N, Kalofonos HP, et al. Randomized comparison of early versus late hyperfractionated thoracic irradiation concurrently with chemotherapy in limited disease small-cell lung cancer: a randomized phase II study of the Hellenic Cooperative Oncology Group (HeCOG). Annals of oncology : official journal of the European Society for Medical Oncology. 2001;12(9):1231-8. doi:10.1023/a:1012295131640.

30. Gregor A, Drings P, Burghouts J, Postmus PE, Morgan D, Sahmoud T, et al. Randomized trial of alternating versus sequential radiotherapy/chemotherapy in limited-disease patients with small-cell lung cancer: a European Organization for Research and Treatment of Cancer Lung Cancer Cooperative Group Study. Journal of clinical oncology : official journal of the American Society of Clinical Oncology. 1997;15(8):2840-9. doi:10.1200/jco.1997.15.8.2840.

31. Takada M, Fukuoka M, Kawahara M, Sugiura T, Yokoyama A, Yokota S, et al. Phase III study of concurrent versus sequential thoracic radiotherapy in combination with cisplatin and etoposide for limited-stage small-cell lung cancer: results of the Japan Clinical Oncology Group Study 9104. Journal of clinical oncology : official journal of the American Society of Clinical Oncology. 2002;20(14):3054-60. doi:10.1200/jco.2002.12.071.

32. El Sharouni SY, Kal HB, Barten-Van Rijbroek A, Struikmans H, Battermann JJ, Schramel FM. Concurrent versus sequential chemotherapy and radiotherapy in limited disease small cell lung cancer: a retrospective comparative study. Anticancer Res. 2009;29(12):5219-24.

33. Manapov F, Klöcking S, Niyazi M, Belka C, Hildebrandt G, Fietkau R, et al. Chemoradiotherapy duration correlates with overall survival in limited disease SCLC patients with poor initial performance status who successfully completed multimodality treatment. Strahlentherapie und Onkologie : Organ der Deutschen Rontgengesellschaft [et al]. 2012;188(1):29-34. doi:10.1007/s00066-011-0016-9.

34. Manapov F, Klöcking S, Niyazi M, Oskan F, Niemöller OM, Belka C, et al. Timing of failure in limited disease (stage I-III) small-cell lung cancer patients treated with chemoradiotherapy: a retrospective analysis. Tumori. 2013;99(6):656-60. doi:10.1700/1390.15452.

35. Nakamura M, Onozawa M, Motegi A, Hojo H, Zenda S, Nakamura N, et al. Impact of prophylactic cranial irradiation on pattern of brain metastases as a first recurrence site for limited-disease small-cell lung cancer. J Radiat Res. 2018;59(6):767-73. doi:10.1093/jrr/rry066.

36. Schiller JH, Adak S, Cella D, DeVore RF, 3rd, Johnson DH. Topotecan versus observation after cisplatin plus etoposide in extensive-stage small-cell lung cancer: E7593--a phase III trial of the Eastern Cooperative Oncology Group. Journal of clinical oncology : official journal of the American Society of Clinical Oncology. 2001;19(8):2114-22. doi:10.1200/jco.2001.19.8.2114.

37. Sundstrøm S, Bremnes RM, Kaasa S, Aasebø U, Hatlevoll R, Dahle R, et al. Cisplatin and etoposide regimen is superior to cyclophosphamide, epirubicin, and vincristine regimen in small-cell lung cancer: results from a randomized phase III trial with 5 years' follow-up. Journal of clinical oncology : official journal of the American Society of Clinical Oncology. 2002;20(24):4665-72. doi:10.1200/jco.2002.12.111.
