# Supplementary material for: Risk Factors for Brain Metastases in Patients With Small Cell Lung Cancer: A Systematic Review and Meta-Analysis
Source: Front Oncol. 2022 Jun 10;12:889161. doi: 10.3389/fonc.2022.889161 (PMC9226404; doi:10.3389/fonc.2022.889161)
Supplement: Supplementary file 3 [file Table_1.docx]

| **Supplementary Table 1.** PICO searching strategy | |
| --- | --- |
| **PICO** | **Search terms** |
| Patients | (("Carcinoma, Small Cell"[Majr] AND "Lung Neoplasms"[Majr]) OR "Small Cell Lung Carcinoma"[Mesh] OR sclc*[ti] OR (small cell*[ti] AND lung*[ti]) OR (small*[ti] AND cell*[ti] AND lung*[ti]) OR (oat cell*[ti] AND lung*[ti]) OR ("oat"[ti] AND cell*[ti] AND lung*[ti]) OR ((pneumoa*[ti] OR pneumob*[ti] OR pneumoc*[ti] OR pneumod*[ti] OR pneumoe*[ti] OR pneumof*[ti] OR pneumog*[ti] OR pneumoh*[ti] OR pneumoi*[ti] OR pneumok*[ti] OR pneumol*[ti] OR pneumom*[ti] OR pneumon*[ti] OR pneumoo*[ti] OR pneumop*[ti] OR pneumor*[ti] OR pneumos*[ti] OR pneumot*[ti] OR pneumou*[ti] OR pneumov*[ti] OR pneumow*[ti] OR pneumox*[ti] OR pulmon*[ti] OR respir*[ti] OR lung*[ti] OR bronche*[ti] OR bronchi*[ti] OR bronchoa*[ti] OR bronchob*[ti] OR bronchoc*[ti] OR bronchod*[ti] OR bronchoe*[ti] OR bronchof*[ti] OR bronchog*[ti] OR bronchoh*[ti] OR bronchoi*[ti] OR bronchok*[ti] OR bronchol*[ti] OR bronchom*[ti] OR bronchon*[ti] OR bronchoo*[ti] OR bronchop*[ti] OR bronchor*[ti] OR bronchos*[ti] OR bronchot*[ti] OR bronchou*[ti] OR bronchov*[ti] OR bronchoz*[ti] OR bronchu*[ti] OR endobronch*[ti] OR alveol*[ti] OR pleur*[ti] OR diaphragm*[ti] OR diaphragm*[ti] OR thorax*[ti] OR thorac*[ti] OR chest*[ti]) AND ((small*[ti] AND cell*[ti]) OR oat cell*[ti] OR ("oat"[ti] AND cell*[ti]))) NOT "non-small"[ti]) |
| Intervention | NA |
| Comparison | NA |
| Outcome | (("brain metasta*") OR ("cranial metasta*") OR ("CNS metasta*") OR ("central nervous system metasta*") OR ("cerebral metasta*") OR "Brain Neoplasms/secondary" [Mesh] OR ("metastatic brain tum*") OR ("secondary brain tum*")OR ("intra-axial metastatic tum*")) |

| **Supplementary** **Table 2.** Descriptions of the components of PICO | | |
| --- | --- | --- |
| **Acronym** | **Definition** | **Description** |
| P | Patients | SCLC patients without BM at baseline |
| I | Intervention | NA |
| C | Comparison | NA |
| O | Outcome | BM during or after antitumor treatment (follow-up BM), time to BM development, and risk factors associated with BM, overall survival |
| *Abbreviations*: BM, Brain metastasis; SCLC, small cell lung cancer. | | |

| **Supplementary Table 3.** Inclusion criteria | |
| --- | --- |
| Subjects included | Human only |
| Language | English |
| Article type | Original article, full paper |
| Study type | Large scale retrospective studies (sample size ≥ 100);  Prospective observational studies (sample size ≥ 100);  Prospective randomized phase II trials (sample size ≥ 50);  Prospective randomized phase III-IV trials |
| Primary tumor | Small cell lung cancer without brain metastasis at baseline |
| Period | Studies published since 01.01.1995 (as from 1995, brain MRI with gadolinium became more widely available) |
| Follow up period | All |
| Outcome | Brain metastasis |
