# Supplementary material for: Risk Factors for Brain Metastases in Patients With Small Cell Lung Cancer: A Systematic Review and Meta-Analysis
Source: Front Oncol. 2022 Jun 10;12:889161. doi: 10.3389/fonc.2022.889161 (PMC9226404; doi:10.3389/fonc.2022.889161)
Supplement: Supplementary file 4 [file Table_2.docx]

| **Appendix** **Table 2.** Descriptions of the components of PICO | | |
| --- | --- | --- |
| **Acronym** | **Definition** | **Description** |
| P | Patients | SCLC patients without BM at baseline |
| I | Intervention | NA |
| C | Comparison | NA |
| O | Outcome | BM during or after antitumor treatment (follow-up BM), time to BM development, and risk factors associated with BM, overall survival |
| *Abbreviations*: BM, Brain metastasis; SCLC, small cell lung cancer. | | |
