# Supplementary material for: Risk Factors for Brain Metastases in Patients With Small Cell Lung Cancer: A Systematic Review and Meta-Analysis
Source: Front Oncol. 2022 Jun 10;12:889161. doi: 10.3389/fonc.2022.889161 (PMC9226404; doi:10.3389/fonc.2022.889161)
Supplement: Supplementary file 5 [file Table_3.docx]

| **Appendix Table 3.** Inclusion criteria | |
| --- | --- |
| Subjects included | Human only |
| Language | English |
| Article type | Original article, full paper |
| Study type | Large scale retrospective studies (sample size ≥ 100);  Prospective observational studies (sample size ≥ 100);  Prospective randomized phase II trials (sample size ≥ 50);  Prospective randomized phase III-IV trials |
| Primary tumor | Small cell lung cancer without brain metastasis at baseline |
| Period | Studies published since 01.01.1995 (as from 1995, brain MRI with gadolinium became more widely available) |
| Follow up period | All |
| Outcome | Brain metastasis |
