# Supplementary material for: Risk Factors for Brain Metastases in Patients With Small Cell Lung Cancer: A Systematic Review and Meta-Analysis
Source: Front Oncol. 2022 Jun 10;12:889161. doi: 10.3389/fonc.2022.889161 (PMC9226404; doi:10.3389/fonc.2022.889161)
Supplement: Supplementary file 6 [file Table_4.docx]

Appendix Table 4. Design of included randomized controlled trials

| ID | First Author  (Trial) | Journal | Study design | Brain CT or MRI before treatment | Brain CT or MRI before PCI | Scheduled Brain CT or MRI during  follow-up | Brain image contrast-enhanced or not | BM as primary or secondary endpoints | Recruitment  period | Sample size  (planned and actual enrollment) |
| --- | --- | --- | --- | --- | --- | --- | --- | --- | --- | --- |
|  | 1. PCI vs no PCI |  |  |  |  |  |  |  |  |  |
|  | 1) LD-SCLC |  |  |  |  |  |  |  |  |  |
| 487 | Work, 1996(1) | J Clin Oncol | LD-SCLC:  PCI vs no PCI | Only performed  when indicated | Only performed  when indicated. | Only performed  when indicated. | NI | NI | 03-1981 ~  09-1989 | 100-> 200;  199 were eligible,  PCI: 157;  No PCI: 42. |
| 148 | Gregor, 1997(2)  (UKCCCR/EORTC) | Eur J Cancer | Phase III,  LD-SCLC, CR after induction therapy:  PCI vs no PCI  (PCI 24Gy vs 36Gy) | NI | 16% patients had brain CT before PCI | No | NI | Secondary | 10-1987 ~  04-1995 | 300 required (Power: NI)  314 patients (194 PCI, 120 No PCI) were randomized. |
| 62 | Cao, 2005(3) | Chin Med J (Engl) | LD-SCLC:  PCI vs no PCI | CT?^A^ | CT?^A^ | NI | NI | NI | 01-1990 ~  12-1995 | NI for targeted size;  51 enrolled: 26 PCI, 25 no PCI |
|  | 2) ED-SCLC |  |  |  |  |  |  |  |  |  |
| 415 | Slotman, 2007(4) (EORTC) | N Engl J Med | Phase III,  ED-SCLC:  PCI vs no PCI | No | No | Only performed  when indicated | Yes | Primary | 02-2001 ~  03-2006 | 287 required (Power 80%);  286 patients were recruited  (143 in each arm). |
| 445 | Takahashi, 2017(5) | Lancet Oncol | Phase III,  ED-SCLC:  PCI vs no PCI | NI | MRI | Brain MRI at 3-month intervals up to 12 months and at 18 and 24 months after enrolment | Yes | Secondary | 03-04-2009 ~  17-07-2013 | 330 required (power: 80%);  224 recruited:  PCI: 113; No PCI: 111 |
|  | 3) SCLC |  |  |  |  |  |  |  |  |  |
| 18 | Arriagada, 1995(6)  (PCI 85) | J Natl Cancer Inst | SCLC, CR after induction therapy:  PCI vs no PCI | CT?^A^ | CT?^A^ | CT at 6, 18, 30, and 48 months after random assignment | NI | Primary | 05-1985 ~  03-1993 | 150 each arm (power: 95%);  300 randomized (149 PCI, 151 control),  145 received PCI,  149 no PCI |
| 225 | Laplanche, 1998(7)  (PCI 88) | Lung Cancer | SCLC, CR after induction therapy:  PCI vs no PCI | NI | NI | CT was performed when indicated. | NI | Secondary | 10-1988 ~  04-1994 | 550 per group (power: 95%); 211 included (100 PCI, 111 no PCI ) (power: 37%) |
| 19 | Arriagada, 2002(8)  (PCI 85 + PCI 88) | Ann Oncol | SCLC, CR after induction therapy:  PCI vs no PCI | CT?^A^ | CT?^A^ | CT was performed when indicated. | NI | Primary | 05-1985 ~  04-1994 | NI for targeted size;  505 enrolled:  PCI85: 294 (145 PCI, 149 no PCI );  PCI88: 211 (100 PCI, 111 no PCI ) |
|  | 2. PCI dose |  |  |  |  |  |  |  |  |  |
| 231 | Le Pechoux, 2009(9) | Lancet Oncol | Phase III, LD-SCLC with CR after CRT:  PCI high dose (36Gy/24f/bid vs 36Gy/18f/qd)  vs standard dose (25Gy). | NI | 23% had MRI, 73% had CT | MRI/CT yearly or before in case of neurological symptoms | NI | Primary | 09-1999 ~  12-2005 | NI for targeted size;  720 (360 in each arm) enrolled |
|  | 3. TRT vs no TRT in ED-SCLC | |  |  |  |  |  |  |  |  |
| 526 | Slotman, 2015(10)  (CREST) | Lancet | Phase III,  ED-SCLC:  TRT vs no TRT | Brain CT/MRI was done for all patients with symptoms  suggestive of BM.  230 (46%) of  asymptomatic patients underwent a brain CT/  MRI | A brain CT/MRI was done for all patients with symptoms  suggestive of BM.  43 (13%) of  asymptomatic patients  underwent a brain CT/MRI | NI | NI | Secondary | 18-02-2009 ~  21-12-2012 | 483 required (power: 80%);  498 randomized (249 TRT, 249 no TRT), 495 analyzed (247 received TRT, 248 no TRT) |
| 140 | Gore, 2017(11) (RTOG 0937) | J Thorac Oncol | Phase II,  ED-SCLC:  TRT vs no TRT | No | Yes, MRI/CT | Brain imaging were required at 2, 6, 9, and 12 months; every 6 months for 2 to 3 years; and then annually. | NI | Secondary | 18-03-2010 ~  27-02-2015 | 154 required (power: 80%);  97 randomized (46 TRT, 51 no TRT), 86 eligible (44 received TRT, 42 no TRT) |
|  | 4. TRT timing |  |  |  |  |  |  |  |  |  |
| 488 | Work, 1997(12) | J Clin Oncol | LD-SCLC:  Early TRT (initial TRT) + PCI vs Late TRT (delayed 18 weeks) + PCI | Only performed  when indicated. | Only performed  when indicated. | Only performed  when indicated. | NI | NI | 03-1981 ~  09-1989 | 100-> 200;  199 were eligible, 157 were given PCI:  Early TRT: 99;  Late TRT: 58. |
| 532 | Jeremic, 1997(13) | J Clin Oncol | LD-SCLC: Early vs Late TDRT (week 1 vs week 6) | CT or radionuclide | CT or radionuclide | NI | NI | Secondary | 01-1988 ~  12-1992 | 170 required,  107 enrolled,  103 included:  Early: 52;  Late: 51. |
| 531 | Skarlos, 2001(14)  (HeCOG) | Ann Oncol | LD-SCLC:  Early vs Late TDRT (1^st^ vs 4^th^ chemo) | NI | NI | Brain CT:  During treatment: every 2 cycles of chemo; after treatment:  every 3 months for the first year, every 4 months for the second year and every 6  months thereafter | NI | Secondary | 12-1993 ~  11-1999 | 86 required,  81 included:  Early: 42;  Late: 39. |
| 429 | Spiro, 2006(15) | J Clin Oncol | LD-SCLC: Early vs Late TRT (2^nd^ vs 6^th^ chemo) | CT: 153/325=47%; | CT | Performed when indicated. | NI | Secondary | 1993 ~  1999 | 320 required (power: 80%);  325 recruited: Early TRT: 159; Late TRT: 166 |
|  | 5.CRT sequence |  |  |  |  |  |  |  |  |  |
| 530 | Gregor, 1997(16)  (EORTC08877) | J Clin Oncol | Phase III,  LD-SCLC:  Alternating vs SCRT | NI | NI | NI | NI | Secondary | 03-1989 ~  01-1995 | 360 required (Power: 80%); 349 recruited (175 in arm A, 174 in arm S), 14 were ineligible (5 in arm A, 9 in arm S). |
| 529 | Takada, 2002(17) (JCOG 9104) | J Clin Oncol | LD-SCLC;  SCRT vs CCRT | CT | NI | NI | NI | Secondary | 05-1991 ~  01-1995 | 220 required (Power 80%);  231 recruited, 228 eligible (114 in each arm). |
|  | 6.TRT fractionation | |  |  |  |  |  |  |  |  |
| 239 | Levy, 2019(18);  Faivre-Finn, 2017(19);  Faivre-Finn, 2016(20).  (CONVERT trial) | J Thorac Oncol;  Lancet Oncol;  BMJ Open | Phase III,  LD-SCLC:  TDRT vs ODRT | MRI/CT:  CT: 79% (356/449);  MRI: 18% (83/449) | No | No | NI | Secondary | 17-04-2008 ~  29-11-2013 | 532 required (Power 80%); 547 recruited (274 TDRT, 273 ODRT), 449 received PCI (229 TDRT, 220 ODRT). |
|  | 7. Topotecan vs observation in ED-SCLC | | |  |  |  |  |  |  |  |
| 388 | Schiller,2001(21)  (E7593) | J Clin Oncol | Phase III,  ED-SCLC:  EP -> Topotecan  vs EP -> Observation | No | No | No | NA | Secondary | 03-1995 ~  01-1999 | 284 patients for step 2 needed (Power 90%);  420 recruitment for step 1 required.  421 recruited (274 TDRT, 273 ODRT),  402 eligible.  242 randomized (122 Topotecan, 120 observation), 223 eligible (112 Topotecan, 111 observation) |
| 536 | Sundstrøm, 2002(22) | J Clin Oncol | Phase III,  SCLC:  EP vs CEV | No. Only performed  when indicated. | No. Only performed  when indicated. | No | NI | Secondary | 01-1989 ~  08-1994 | 436 randomized  (218 EP, 218 CEV) |
| ***Notes:***  ^A^: Not sure the brain image was before treatment or before PCI.  ***Abbreviations:***  CEV, cyclophosphamide-epirubicin-vincristine; CR, complete response; CRT, chemoradiotherapy; CT, Computerized Tomography; ED, extensive-stage disease; EP: Etoposide-platinum; LD, limited-stage disease; MRI, Magnetic Resonance Imaging; NI, no information; PCI, prophylactic cranial irradiation; SCLC, small cell lung cancer; TRT, thoracic radiotherapy. | | | | | | | | | | |

**References:**

1. Work E, Bentzen SM, Nielsen OS, Fode K, Michalski W, Palshof T. Prophylactic cranial irradiation in limited stage small cell lung cancer: survival benefit in patients with favourable characteristics. Eur J Cancer. 1996;32a(5):772-8. doi:10.1016/0959-8049(95)00597-8.

2. Gregor A, Cull A, Stephens RJ, Kirkpatrick JA, Yarnold JR, Girling DJ, et al. Prophylactic cranial irradiation is indicated following complete response to induction therapy in small cell lung cancer: results of a multicentre randomised trial. United Kingdom Coordinating Committee for Cancer Research (UKCCCR) and the European Organization for Research and Treatment of Cancer (EORTC). Eur J Cancer. 1997;33(11):1752-8. doi:10.1016/s0959-8049(97)00135-4.

3. Cao KJ, Huang HY, Tu MC, Pan GY. Long-term results of prophylactic cranial irradiation for limited-stage small-cell lung cancer in complete remission. Chin Med J (Engl). 2005;118(15):1258-62.

4. Slotman B, Faivre-Finn C, Kramer G, Rankin E, Snee M, Hatton M, et al. Prophylactic cranial irradiation in extensive small-cell lung cancer. The New England journal of medicine. 2007;357(7):664-72. doi:10.1056/NEJMoa071780.

5. Takahashi T, Yamanaka T, Seto T, Harada H, Nokihara H, Saka H, et al. Prophylactic cranial irradiation versus observation in patients with extensive-disease small-cell lung cancer: a multicentre, randomised, open-label, phase 3 trial. The Lancet Oncology. 2017;18(5):663-71. doi:10.1016/s1470-2045(17)30230-9.

6. Arriagada R, Le Chevalier T, Borie F, Rivière A, Chomy P, Monnet I, et al. Prophylactic cranial irradiation for patients with small-cell lung cancer in complete remission. J Natl Cancer Inst. 1995;87(3):183-90. doi:10.1093/jnci/87.3.183.

7. Laplanche A, Monnet I, Santos-Miranda JA, Bardet E, Le Péchoux C, Tarayre M, et al. Controlled clinical trial of prophylactic cranial irradiation for patients with small-cell lung cancer in complete remission. Lung cancer (Amsterdam, Netherlands). 1998;21(3):193-201. doi:10.1016/s0169-5002(98)00056-7.

8. Arriagada R, Le Chevalier T, Rivière A, Chomy P, Monnet I, Bardet E, et al. Patterns of failure after prophylactic cranial irradiation in small-cell lung cancer: analysis of 505 randomized patients. Annals of oncology : official journal of the European Society for Medical Oncology. 2002;13(5):748-54. doi:10.1093/annonc/mdf123.

9. Le Péchoux C, Dunant A, Senan S, Wolfson A, Quoix E, Faivre-Finn C, et al. Standard-dose versus higher-dose prophylactic cranial irradiation (PCI) in patients with limited-stage small-cell lung cancer in complete remission after chemotherapy and thoracic radiotherapy (PCI 99-01, EORTC 22003-08004, RTOG 0212, and IFCT 99-01): a randomised clinical trial. The Lancet Oncology. 2009;10(5):467-74. doi:10.1016/s1470-2045(09)70101-9.

10. Slotman BJ, van Tinteren H, Praag JO, Knegjens JL, El Sharouni SY, Hatton M, et al. Use of thoracic radiotherapy for extensive stage small-cell lung cancer: a phase 3 randomised controlled trial. Lancet. 2015;385(9962):36-42. doi:10.1016/s0140-6736(14)61085-0.

11. Gore EM, Hu C, Sun AY, Grimm DF, Ramalingam SS, Dunlap NE, et al. Randomized Phase II Study Comparing Prophylactic Cranial Irradiation Alone to Prophylactic Cranial Irradiation and Consolidative Extracranial Irradiation for Extensive-Disease Small Cell Lung Cancer (ED SCLC): NRG Oncology RTOG 0937. J Thorac Oncol. 2017;12(10):1561-70. doi:10.1016/j.jtho.2017.06.015.

12. Work E, Nielsen OS, Bentzen SM, Fode K, Palshof T. Randomized study of initial versus late chest irradiation combined with chemotherapy in limited-stage small-cell lung cancer. Aarhus Lung Cancer Group. Journal of clinical oncology : official journal of the American Society of Clinical Oncology. 1997;15(9):3030-7. doi:10.1200/jco.1997.15.9.3030.

13. Jeremic B, Shibamoto Y, Acimovic L, Milisavljevic S. Initial versus delayed accelerated hyperfractionated radiation therapy and concurrent chemotherapy in limited small-cell lung cancer: a randomized study. Journal of clinical oncology : official journal of the American Society of Clinical Oncology. 1997;15(3):893-900. doi:10.1200/jco.1997.15.3.893.

14. Skarlos DV, Samantas E, Briassoulis E, Panoussaki E, Pavlidis N, Kalofonos HP, et al. Randomized comparison of early versus late hyperfractionated thoracic irradiation concurrently with chemotherapy in limited disease small-cell lung cancer: a randomized phase II study of the Hellenic Cooperative Oncology Group (HeCOG). Annals of oncology : official journal of the European Society for Medical Oncology. 2001;12(9):1231-8. doi:10.1023/a:1012295131640.

15. Spiro SG, James LE, Rudd RM, Trask CW, Tobias JS, Snee M, et al. Early compared with late radiotherapy in combined modality treatment for limited disease small-cell lung cancer: a London Lung Cancer Group multicenter randomized clinical trial and meta-analysis. Journal of clinical oncology : official journal of the American Society of Clinical Oncology. 2006;24(24):3823-30. doi:10.1200/jco.2005.05.3181.

16. Gregor A, Drings P, Burghouts J, Postmus PE, Morgan D, Sahmoud T, et al. Randomized trial of alternating versus sequential radiotherapy/chemotherapy in limited-disease patients with small-cell lung cancer: a European Organization for Research and Treatment of Cancer Lung Cancer Cooperative Group Study. Journal of clinical oncology : official journal of the American Society of Clinical Oncology. 1997;15(8):2840-9. doi:10.1200/jco.1997.15.8.2840.

17. Takada M, Fukuoka M, Kawahara M, Sugiura T, Yokoyama A, Yokota S, et al. Phase III study of concurrent versus sequential thoracic radiotherapy in combination with cisplatin and etoposide for limited-stage small-cell lung cancer: results of the Japan Clinical Oncology Group Study 9104. Journal of clinical oncology : official journal of the American Society of Clinical Oncology. 2002;20(14):3054-60. doi:10.1200/jco.2002.12.071.

18. Levy A, Le Péchoux C, Mistry H, Martel-Lafay I, Bezjak A, Lerouge D, et al. Prophylactic Cranial Irradiation for Limited-Stage Small-Cell Lung Cancer Patients: Secondary Findings From the Prospective Randomized Phase 3 CONVERT Trial. J Thorac Oncol. 2019;14(2):294-7. doi:10.1016/j.jtho.2018.09.019.

19. Faivre-Finn C, Snee M, Ashcroft L, Appel W, Barlesi F, Bhatnagar A, et al. Concurrent once-daily versus twice-daily chemoradiotherapy in patients with limited-stage small-cell lung cancer (CONVERT): an open-label, phase 3, randomised, superiority trial. The Lancet Oncology. 2017;18(8):1116-25. doi:10.1016/s1470-2045(17)30318-2.

20. Faivre-Finn C, Falk S, Ashcroft L, Bewley M, Lorigan P, Wilson E, et al. Protocol for the CONVERT trial-Concurrent ONce-daily VErsus twice-daily RadioTherapy: an international 2-arm randomised controlled trial of concurrent chemoradiotherapy comparing twice-daily and once-daily radiotherapy schedules in patients with limited stage small cell lung cancer (LS-SCLC) and good performance status. BMJ open. 2016;6(1):e009849. doi:10.1136/bmjopen-2015-009849.

21. Schiller JH, Adak S, Cella D, DeVore RF, 3rd, Johnson DH. Topotecan versus observation after cisplatin plus etoposide in extensive-stage small-cell lung cancer: E7593--a phase III trial of the Eastern Cooperative Oncology Group. Journal of clinical oncology : official journal of the American Society of Clinical Oncology. 2001;19(8):2114-22. doi:10.1200/jco.2001.19.8.2114.

22. Sundstrøm S, Bremnes RM, Kaasa S, Aasebø U, Hatlevoll R, Dahle R, et al. Cisplatin and etoposide regimen is superior to cyclophosphamide, epirubicin, and vincristine regimen in small-cell lung cancer: results from a randomized phase III trial with 5 years' follow-up. Journal of clinical oncology : official journal of the American Society of Clinical Oncology. 2002;20(24):4665-72. doi:10.1200/jco.2002.12.111.
