# Supplementary material for: Risk Factors for Brain Metastases in Patients With Small Cell Lung Cancer: A Systematic Review and Meta-Analysis
Source: Front Oncol. 2022 Jun 10;12:889161. doi: 10.3389/fonc.2022.889161 (PMC9226404; doi:10.3389/fonc.2022.889161)
Supplement: Supplementary file 7 [file Table_5.docx]

Appendix Table 5. Baseline characteristics and treatments of the randomized clinical trials

| ID | First Author,  (Trial) | Median follow-up | Age | PS | Gender (Male percentage) | Surgery | TRT | Chemo | PCI |
| --- | --- | --- | --- | --- | --- | --- | --- | --- | --- |
|  | 1. PCI vs no PCI |  |  |  |  |  |  |  |  |
|  | 1) LD-SCLC |  |  |  |  |  |  |  |  |
| 487 | Work, 1996(1) | NI | Median (range): PCI: 61 (36-70),  No PCI: 59 (36-69),  Total: 60 (36-70). | KPS  100: 11.6%;  90-80: 69.3%;  70-60: 14.6%;  50-40: 4.5%. | PCI: 60%  No PCI: 71.4%  Total: 61.5% | No | Before October 1984: 40 Gy;  After October 1984: 45 Gy.. | Yes | PCI: 157  No PCI: 42 |
| 148 | Gregor, 1997(2)  (UKCCCR/EORTC) | 18 months | Median (range):  No PCI:61 (28-76),  PCI: 60 (37-79). | NI | No PCI: 74/120(62%), PCI: 125/194(64%). | NI | 84% TRT (263/314) | Yes | PCI: 194/314  No PCI: 120/314 |
| 62 | Cao, 2005(3) | >5 years | ≤65;  Mean±SD:  No PCI:55.63 ± 7.29,  PCI: 54.69 ± 7.56. | KPS ≥70 | No PCI: 92%, PCI: 92%. | NI | Yes, 56-70Gy. | Yes | PCI: 26 (51%);  No PCI: 25(49%) |
|  | 2) ED-SCLC |  |  |  |  |  |  |  |  |
| 415 | Slotman, 2007(4)  (EORTC) | NI | Median (range):  No PCI:63 (39-75), PCI: 62 (37-75). | 0:No PCI:52/143(36.4%),  PCI: 52/143(36.4%);  1:No PCI:76/143(53.1%),  PCI: 80/143(55.9%);  2:No PCI:15/143(10.5%),  PCI: 11/143(7.7%);  3:No PCI:0/177(0%),  PCI: 1/163(1%);  Unknown:No PCI:0/177(0%),  PCI: 1/163(1%).  (0:No PCI:105/177(59%),  PCI: 77/163(47%);  >0:No PCI:68/177(41%),  PCI: 86/163(53%) | No PCI: 82/143(57.3%), PCI: 97/143(67.8%). | NI | NI | Yes | PCI: 143/286  No PCI: 143/286 |
| 445 | Takahashi, 2017(5) | 11.9 months | Median (range):  No PCI:69 (37-86), PCI: 69 (43-83). | 0-1:No PCI:107/111(96%),  PCI: 108/113(96%);  2:No PCI:4/111(4%),  PCI: 5/113(4%). | No PCI: 98/111(88%), PCI: 95/113(84%). | NI | NI | Yes | PCI: 106/224  No PCI: 118/224 |
|  | 3) SCLC |  |  |  |  |  |  |  |  |
| 18 | Arriagada, 1995(6)  (PCI 85) | NI | Mean±SD:  No PCI:56 ± 9, PCI: 57 ± 8. | KPS  90-100:No PCI:62%,  PCI: 62%; 70-80: No PCI:35%,  PCI: 37%; ≤60: No PCI:3%, PCI: 1%. | No PCI: 86%, PCI: 88%. | 8% | 92% | 99% | PCI: 145 (49%);  No PCI: 149(51%) |
| 225 | Laplanche, 1998(7) (PCI 88) | 5 years | Mean±SD:  No PCI:57 ± 9,  PCI: 58 ± 8. | KPS  ≥90: No PCI:75%,  PCI: 82%;  <90: No PCI:25%,  PCI: 18%. | No PCI: 92%,  PCI: 89%. | NI | NI | Yes | PCI: 100(47%), no PCI: 111(53%) |
| 19 | Arriagada, 2002(8)  (PCI 85 + PCI 88) | 11 years | See above | KPS  >80: No PCI:68%,  PCI: 70%; 70-80: No PCI:31%,  PCI: 29%; ≤60: No PCI:2%, PCI: 1%. | No PCI: 88%, PCI: 88%. | NI | NI | Yes | PCI: 245(51%), no PCI: 260 (49%) |
|  | 2. PCI dose |  |  |  |  |  |  |  |  |
| 231 | Le Pechoux, 2009(9) | 39 months | Median (range):  standard dose: 60 (38-83), higher dose: 60 (34-78). | NI | Standard dose: 234/360 (65%);  higher dose: 226/360 (63%). | NI | Yes (685/720=95%) | Yes | Yes (711/720=99%) |
|  | 3. TRT vs no TRT in ED-SCLC | |  |  |  |  |  |  |  |
| 526 | Slotman, 2015(10)  (CREST) | 24 months | Median (IQR):  No TRT:63 (57-69);  TRT: 63 (58-69). | 0:No TRT:70/248 (28%),  TRT: 97/247 (39%);  1:No TRT: 155/248(63%),  TRT: 121/247(49%);  2:No TRT:23/248(9%),  TRT: 29/247(12%). | No TRT: 55%;  TRT: 55%; | No | Yes (30Gy/10f):  248 (50%);  No TRT: 247 (50%) | Yes | Yes |
| 140 | Gore, 2017(11) (RTOG 0937) | 9 months | Median (range):  No TRT:60.5 (47-81);  TRT: 66 (35-86);  Total: 63 (35-86). | 0: 39/86 (45.3%);  1: 46/86 (53.5%);  2: 1/86 (1.2%). | No TRT: 42.9%;  TRT: 47.7%;  Total: 45.3% | No | Yes (45Gy/15f):  44 (51%);  No TRT: 42 (49%) | Yes | Yes |
|  | 4. TRT timing |  |  |  |  |  |  |  |  |
| 488 | Work, 1997(12) | NI | Median (range):  Early TRT: 61 (36-70), Late TRT: 59 (36-69),  Total: 60 (36-70). | KPS  100: 11.6%;  90-80: 69.3%;  70-60:14.6%;  50-40: 4.5%. | Early TRT: 54.5%  Late TRT: 70.6%  Total: 61.5% | No | Before October 1984: 40 Gy;  After October 1984: 45 Gy.. | Yes | PCI: 157  No PCI: 42 |
| 532 | Jeremic, 1997(13) | NI | Median (range): Early TRT: 59 (40-67),  late TRT: 59 (44-66), | 90-100: 51/103 (49.5%),  50-80: 52/103 (50.5%) | Early TRT: 31/52 (59.6%),  Late TRT: 31/51(60.8%). | NI | 54Gy/36f, bid | Yes | PCI was given to patients achieving a complete response. |
| 531 | Skarlos, 2001(14)  (HeCOG) | 35 months | Median (range): Early TRT:  61 (40-76),  Late TRT:  60 (37.5-76), | 0:  Early: 11/42 (26%),  Late: 16/39 (41%).  1:  Early: 21/42 (50%),  Late: 17/39 (44%).  2:  Early: 10/42 (24%),  Late: 6/39 (15%). | Early TRT: 39/42 (93%),  Late TRT: 35/39 (90%). | NI | 45Gy/30f, bid | Yes | PCI was given to patients achieving a complete response. |
| 429 | Spiro, 2006(15) | 63 months | Median (range):  Early TRT:  62 (34-74),  Late TRT:  62 (33-74). | 0-1:  Early TRT:144/159 (91%),  Late TRT: 148/166 (89%).  2-3:  Early TRT:15/159 (9%),  Late TRT: 18/166 (11%). | Early TRT: 95/159 (60%),  Late TRT: 94/166 (57%). | NI | Yes: 40Gy/15f; | Yes | Responding to CRT: Yes: |
|  | 5.Treatment sequence: CCRT vs SCRT, Alternating vs SCRT: | | | |  |  |  |  |  |
| 530 | Gregor, 1997(16)  (EORTC08877) | 43 months | Median (range):  A:61 (34-74),  S: 61 (33-75). | 0:A:80/170 (47%),  S: 76/165 (46%);  1:A:76/170 (45%),  S: 79/165 (48%);  2:A:10/170 (6%),  S: 7/165 (4%);  3.A:3/170 (2%),  S: 3/165 (2%); | A:112/170 (66%),  S: 112/165 (68%); | No | Alternating:  50Gy/20f, 4 1-week courses.  SCRT:  50Gy/20f, 4 consecutive weeks. | Yes | No |
| 529 | Takada, 2002(17)  (JCOG 9104) | NI | Median (range): SCRT: 64 (30-74), CCRT: 65 (39-74), | 0:  SCRT: 33/114 (29%),  CCRT: 25/114 (22%).  1:  SCRT: 75/114 (66%),  CCRT: 83/114 (73%).  2:  SCRT: 6/114 (5%),  CCRT: 6/114 (5%). | SCRT: 93/114(82%), CCRT: 91/114(80%). | NI | 45Gy/30f, bid | Yes | PCI was administered to  patients with a complete or near-complete response |
|  | 6.TRT fractionation: ODRT vs TDRT | | |  |  |  |  |  |  |
| 239 | Levy, 2019(18) (CONVERT trial) | 45 months | Median (range):  62 (29-81) | 0: 215 (48%);  1: 222 (49%);  2: 12 (3%). | 252 (56%) | NI | Yes | Yes | Yes (449) |
|  | 7. Chemo: Topotecan vs observation in ED-SCLC, EP vs CEV in SCLC | | | |  |  |  |  |  |
| 388 | Schiller,2001(19)  (E7593) | 21 months | Median:  Topotecan: 62.5, Observation: 62. | 0: Topotecan: 29%,  Observation: 34%;  1: Topotecan: 60%,  Observation: 54%;  2: Topotecan: 12%,  Observation: 12%. | Topotecan: 64%,  Observation: 61%; | Topotecan: 37%;  observation: 22%; | NI | Yes | NI |
| 536 | Sundstrøm, 2002(20) | >5 years | Median (range):  EP: 64 (41-75);  CEV: 64 (39-76) | 0: EP: 22%,  CEV: 18%;  1: EP: 47%,  CEV: 44%;  2: EP: 28%,  CEV: 35%;  3: EP: 3%,  CEV: 3%. | EP: 66%,  CEV: 63%; | NI | LD-SCLC: Yes | Yes, EP vs CEV | PCI was administered to  LD-SCLC patients with a complete response:  EP: 20%;  CEV: 23%. |
| ***Abbreviations:***  CCRT, concurrent chemoradiotherapy; CEV, cyclophosphamide-epirubicin-vincristine; chemo, chemotherapy; CRT, chemoradiotherapy; ED-SCLC, extensive-stage disease small cell lung cancer; EP: Etoposide-platinum; IQR, Interquartile range; LD-SCLC, limited-stage disease small cell lung cancer; KPS, Karnofsky performance status scale; NI: no information; ODRT, once-daily radiotherapy; PCI, prophylactic cranial irradiation; PS, performance status; SCRT, sequential chemoradiotherapy; TDRT, twice-daily radiotherapy; TRT, thoracic radiotherapy. | | | | | | | | | |

**References:**

1. Work E, Bentzen SM, Nielsen OS, Fode K, Michalski W, Palshof T. Prophylactic cranial irradiation in limited stage small cell lung cancer: survival benefit in patients with favourable characteristics. Eur J Cancer. 1996;32a(5):772-8. doi:10.1016/0959-8049(95)00597-8.

2. Gregor A, Cull A, Stephens RJ, Kirkpatrick JA, Yarnold JR, Girling DJ, et al. Prophylactic cranial irradiation is indicated following complete response to induction therapy in small cell lung cancer: results of a multicentre randomised trial. United Kingdom Coordinating Committee for Cancer Research (UKCCCR) and the European Organization for Research and Treatment of Cancer (EORTC). Eur J Cancer. 1997;33(11):1752-8. doi:10.1016/s0959-8049(97)00135-4.

3. Cao KJ, Huang HY, Tu MC, Pan GY. Long-term results of prophylactic cranial irradiation for limited-stage small-cell lung cancer in complete remission. Chin Med J (Engl). 2005;118(15):1258-62.

4. Slotman B, Faivre-Finn C, Kramer G, Rankin E, Snee M, Hatton M, et al. Prophylactic cranial irradiation in extensive small-cell lung cancer. The New England journal of medicine. 2007;357(7):664-72. doi:10.1056/NEJMoa071780.

5. Takahashi T, Yamanaka T, Seto T, Harada H, Nokihara H, Saka H, et al. Prophylactic cranial irradiation versus observation in patients with extensive-disease small-cell lung cancer: a multicentre, randomised, open-label, phase 3 trial. The Lancet Oncology. 2017;18(5):663-71. doi:10.1016/s1470-2045(17)30230-9.

6. Arriagada R, Le Chevalier T, Borie F, Rivière A, Chomy P, Monnet I, et al. Prophylactic cranial irradiation for patients with small-cell lung cancer in complete remission. J Natl Cancer Inst. 1995;87(3):183-90. doi:10.1093/jnci/87.3.183.

7. Laplanche A, Monnet I, Santos-Miranda JA, Bardet E, Le Péchoux C, Tarayre M, et al. Controlled clinical trial of prophylactic cranial irradiation for patients with small-cell lung cancer in complete remission. Lung cancer (Amsterdam, Netherlands). 1998;21(3):193-201. doi:10.1016/s0169-5002(98)00056-7.

8. Arriagada R, Le Chevalier T, Rivière A, Chomy P, Monnet I, Bardet E, et al. Patterns of failure after prophylactic cranial irradiation in small-cell lung cancer: analysis of 505 randomized patients. Annals of oncology : official journal of the European Society for Medical Oncology. 2002;13(5):748-54. doi:10.1093/annonc/mdf123.

9. Le Péchoux C, Dunant A, Senan S, Wolfson A, Quoix E, Faivre-Finn C, et al. Standard-dose versus higher-dose prophylactic cranial irradiation (PCI) in patients with limited-stage small-cell lung cancer in complete remission after chemotherapy and thoracic radiotherapy (PCI 99-01, EORTC 22003-08004, RTOG 0212, and IFCT 99-01): a randomised clinical trial. The Lancet Oncology. 2009;10(5):467-74. doi:10.1016/s1470-2045(09)70101-9.

10. Slotman BJ, van Tinteren H, Praag JO, Knegjens JL, El Sharouni SY, Hatton M, et al. Use of thoracic radiotherapy for extensive stage small-cell lung cancer: a phase 3 randomised controlled trial. Lancet. 2015;385(9962):36-42. doi:10.1016/s0140-6736(14)61085-0.

11. Gore EM, Hu C, Sun AY, Grimm DF, Ramalingam SS, Dunlap NE, et al. Randomized Phase II Study Comparing Prophylactic Cranial Irradiation Alone to Prophylactic Cranial Irradiation and Consolidative Extracranial Irradiation for Extensive-Disease Small Cell Lung Cancer (ED SCLC): NRG Oncology RTOG 0937. J Thorac Oncol. 2017;12(10):1561-70. doi:10.1016/j.jtho.2017.06.015.

12. Work E, Nielsen OS, Bentzen SM, Fode K, Palshof T. Randomized study of initial versus late chest irradiation combined with chemotherapy in limited-stage small-cell lung cancer. Aarhus Lung Cancer Group. Journal of clinical oncology : official journal of the American Society of Clinical Oncology. 1997;15(9):3030-7. doi:10.1200/jco.1997.15.9.3030.

13. Jeremic B, Shibamoto Y, Acimovic L, Milisavljevic S. Initial versus delayed accelerated hyperfractionated radiation therapy and concurrent chemotherapy in limited small-cell lung cancer: a randomized study. Journal of clinical oncology : official journal of the American Society of Clinical Oncology. 1997;15(3):893-900. doi:10.1200/jco.1997.15.3.893.

14. Skarlos DV, Samantas E, Briassoulis E, Panoussaki E, Pavlidis N, Kalofonos HP, et al. Randomized comparison of early versus late hyperfractionated thoracic irradiation concurrently with chemotherapy in limited disease small-cell lung cancer: a randomized phase II study of the Hellenic Cooperative Oncology Group (HeCOG). Annals of oncology : official journal of the European Society for Medical Oncology. 2001;12(9):1231-8. doi:10.1023/a:1012295131640.

15. Spiro SG, James LE, Rudd RM, Trask CW, Tobias JS, Snee M, et al. Early compared with late radiotherapy in combined modality treatment for limited disease small-cell lung cancer: a London Lung Cancer Group multicenter randomized clinical trial and meta-analysis. Journal of clinical oncology : official journal of the American Society of Clinical Oncology. 2006;24(24):3823-30. doi:10.1200/jco.2005.05.3181.

16. Gregor A, Drings P, Burghouts J, Postmus PE, Morgan D, Sahmoud T, et al. Randomized trial of alternating versus sequential radiotherapy/chemotherapy in limited-disease patients with small-cell lung cancer: a European Organization for Research and Treatment of Cancer Lung Cancer Cooperative Group Study. Journal of clinical oncology : official journal of the American Society of Clinical Oncology. 1997;15(8):2840-9. doi:10.1200/jco.1997.15.8.2840.

17. Takada M, Fukuoka M, Kawahara M, Sugiura T, Yokoyama A, Yokota S, et al. Phase III study of concurrent versus sequential thoracic radiotherapy in combination with cisplatin and etoposide for limited-stage small-cell lung cancer: results of the Japan Clinical Oncology Group Study 9104. Journal of clinical oncology : official journal of the American Society of Clinical Oncology. 2002;20(14):3054-60. doi:10.1200/jco.2002.12.071.

18. Levy A, Le Péchoux C, Mistry H, Martel-Lafay I, Bezjak A, Lerouge D, et al. Prophylactic Cranial Irradiation for Limited-Stage Small-Cell Lung Cancer Patients: Secondary Findings From the Prospective Randomized Phase 3 CONVERT Trial. J Thorac Oncol. 2019;14(2):294-7. doi:10.1016/j.jtho.2018.09.019.

19. Schiller JH, Adak S, Cella D, DeVore RF, 3rd, Johnson DH. Topotecan versus observation after cisplatin plus etoposide in extensive-stage small-cell lung cancer: E7593--a phase III trial of the Eastern Cooperative Oncology Group. Journal of clinical oncology : official journal of the American Society of Clinical Oncology. 2001;19(8):2114-22. doi:10.1200/jco.2001.19.8.2114.

20. Sundstrøm S, Bremnes RM, Kaasa S, Aasebø U, Hatlevoll R, Dahle R, et al. Cisplatin and etoposide regimen is superior to cyclophosphamide, epirubicin, and vincristine regimen in small-cell lung cancer: results from a randomized phase III trial with 5 years' follow-up. Journal of clinical oncology : official journal of the American Society of Clinical Oncology. 2002;20(24):4665-72. doi:10.1200/jco.2002.12.111.
