# Supplementary material for: Risk Factors for Brain Metastases in Patients With Small Cell Lung Cancer: A Systematic Review and Meta-Analysis
Source: Front Oncol. 2022 Jun 10;12:889161. doi: 10.3389/fonc.2022.889161 (PMC9226404; doi:10.3389/fonc.2022.889161)
Supplement: Supplementary file 8 [file Table_6.docx]

Appendix table 6. Characteristics and treatments of non-randomized clinical trials

| ID | (Trial) | Journal | Study design | Reviewed period | Sample size | Median  follow-up | Age | PS | Gender (Male%) | Surgery | TRT | Chemo | PCI or not |
| --- | --- | --- | --- | --- | --- | --- | --- | --- | --- | --- | --- | --- | --- |
| Retrospective studies | | | | | | | | | | | | | |
| 28 | Bang, 2018(1) | Int J Radiat Oncol Biol Phys | ED-SCLC, without baseline BM, at least PR to chemo:  PCI vs  no PCI | 01-01-2005 ~  31-12-2011 | 397 screened  155 eligible | NI | 66 (43-89); | 0: 13/155 (8.4%); 1: 86/155 (55.5%); 2: 31/155 (20%); 3: 23/155 (14.8%); 4: 2/155 (1.3%); | 57.4% | NI | PCI: 62%; No PCI: 55%; (P=0.40) | Yes | PCI: 68  No PCI: 87 |
| 34 | Bernhardt, 2017(2) | Clin Lung Cancer | ED-SCLC, PCI | 2007 ~ 2015 | 136 | NI | 62 (45-86); | KPS:  80 (50-100) | 85/136 (62.5%) | NI | NI | Yes | Yes |
| 52 | Brewster, 1995(3) | Radiother Oncol | LD-SCLC,  PCI 8Gy | 07.1986 - 03.1989. | 106; patients with CR:73. | ≥ 24 months | 58 | NI | 59/106 (55.7%) | NI | 12.5Gy, 1f | Yes | Yes |
| 80 | Chen, 2016(4) | Strahlenther Onkol | ED-SCLC, without baseline BM, at least PR to chemo:  PCI vs no PCI | 04.2005 - 05.2014 | 204 | 11.2 months (range 2.9–71.7 months). | 58 (IQR 52–63). | 0: 71/204 (34.8%); 1: 124/204 (60.8%); 2: 9/204 (4.4%); | 171/204 (83.8%) | NI | NI | Yes | Yes: 45/204 (22.1%) |
| 81 | Chen, 2018(5) | Strahlenther Onkol | ED-SCLC:  Early vs late PCI | 11.2011 - 07.2016 | 103 | 12 months (range: 3–36 months). | 59 (IQR: 53–65) | 0: 37/103 (35.9%); 1: 61/103 (59.2%); 2: 5/103 (4.9%); | 89/103 | NI | Yes | Yes | Yes: 59/103 (57.3%) |
| 82 | Choi, 2017(6) | Clin Lung Cancer | LD-SCLC with PET-CT or not:  PCI vs no PCI | 04.2001 ~ 04.2013 | 1423 screened,  280 eligible | NI | <60: 103/280 (36.8%); 60-70: 129/280 (46.1%); ≥70 years: 48/280 (17.1%). | 0: 54/280 (19.3%); 1: 213/280 (76.1%); 2: 13/280 (4.6%). | 247/280 (88.2%) | NI | Yes | Yes | PCI: 90/280 ( %); No PCI: 190/280( %) |
| 86 | Chu, 2019(7) | Radiat Oncol | risk factors for pre-PCI BM | 2011-2017 | 283 screened, 110 eligible | For OS: 22.7 months (range 6.4– 92.0months); For BM: 5.2 months (range 2.8–9.2 months). | 38-79 | NI | 95/110 (86.4%) | NI | Yes: CCRT: 93/110; SCRT: 17/110 | Yes | No |
| 108 | El Sharouni, 2009(8) | Anticancer Res | LD-SCLC: SCRT vs CCRT; | 1996-2005 | 151 eligible,  16 lost to follow-up:  SCRT: 95; CCRT:40.  SCRT+PCI: 67; CCRT+PCI: 23 | NI | 32-81.7 | NI | 94/151 (62.3%) | NI | Yes | Yes | Yes |
| 112 | Eze, 2017(9) | Clin Lung Cancer | LD-SCLC with MRI:  PCI vs no PCI | 1998 ~ 2012 | 184 eligible | NI | 63 (34-83). | median: 1 (range, 0-3). | 111/184 (60%) | NI | Yes | Yes | PCI: 71/184 (39%); No PCI: 113/184 (61%) |
| 115 | Farooqi, 2017(10) | Radiother Oncol | LS-SCLC:  PCI vs no PCI | 1986 ~ 2012 | 658  (PCI: 364; No PCI: 294) | 21.2 months (range 1.2–240.8 months) | 62 (27–95); ≥70 years: 151/668 (22.9%). | KPS: ≥ 80: 550/658 (83.6%); < 80: 108/658 (16.4%) | 342/658 (52.0%) | No | Yes | Yes | PCI: 364 (55.3%);  No PCI: 294 (44.7%) |
| 134 | Giuliani, 2010(11) | Cancer | LD-SCLC:  PCI vs no PCI | 1997 ~ 2007 | 796 screened, 207 analyzed | 18.8 months (range, 2.2-130.1 months). | 65.7  (38.6- 88.1). | NI | 111/207 (53.6%) | NI | 4005 cGy / 15 fractions | Yes | PCI: 127/207(61.4%) No PCI: 80/207(38.6%) |
| 139 | Gong, 2013(12) | Int J Radiat Oncol Biol Phys | SCLC with surgery | 01.1998 - 12.2009 | 126 eligible | NI | 59 (28–79). | NI | 91/126 (72.2%) | Yes | Yes: 50/126 (39.7%) | Induction: 51/126 (40.5%); adjuvant: 112/126 (88.9%) | No |
| 145 | Greenspoon, 2011(13) | J Thorac Oncol | risk factors for BM in ED-SCLC | 01.01.2004 - 31.12.2006 | 130,  101 without baseline BM | NI | NI | 0: 3/130 (2.3%);  1: 49/130 (37.7%); 2: 48/130 (36.9%);  3: 21/130 (16.2%);  4: 9/130 (6.9%). | 66/130 (50.8%) | Yes: 111/130 (85.4%) | NI | NI | No |
| 203 | Kim, 2019(14) | J Radiat Res | LD-SCLC:  PCI vs no PCI | 11.1994 ~ 06.2010 | 320 screened,  235 analyzed | 22 months (range 1–150 months). | 61 (34–77). | 0: 29; 1-2: 205 | 204/234 (87%) | NI | Yes | Yes | PCI: 139/234 (59.4%); No PCI: 95/234 (40.6%) |
| 263 (264) | Manapov, 2012(15)  (Manapov, 2012(16)) | Strahlenther Onkol  ( J Neurooncol) | LD-SCLC, PS 2-3 | 1997-2008 | 149, 125 completed CRT: SCRT: 51/125 (41%); CCRT:74/125(59%) | NI | Median: 63.2 | 2 (2-3) | 78/125(62%) | NI | Yes | Yes | CCRT: 16 (31%);  SCRT: 20 (27%). |
| 265 | Manapov, 2013(17) | Tumori | LD-SCLC | 1998-2007 | 125: SCRT: 51/125 (41%); CCRT:74/125(59%) | 448 days (range, 35-3432). | Median: 63 | 2 (1-3) | 78/125(62%) | NI | Yes | Yes | 36/125 (29%) patients who achieved a CR. |
| 303 | Nakamura, 2018(18) | J Radiat Res | LD-SCLC:  PCI vs no PCI | 01.2006~ 12.2014 | 162 analyzed | 38 months (range, 6–105 months). | 67.5 (23–85) | 0: 71 (44%); 1-2: 91 (56%) | 130 (80%) | NI | Yes (45Gy/bid, or 50Gy/qd) | Yes | PCI: 93/162 (57%); No PCI: 69/162 (43%) |
| 312 | Nicholls, 2016(19) | Asia Pac J Clin Oncol | SCLC:  PCI vs no PCI | 01.2008 - 12.2013 | 203 | 7.6 months (range 0.5–76.5) | 65.4 (±10.7) | 0: 50/203 (24.6%); 1: 91/203 (44.8%); 2: 34/203(16.7%); 3: 19/203 (9.4%); 4: 3/203(1.5%); | 160/203 (79%) | NI | LD: Yes: 55/74;  ED: Yes: 63/129; | LD: Yes: 55/74;  ED: Yes: 101/129; | LD: 32/74 (43.2%); ED: 17/129 (13.1%) |
| 342 | Pezzi, 2020(20) | JAMA Netw Open | LD-SCLC with MRI:  PCI vs no PCI | 1992 ~ 2012 | 297 eligible,  168 matched | PCI: 83.64 months;  no-PCI group: 83.97 months | Before matching:  PCI: 62.2 years (range, 27.0-85.0 years); No PCI: 68.6 years (range, 40.0-86.0 years).  After matching:  PCI: 65.0 (44.0-85.0) No PCI: 67.5 (40.0-86.0) | before matching:  0: 72/297 (24.2%); 1: 189 /297 (63.6%); 2: 30/297 (10.1%); 3: 6/ 297 (2.0%).  after matching:  0: 38/168 (22.6%); 1: 105 /168 (62.5%); 2: 21/168 (12.5%); 3: 4/ 168 (2.4%). | before matching: 162/297 (54.5%); after matching: 96/168 (57.1%) | NI | Yes | Yes | before matching: PCI: 205/297(69%); No PCI: 92/297(31%);  after matching: PCI: 84/168 (50%) No PCI: 84/168 (50%) |
| 356 | Ramlov, 2012(21) | Lung Cancer | SCLC with PCI | 01.2007 ~ 08.2010 | 118 | All patients: 16.6 months (range 3–54 months); alive patients: 33 months (20–54 months) | 65 (46–80). | NI | 51/118 (43.2%) | Yes: 7/118 (6%) | Yes: 65/118 (53.4%) | Yes: 111/118 | Yes |
| 368 | Roengvoraphoj, 2017(22) | Strahlenther Onkol | LD-SCLC with CRT:  Male vs female | 1998 - 2012 | 179 | NI | 63 (range, 35–83) | 1 (range, 0–3) | 110/179 (61.5 %) | NI | Yes | Yes | Yes: 70/179 (39%) |
| 371 | Rubenstein, 1995(23) | Int J Radiat Oncol Biol Phys | LD-SCLC:  PCI vs no PCI | 06.1986 ~  12.1992 | 197 analyzed | mean:19 months, median:11.5 months (range, 1.1 - 89.8 months) | mean: 66 (range, 33 - 86) | Baseline KPS:  ≤80: 79 (41.8%); >80: 110 (58.2%). Pre-RT KPS: ≤80: 70 (37.0%); >80: 119 (63.0%). | NI | 10 (5.1%) | Yes: 195/197 (99%) | Yes | PCI: 112/197 (56.9%); No PCI: 69/197 (43.1%) |
| 376 | Sahmoun, 2004(24) | Anticancer Res | SCLC without PCI: Risk factors for BM in SCLC:  HT vs no HT | 06.1986 - 06.2003 | 232 screened, 185 eligible | NI | 67 (44-89); | NI | 130/185 (70%) | NI | NI | NI | No |
| 377 | Sahmoun, 2005(25) | Anticancer Res | Risk factors for BM and OS in SCLC:  Site, gender | 01.1989 - 12.2002 | 230 eligible, 209 without baseline BM | NI | 67 (41-89); | NI | 148/230 (64%) | NI | Yes: 134/230 | Yes: 182/230 | Yes: 12/209 (5.7%) |
| 384 | Sas-Korczyńska, 2010(26) | Strahlenther Onkol | LD-SCLC:  Early PCI vs Late PCI | 1995 - 2004 | 129 | 19 months (range: 4-135 months) | 57 (33-73) (Mean: 56.02) | 80 (60-90) | 83/129 (64.3%) | NI | Yes, CCRT | Yes | Yes: 86/129, 66.7%; (Early: 41/86. 47.7%; late: 45/86, 52.3%) |
| 393 | Scotti, 2014(27) | Tumori | TRT timing in LD-SCLC | 06.2000 - 05.2010 | 124:  CCRT: 53/124 (42.8%); SCRT: 71/124 (57.2%) | 2.2 years (range, 0.2-12.4) | ≤ 55: 34/124 (27.4%); 56-65: 42/124 (33.9%); ≥ 66: 48/124 (38.7%) | 0: 95/124 (76.6%); 1: 29/124 (23.4%) | 101/124 (81.5%) | NI | Yes | Yes | Yes: 38/124 (25.9%) |
| 439 | Suzuki, 2018(28) | Radiother Oncol | Risk factors for BM in SCLC:  Hematologic variables | 2001–2015 | 293 | 14.3 months  (IQR: 9.3–22.8 months) | 64 years (IQR, 58–71 years) | 0–1: 239/293 (82%) | 48% | No | ≥ 45 Gy: 200/293 (68%) | yes | Yes: 125/293 (43%) |
| 441 | Tai, 2013(29) | Clin Lung Cancer | LD-SCLC:  PCI vs  no PCI | 1981 ~ 2007 | 289 analyzed | NI | 65 years (range, 38-86 years) | NI | 168/289 (58.1%) | NI | Yes | Yes | PCI: 177/289 (61.2%); |
| 461 | van der Linden, 2001(30) | Respir Med | LD-SCLC with CR:  PCI vs  no PCI | 01.1985 ~ 10.1994 | 135 screened,  102 analyzed | 17 months (range 7 - 117 months) | NI | NI | NI | 9/135 | yes: 67/102 (65.7%): PCI: 55/65; No PCI: 12/37. | Yes | PCI: 65/102; No PCI: ˆ37/102. |
| 491 | Wu, 2017(31) | Radiother Oncol | LD-SCLC,  TNM vs BM | 1993-2013 | 333 screened, 283 eligible | 21.4 months | NI | KPS: ≥ 80: 241/283 (85.2%);  < 80: 42/283 (14.8%) | 127/283 (44.9%) | Yes: 69/283 (24.4%) | Yes: 236/283 (83.4%) | Yes: 264/283 (93.3%) | Yes: 116/283 (41.0%) |
| 493 | Xu, 2017(32) | J Thorac Oncol | Resected SCLC:  PCI vs  no PCI | 01.2006 ~ 01.2014 | 438 screened,  349 eligible | NI | median: NI (range, 38–79); <60 years: 155/349 (44.4%) ≥60 years: 194/349 (55.6%). | NI | 337/389 (85.1%) | Yes, all | yes: 229 (65.6%); median: 52 Gy (range 30–80 Gy). | Yes: 321 (92%); No: 28 (8.0%) | PCI: 115/349; No PCI: 234/349 |
| 513 | Zeng, 2019(33) | JAMA Netw Open | SCLC, with TRT, chemo, and PCI:  TDRT vs ODRT | 01-07-2003 ~  30-6-2016 | 894 screened,  778 eligible,  338 matched (1:1 PSM) | 23.6 months (interquartile range, 14.2-38.2 months), | 55 (interquartile range, 48-61), | 0: 127/778(16.3%); 1: 624/778(80.2%); 2: 27/778 (3.5%) | 574/778 (73.8%) | Yes: 44 (5.7%) | ODRT: 609/778 (78.3%); TDRT: 169/778 (21.7%) | Yes | Yes |
| 514 | Zeng, 2017(34) | Sci Rep | SCLC with PCI:  Risk factors for BM | 2003 ~  2014 | 204 screened, 175 eligible | 42.1 months (range, 7.4–119.4) | 55 (29-76) | 0: 10/175(5.7%); 1: 162/175(92.6%); 2: 3/175(1.7%) | 129/175 (73.7%) | NI | ODRT: 123/175; TDRT: 46/175 | Yes | Yes |
| 519 | Zheng, 2018(35) | Strahlenther Onkol | LD-SCLC without PCI:  Risk factors for BM | 01.2010 ~ 12.2016 | 153 | 42.5 months (range, 5.8–93.2 months). | 59 (23–80); | 0: 64/153 (41.8%); 1: 75/153 (49.0%); 2: 6/153 (3.9%) | 104/153 (68%) | NI | ODRT (mean: 56.9Gy, range:50–66Gy): 120; TDRT (45Gy/ bid): 19 | Yes | No |
| 520 | Zhu, 2014(36) | Radiat Oncol | Resected SCLC:  Risk factors for BM | 01.2003 ~ 12.2009 | 211 screened,  126 eligible | 56.0 months (range, 30.4–96.8 months). | 55 (34–74); <65 years: 91/126 (72.2%); ≥65 years: 35/126 (27.8%). | KPS: ≥ 80: 80/126 (64.0%);   < 80: 46/126 (36.0%) | 101/126 (80.2%) | Yes, all | yes:55/126; 50–60 Gy | yes | No |
| 521 | Zhu, 2014(37) | Lung Cancer | Resected SCLC:  PCI vs no PCI | 01.2003 ~ 12.2009 | 211 screened,  193 eligible | All: 39.4 months (range,4.0–96.8 months); surviving patients: 52.7 months (range,30.4–96.8 months). | 56 (34-78); <65 years: 143/193 (74.1%); ≥65 years: 50/193 (25.9%). | KPS:  ≥ 80: 126/193 (65.3%);  < 80: 67/193 (34.7%) | 150/193 (77.7%) | Yes, all | Yes: 94/193; 50–60 Gy | Yes | PCI: 67/193; No PCI: 126/193 |
|  | Prospective observation studies | | |  |  |  |  |  |  |  |  |  |  |
| 397 | Seute, 2004(38) | Cancer | SCLC,  Observe neurologic disorders | 10.1980 -  09. 2001 | 432 (11 were diagnosed SCLC with BM at autopsy) | NI | 66 (32–89) | NI | 347/432 (80.3%) | NI | occasionally applied | yes | 45/432 (10.4%) |
| 122 | Fu, 2014(39) | Jpn J Clin Oncol | SCLC with PCI | 11.2006 -02.2010 | 129 enrolled, 112 eligible | 25 months (5–66 months) | 58.5 years (IQR 49–69 years) | NI | NI | NI | Yes | Yes | Yes |
| *Abbreviations:* BM, brain metastasis; CCRT, concurrent chemoradiotherapy; chemo, chemotherapy; CR, complete response; CRT, chemoradiotherapy; ED-SCLC, extensive-stage disease small cell lung cancer; IQR, interquartile range; HT: Hypertension; KPS, Karnofsky performance status scale; LD-SCLC, limited-stage disease small cell lung cancer; MRI, magnetic resonance imaging; NI, no information; ODRT, once-daily radiotherapy; OS, overall survival; PCI, prophylactic cranial irradiation; PET-CT, positron emission tomography and computed tomography; PR, partial response; PS, performance status; SCLC, small cell lung cancer; SCRT, sequential chemoradiotherapy; TDRT, twice-daily radiotherapy; TRT, thoracic radiotherapy. | | | | | | | | | | | | | |

**References:**

1. Bang A, Kendal WS, Laurie SA, Cook G, MacRae RM. Prophylactic Cranial Irradiation in Extensive Stage Small Cell Lung Cancer: Outcomes at a Comprehensive Cancer Centre. International journal of radiation oncology, biology, physics. 2018;101(5):1133-40. doi:10.1016/j.ijrobp.2018.04.058.

2. Bernhardt D, Adeberg S, Bozorgmehr F, Opfermann N, Hoerner-Rieber J, Repka MC, et al. Nine-year Experience: Prophylactic Cranial Irradiation in Extensive Disease Small-cell Lung Cancer. Clin Lung Cancer. 2017;18(4):e267-e71. doi:10.1016/j.cllc.2016.11.012.

3. Brewster AE, Hopwood P, Stout R, Burt PA, Thatcher N. Single fraction prophylactic cranial irradiation for small cell carcinoma of the lung. Radiother Oncol. 1995;34(2):132-6. doi:10.1016/0167-8140(95)01513-g.

4. Chen Y, Li J, Hu Y, Zhang Y, Lin Z, Zhao Z, et al. Prophylactic cranial irradiation could improve overall survival in patients with extensive small cell lung cancer : A retrospective study. Strahlentherapie und Onkologie : Organ der Deutschen Rontgengesellschaft [et al]. 2016;192(12):905-12. doi:10.1007/s00066-016-1038-0.

5. Chen Y, Li J, Zhang Y, Hu Y, Zhang G, Yan X, et al. Early versus late prophylactic cranial irradiation in patients with extensive small cell lung cancer. Strahlentherapie und Onkologie : Organ der Deutschen Rontgengesellschaft [et al]. 2018;194(10):876-85. doi:10.1007/s00066-018-1307-1.

6. Choi M, Lee Y, Moon SH, Han JY, Kim HT, Lee JS. Effect of Accurate Staging Using Positron Emission Tomography on the Outcomes of Prophylactic Cranial Irradiation in Patients With Limited Stage Small-Cell Lung Cancer. Clin Lung Cancer. 2017;18(1):77-84. doi:10.1016/j.cllc.2016.06.012.

7. Chu X, Li S, Xia B, Chu L, Yang X, Ni J, et al. Patterns of brain metastasis immediately before prophylactic cranial irradiation (PCI): implications for PCI optimization in limited-stage small cell lung cancer. Radiation oncology (London, England). 2019;14(1):171. doi:10.1186/s13014-019-1371-4.

8. El Sharouni SY, Kal HB, Barten-Van Rijbroek A, Struikmans H, Battermann JJ, Schramel FM. Concurrent versus sequential chemotherapy and radiotherapy in limited disease small cell lung cancer: a retrospective comparative study. Anticancer Res. 2009;29(12):5219-24.

9. Eze C, Roengvoraphoj O, Niyazi M, Hildebrandt G, Fietkau R, Belka C, et al. Treatment Response and Prophylactic Cranial Irradiation Are Prognostic Factors in a Real-life Limited-disease Small-cell Lung Cancer Patient Cohort Comprehensively Staged With Cranial Magnetic Resonance Imaging. Clin Lung Cancer. 2017;18(4):e243-e9. doi:10.1016/j.cllc.2016.11.005.

10. Farooqi AS, Holliday EB, Allen PK, Wei X, Cox JD, Komaki R. Prophylactic cranial irradiation after definitive chemoradiotherapy for limited-stage small cell lung cancer: Do all patients benefit? Radiother Oncol. 2017;122(2):307-12. doi:10.1016/j.radonc.2016.11.012.

11. Giuliani M, Sun A, Bezjak A, Ma C, Le LW, Brade A, et al. Utilization of prophylactic cranial irradiation in patients with limited stage small cell lung carcinoma. Cancer. 2010;116(24):5694-9. doi:10.1002/cncr.25341.

12. Gong L, Wang QI, Zhao L, Yuan Z, Li R, Wang P. Factors affecting the risk of brain metastasis in small cell lung cancer with surgery: is prophylactic cranial irradiation necessary for stage I-III disease? International journal of radiation oncology, biology, physics. 2013;85(1):196-200. doi:10.1016/j.ijrobp.2012.03.038.

13. Greenspoon JN, Evans WK, Cai W, Wright JR. Selecting patients with extensive-stage small cell lung cancer for prophylactic cranial irradiation by predicting brain metastases. J Thorac Oncol. 2011;6(4):808-12. doi:10.1097/JTO.0b013e31820d782d.

14. Kim TG, Pyo H, Ahn YC, Noh JM, Oh D. Role of prophylactic cranial irradiation for elderly patients with limited-disease small-cell lung cancer: inverse probability of treatment weighting using propensity score. J Radiat Res. 2019;60(5):630-8. doi:10.1093/jrr/rrz040.

15. Manapov F, Klöcking S, Niyazi M, Belka C, Hildebrandt G, Fietkau R, et al. Chemoradiotherapy duration correlates with overall survival in limited disease SCLC patients with poor initial performance status who successfully completed multimodality treatment. Strahlentherapie und Onkologie : Organ der Deutschen Rontgengesellschaft [et al]. 2012;188(1):29-34. doi:10.1007/s00066-011-0016-9.

16. Manapov F, Klöcking S, Niyazi M, Levitskiy V, Belka C, Hildebrandt G, et al. Primary tumor response to chemoradiotherapy in limited-disease small-cell lung cancer correlates with duration of brain-metastasis free survival. J Neurooncol. 2012;109(2):309-14. doi:10.1007/s11060-012-0894-4.

17. Manapov F, Klöcking S, Niyazi M, Oskan F, Niemöller OM, Belka C, et al. Timing of failure in limited disease (stage I-III) small-cell lung cancer patients treated with chemoradiotherapy: a retrospective analysis. Tumori. 2013;99(6):656-60. doi:10.1700/1390.15452.

18. Nakamura M, Onozawa M, Motegi A, Hojo H, Zenda S, Nakamura N, et al. Impact of prophylactic cranial irradiation on pattern of brain metastases as a first recurrence site for limited-disease small-cell lung cancer. J Radiat Res. 2018;59(6):767-73. doi:10.1093/jrr/rry066.

19. Nicholls L, Keir GJ, Murphy MA, Mai T, Lehman M. Prophylactic cranial irradiation in small cell lung cancer: A single institution experience. Asia Pac J Clin Oncol. 2016;12(4):415-20. doi:10.1111/ajco.12564.

20. Pezzi TA, Fang P, Gjyshi O, Feng L, Liu S, Komaki R, et al. Rates of Overall Survival and Intracranial Control in the Magnetic Resonance Imaging Era for Patients With Limited-Stage Small Cell Lung Cancer With and Without Prophylactic Cranial Irradiation. JAMA network open. 2020;3(4):e201929. doi:10.1001/jamanetworkopen.2020.1929.

21. Ramlov A, Tietze A, Khalil AA, Knap MM. Prophylactic cranial irradiation in patients with small cell lung cancer. A retrospective study of recurrence, survival and morbidity. Lung cancer (Amsterdam, Netherlands). 2012;77(3):561-6. doi:10.1016/j.lungcan.2012.05.101.

22. Roengvoraphoj O, Eze C, Niyazi M, Li M, Hildebrandt G, Fietkau R, et al. Prognostic role of patient gender in limited-disease small-cell lung cancer treated with chemoradiotherapy. Strahlentherapie und Onkologie : Organ der Deutschen Rontgengesellschaft [et al]. 2017;193(2):150-5. doi:10.1007/s00066-016-1073-x.

23. Rubenstein JH, Dosoretz DE, Katin MJ, Blitzer PH, Salenius SA, Floody PA, et al. Low doses of prophylactic cranial irradiation effective in limited stage small cell carcinoma of the lung. International journal of radiation oncology, biology, physics. 1995;33(2):329-37. doi:10.1016/0360-3016(95)00166-v.

24. Sahmoun AE, Case LD, Chavour S, Kareem S, Schwartz GG. Hypertension and risk of brain metastasis from small cell lung cancer: a retrospective follow-up study. Anticancer Res. 2004;24(5b):3115-20.

25. Sahmoun AE, Case LD, Santoro TJ, Schwartz GG. Anatomical distribution of small cell lung cancer: effects of lobe and gender on brain metastasis and survival. Anticancer Res. 2005;25(2a):1101-8.

26. Sas-Korczyńska B, Korzeniowski S, Wójcik E. Comparison of the effectiveness of "late" and "early" prophylactic cranial irradiation in patients with limited-stage small cell lung cancer. Strahlentherapie und Onkologie : Organ der Deutschen Rontgengesellschaft [et al]. 2010;186(6):315-9. doi:10.1007/s00066-010-2088-3.

27. Scotti V, Meattini I, Franzese C, Saieva C, Bertocci S, Meacci F, et al. Radiotherapy timing in the treatment of limited-stage small cell lung cancer: the impact of thoracic and brain irradiation on survival. Tumori. 2014;100(3):289-95. doi:10.1700/1578.17206.

28. Suzuki R, Wei X, Allen PK, Welsh JW, Komaki R, Lin SH. Hematologic variables associated with brain failure in patients with small-cell lung cancer. Radiother Oncol. 2018;128(3):505-12. doi:10.1016/j.radonc.2018.05.026.

29. Tai P, Assouline A, Joseph K, Stitt L, Yu E. Prophylactic cranial irradiation for patients with limited-stage small-cell lung cancer with response to chemoradiation. Clin Lung Cancer. 2013;14(1):40-4. doi:10.1016/j.cllc.2012.04.005.

30. van der Linden YM, van Kempen ML, van der Tweel I, Vanderschueren RG, Schlösser NJ, Lammers JW, et al. Prophylactic cranial irradiation in limited disease small-cell lung cancer in complete remission: a retrospective analysis. Respir Med. 2001;95(3):235-6. doi:10.1053/rmed.2000.1022.

31. Wu AJ, Gillis A, Foster A, Woo K, Zhang Z, Gelblum DY, et al. Patterns of failure in limited-stage small cell lung cancer: Implications of TNM stage for prophylactic cranial irradiation. Radiother Oncol. 2017;125(1):130-5. doi:10.1016/j.radonc.2017.07.019.

32. Xu J, Yang H, Fu X, Jin B, Lou Y, Zhang Y, et al. Prophylactic Cranial Irradiation for Patients with Surgically Resected Small Cell Lung Cancer. J Thorac Oncol. 2017;12(2):347-53. doi:10.1016/j.jtho.2016.09.133.

33. Zeng H, Li R, Hu C, Qiu G, Ge H, Yu H, et al. Association of Twice-Daily Radiotherapy With Subsequent Brain Metastases in Adults With Small Cell Lung Cancer. JAMA network open. 2019;2(5):e190103. doi:10.1001/jamanetworkopen.2019.0103.

34. Zeng H, Xie P, Meng X, Yuan S, Sun X, Li W, et al. Risk factors for brain metastases after prophylactic cranial irradiation in small cell lung cancer. Scientific reports. 2017;7:42743. doi:10.1038/srep42743.

35. Zheng Y, Wang L, Zhao W, Dou Y, Lv W, Yang H, et al. Risk factors for brain metastasis in patients with small cell lung cancer without prophylactic cranial irradiation. Strahlentherapie und Onkologie : Organ der Deutschen Rontgengesellschaft [et al]. 2018;194(12):1152-62. doi:10.1007/s00066-018-1362-7.

36. Zhu H, Bi Y, Han A, Luo J, Li M, Shi F, et al. Risk factors for brain metastases in completely resected small cell lung cancer: a retrospective study to identify patients most likely to benefit from prophylactic cranial irradiation. Radiation oncology (London, England). 2014;9:216. doi:10.1186/1748-717x-9-216.

37. Zhu H, Guo H, Shi F, Zhu K, Luo J, Liu X, et al. Prophylactic cranial irradiation improved the overall survival of patients with surgically resected small cell lung cancer, but not for stage I disease. Lung cancer (Amsterdam, Netherlands). 2014;86(3):334-8. doi:10.1016/j.lungcan.2014.09.019.

38. Seute T, Leffers P, ten Velde GP, Twijnstra A. Neurologic disorders in 432 consecutive patients with small cell lung carcinoma. Cancer. 2004;100(4):801-6. doi:10.1002/cncr.20043.

39. Fu L, Liu F, Fu H, Liu L, Yuan S, Gao Y, et al. Circulating tumor cells correlate with recurrence in stage III small-cell lung cancer after systemic chemoradiotherapy and prophylactic cranial irradiation. Jpn J Clin Oncol. 2014;44(10):948-55. doi:10.1093/jjco/hyu109.
