# Supplementary material for: Risk Factors for Brain Metastases in Patients With Small Cell Lung Cancer: A Systematic Review and Meta-Analysis
Source: Front Oncol. 2022 Jun 10;12:889161. doi: 10.3389/fonc.2022.889161 (PMC9226404; doi:10.3389/fonc.2022.889161)
Supplement: Supplementary file 9 [file Table_7.docx]

| Appendix table 7. Summary of the 47 risk factors without meta-analysis | | | | |
| --- | --- | --- | --- | --- |
|  |  | BM | | |
|  |  | Risk | Non-significant | Unclear |
| OS | Risk | p-stage, pre-RT KPS,  no treatment vs CRT, | Laterality, | Weight loss, liver metastasis, pre-treatment NLR,  CRT-D, intensity-modulated radiotherapy (IMRT) vs 2D/3D,  PET-CT or not at diagnosis,  EP vs CEV |
|  | Non-significant | LVI,  chemo alone vs CRT, | Race,  chronic disease, histology,  number of metastatic sites,  bone metastasis, adrenal metastasis, lung metastasis, anatomical site, KPS, LDH, NSE, CEA, blood glucose, SUVmax, BED, SER, era, treatment intent curative or not, chemo regimen, brain CT vs MRI before PCI |  |
|  | Unclear | pre-PCI TLC, pretreatment platelet count | BMI, N-stage,  pretreatment TLC, pre-PCI platelet count, | Tumor size, response,  PCI timing,  TRT or not,  TRT timing,  CRT sequences,  TRT fractionation, chemo cycles, chemo or not in resected SCLC |
|  | No information |  | hypertension, number of extrathoracic metastatic sites,  neutrophil count,  pre-PCI NLR, CTC,  hospital. | Surgery; |
| ***Abbreviations:***  BED, biologically effective dose; BM, brain metastasis; BMFS, brain metastasis free survival; BMI, body mass index; CCRT, concurrent chemoradiotherapy; CEA, carcinoembryonic antigen; CEV, cyclophosphamide-epirubicin-vincristine; chemo, chemotherapy; CI, confidence interval; CR, complete response; CRT, chemoradiotherapy; CRT-D: Chemoradiotherapy duration; CT, computerized tomography; CTC, circulating tumor cells; ED, extensive-stage disease; EP, etoposide-platinum; HR, hazard ratio; IMRT, intensity-modulated radiotherapy; IPTW, inverse probability treatment weight; IR, incomplete response; KPS, Karnofsky performance status scale; LD, limited-stage disease; LDH, lactate dehydrogenase; LVI, lymphovascular invasion; MRI, magnetic resonance imaging; NA, not applicable; NI, no information; NLR, neutrophil-to-lymphocyte ratio; NR: Non-response; NSE, neuron-specific enolase; ODRT, once-daily radiotherapy; OR, odds ratio; OS, overall survival; PCI, prophylactic cranial irradiation; PET-CT, positron emission tomography and computed tomography; PLR, platelet-to-lymphocyte ratio; PORT, postoperative radiotherapy; PS, performance status; SCLC, small cell lung cancer; SCRT, sequential chemoradiotherapy; SD, stable disease; SER, start of any treatment until the end of chest irradiation; SHR, subdistribution hazard ratio; SUV, standardized uptake value, tGTV, thoracic gross tumor volume; TRT, thoracic radiotherapy; TDRT, twice-daily radiotherapy; 2D, two-dimensional radiotherapy; 3D, three-dimensional radiotherapy. | | | | |
